# Exocytosis of ATP From Astrocytes Modulates Phasic and Tonic Inhibition in the Neocortex

**DOI:** 10.1371/journal.pbio.1001747

**Published:** 2014-01-07

**Authors:** Ulyana Lalo, Oleg Palygin, Seyed Rasooli-Nejad, Jemma Andrew, Philip G. Haydon, Yuriy Pankratov

**Affiliations:** 1 Faculty of Medical and Human Sciences, The University of Manchester, Manchester, United Kingdom; 2 School of Life Sciences, University of Warwick, Coventry, United Kingdom; 3 Department of Neuroscience, Tufts University School of Medicine, Boston, Massachusetts, United States of America; ICM - Institut du Cerveau et de la Moelle épinière, France

## Abstract

Communication between neuronal and glial cells is important for many brain functions. Astrocytes can modulate synaptic strength via Ca^2+^-stimulated release of various gliotransmitters, including glutamate and ATP. A physiological role of ATP release from astrocytes was suggested by its contribution to glial Ca^2+^-waves and purinergic modulation of neuronal activity and sleep homeostasis. The mechanisms underlying release of gliotransmitters remain uncertain, and exocytosis is the most intriguing and debated pathway. We investigated release of ATP from acutely dissociated cortical astrocytes using “sniff-cell” approach and demonstrated that release is vesicular in nature and can be triggered by elevation of intracellular Ca^2+^ via metabotropic and ionotropic receptors or direct UV-uncaging. The exocytosis of ATP from neocortical astrocytes occurred in the millisecond time scale contrasting with much slower nonvesicular release of gliotransmitters via Best1 and TREK-1 channels, reported recently in hippocampus. Furthermore, we discovered that elevation of cytosolic Ca^2+^ in cortical astrocytes triggered the release of ATP that directly activated quantal purinergic currents in the pyramidal neurons. The glia-driven burst of purinergic currents in neurons was followed by significant attenuation of both synaptic and tonic inhibition. The Ca^2+^-entry through the neuronal P2X purinoreceptors led to phosphorylation-dependent down-regulation of GABAA receptors. The negative purinergic modulation of postsynaptic GABA receptors was accompanied by small presynaptic enhancement of GABA release. Glia-driven purinergic modulation of inhibitory transmission was not observed in neurons when astrocytes expressed dn-SNARE to impair exocytosis. The astrocyte-driven purinergic currents and glia-driven modulation of GABA receptors were significantly reduced in the P2X4 KO mice. Our data provide a key evidence to support the physiological importance of exocytosis of ATP from astrocytes in the neocortex.

## Abstract 2

Astrocytes secrete ATP by exocytosis from synaptic-like vesicles, activating neuronal P2X receptors, which contribute to postsynaptic GABA receptor down-regulation, ultimately mediating the communication between astrocytes and neurons required for brain function.

## Author Summary

Brain function depends on the interaction between two major types of cells: neurons transmitting electrical signals and glial cells, which control cerebral circulation and neuronal homeostasis. There is a growing evidence of the participation of astrocytes in regulating neuronal excitability and synaptic plasticity via the release of “gliotransmitters,” which include glutamate and ATP. The importance of ATP release from astrocytes was suggested by studies that demonstrated its contribution to neuronal activity and sleep homeostasis via modulation of known “purinergic” receptors. But the mechanisms underlying gliotransmitter release and the physiological significance of direct glia-to-neuron communication remain unknown and intensively debated. Here, we investigate the release of ATP from astrocytes of brain neocortex and demonstrate that astrocytes can release ATP by Ca^2+^-dependent exocytosis, most likely from synaptic-like microvesicles. We also find that vesicular release of ATP from astrocytes can directly activate excitatory signaling in the neighboring neurons, operating through purinergic P2X receptors. We saw that activation of these P2X receptors by astrocyte-driven ATP down-regulated the inhibitory synaptic signaling in the neocortical neurons. Our results imply that exocytosis of gliotransmitters is important for the communication between astrocytes and neurons in the neocortex.

## Introduction

ATP acts as neurotransmitter mediating excitatory synaptic transmission and synaptic plasticity in the central nervous system [1],[2]. There is growing evidence that ATP can also play an important role in signal transfer between neuronal and glial circuits and within glial networks [3]–[6]. ATP can regulate growth and development of neural cells and contribute to various pathological processes [7]–[9]. Action of ATP is mediated by ionotropic P2X and metabotropic P2Y receptors abundantly expressed in many types of neurons and glial cells [1],[2]. By virtue of the high Ca^2+^-permeability of P2X receptors and the ability of P2Y receptors to stimulate IP_3_-dependent Ca^2+^ release from endoplasmic reticulum, purinergic receptors can transmit robust Ca^2+^-signals and thereby modulate activity and trafficking of excitatory and inhibitory receptors [10],[11]. In addition to direct actions mediated by P2 purinoreceptors, ATP can initiate secondary neuromodulation via P1 adenosine receptors after rapid degradation by ecto-nucleotidases to adenosine [2],[12]. Different mechanisms of ATP release have been identified, such as vesicular release from nerve terminals [13],[14] and several nonvesicular pathways, including concentration gradient-driven diffusion through gap-junction hemichannels, anion channels, and dilated P2X7 receptors [4],[7],[15],[16].

A physiological role of ATP release from astrocytes has been suggested by the participation of ATP in the propagation of glial Ca^2+^-waves [17]–[19] and significant contribution of ATP and adenosine to the astroglia-driven modulation of neuronal activity and sleep homeostasis [4],[12],[20]. There is growing evidence, albeit obtained mostly in cell cultures, that the release of gliotransmitters may share common mechanisms of vesicular neurotransmitter release such as a dependence on the proton gradient and vesicular transporters, SNARE proteins, andintracellular Ca^2+^ elevation [9],[21],[22]. Importantly, astroglial-driven release of ATP and modulation of synaptic plasticity in the hippocampus were suppressed in transgenic mice expressing a dominant-negative SNARE (dn-SNARE) domain selectively in astrocytes [12].

However, the mechanism of gliotransmitter release from astrocytes has been disputed [16]. Ca^2+^-dependent exocytosis of glutamate and ATP, mainly from cultured hippocampal astrocytes, has been reported [21]–[26]. By contrast, alteration of astroglial InsP3-mediated Ca^2+^-signaling did not have a significant effect on glutamatergic synaptic transmission in the hippocampal slices [27], fuelling the debate on the role for glial exocytosis in more intact tissue [16]. Still, more recent *in situ* and *in vivo* data demonstrated an effect of astroglial InsP3-mediated Ca^2+^-signaling on cholinergic modulation of synaptic plasticity in hippocampus and neocortex [28]–[30]. At the same time, two recent studies reported the possibility of nonvesicular release of astroglial glutamate through the TREK-1 and best1 channels [31] and the lack of immunostaining for vesicular glutamate transporters in brain astrocytes [32], contrasting with the bulk of evidence for glial exocytosis obtained by variety of different techniques [21]–[26],[33]. Thus, physiological relevance of Ca^2+^-dependent exocytosis of gliotransmitters remains controversial.

In this study, to avoid possible artifacts of cell culture, we investigate release of ATP from acutely isolated cortical astrocytes [34] and astrocytes in the neocortical slices. We provide several lines of evidence for (1) existence of functional vesicular mechanism of Ca^2+^-dependent gliotransmitter release in neocortical astrocytes, (2) quantal P2X receptor-mediated currents directly activated in neocortical neurons by release of ATP from astrocytes, and (3) glia-driven purinergic modulation of GABAergic transmission that is impaired by astrocytic expression of dn-SNARE or deletion of P2X4 receptors.

## Results

### Vesicular Release of ATP From Acutely Isolated Cortical Astrocytes

As a first step in demonstrating the presence of quantal Ca^2+^-dependent release of ATP from astrocytes, we used a sniffer cell approach where ATP release from astrocyte was detected by HEK293 expressing P2X2 receptors (see Materials and Methods for details). We compared release of ATP from astroglia of somatosensory cortex of wild-type mice and from mice conditionally expressing the SNARE domain of VAMP2 selectively in astrocytes (dn-SNARE mice) [12],[20].

**Figure 1:**
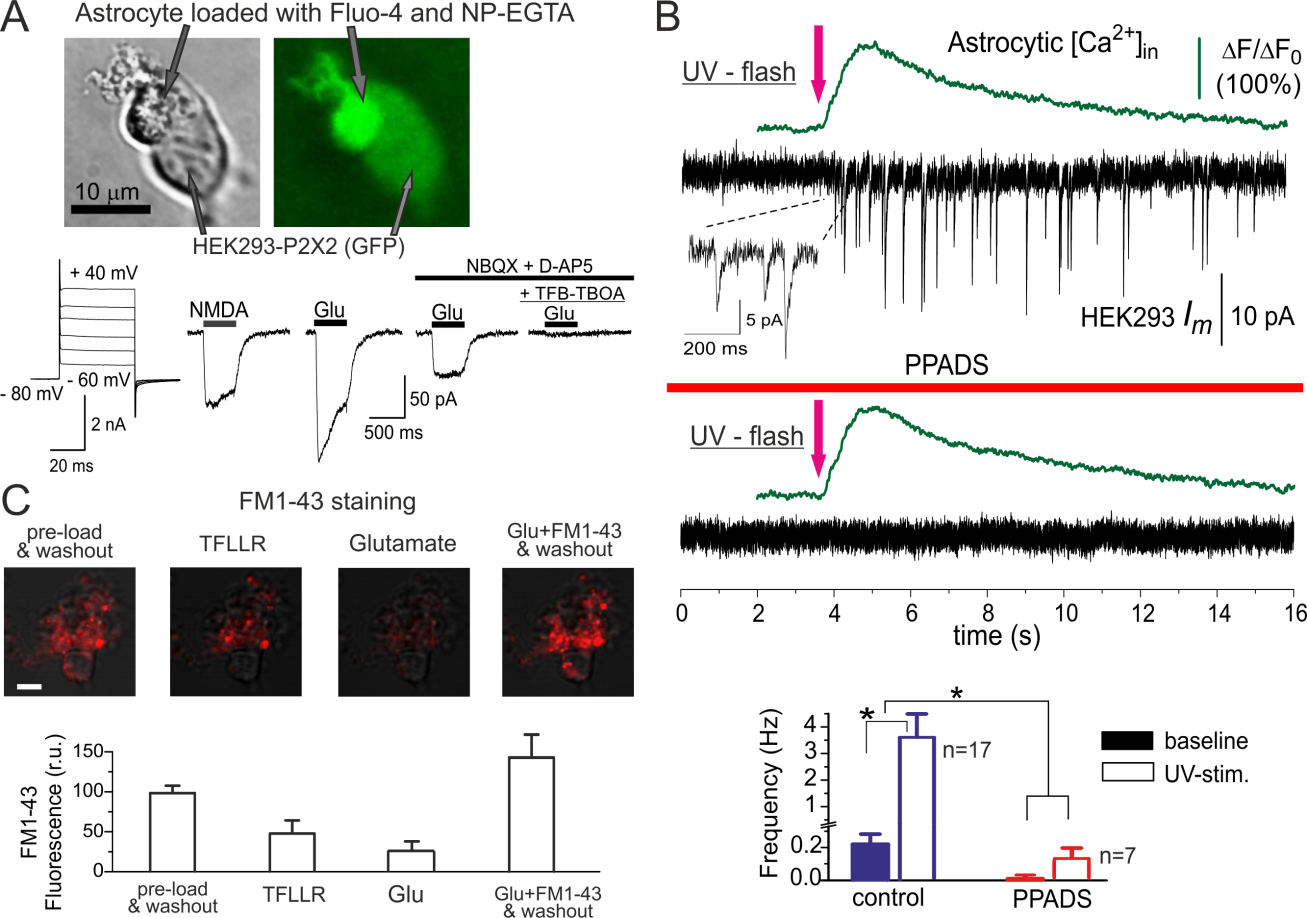
Detection of ATP released from cortical astrocytes with the aid of sniffer cells. (A) Astrocytes acutely dissociated from the wild-type mouse neocortex have been loaded with UV-photoliable Ca^2+^-chelator NP-EGTA and Ca^2+^-indicator Fluo4-AM and placed over HEK293 cells expressing P2X2 receptors. The graphs below show the depolarization-activated currents and responses to application of 20 µM NMDA and 100 µM glutamate recorded in the astrocyte at a holding potential of −80 mV after an uncaging experiment; the response recorded under NBQX and D-AP5 was mediated by glutamate transporters, as evidenced by inhibition with specific blocker of glial glutamate transporters TFB-TBOA (300 nM). (B) Fluo-4 fluorescence was monitored in the astrocyte simultaneously with recording of transmembrane current in the HEK293-P2X2 cell voltage-clamped at −80 mV. UV uncaging of Ca^2+^ in the astrocyte was followed by the burst of phasic currents in the HEK293-P2X2 cell. Both the baseline and UV-elicited phasic currents were strongly inhibited after application of P2X receptor antagonist PPADS, confirming that they were mediated by ATP receptors. The diagram in the bottom panel shows the amplitude and frequency (mean ± SD for indicated numbers of HEK293-P2X2 cells) of phasic currents; statistical significance of the effect of PPADS was as indicated (*p<0.02 and **p<0.005, ANOVA). (C) Staining of cortical astrocyte with FM1-43 supports the existence of the mechanism of vesicular release. Acutely isolated cortical astrocytes were pre-incubated with 2.5 µM FM1-43 for 15 min and then washed out with extracellular medium for 15 min. The upper row shows the superposition of the DIC image of astrocyte and two-photon images of FM1-43 fluorescence (maximal intensity projections of Z-stack) recorded before (control) and after a 5-min-long application of the agonist of astroglial PAR-1 receptors TFLLR (10 µM) [27] and glutamate; scale bar is 5 µm. Activation of astrocytes via PAR-1 and glutamate receptors led to FM1-43 destaining. To recover fluorescence, FM1-43 was applied for 5 min in the presence of glutamate (100 µM) and then washed out for 15 min. The bottom plot shows the changes in the FM1-43 fluorescent signal (mean ± SD for seven astrocytes) averaged over the whole-cell image.

Acutely isolated cortical astrocytes were separately loaded with Ca^2+^-indicator Fluo-4 and photosensitive Ca^2+^-chelator NP-EGTA [35] and then distributed into a recording chamber containing preplated HEK293-P2X2 cells (Figure 1). Whole-cell voltage-clamp recordings were made from a HEK293-P2X2 cell lying in the immediate vicinity to astrocytes (Figure 1A). Identification of astrocytes was confirmed by their functional characterization at the end of experiment, including low input resistance, lack of voltage-gated Na^+^-conductance, large K^+^-conductance, large conductance mediated by glutamate transporters, and NMDA receptors lacking Mg^2+^-block (Table S1). Thus, influence of nonastrocytic cells in the experiments reported below can be ruled out.

Brief flash of UV-light caused the uncoupling of Ca^2+^ from NP-EGTA (monitored by Fluo-4 Ca^2+^-indicator). This was accompanied by an asynchronous burst of phasic currents in the adjacent ATP-sensitive sniffer cell in 17 of 20 experiments (Figure 1B). P2 receptor antagonist PPADS (10 µM) prevented the detection of UV-evoked phasic currents (*n* = 7 of 7; Figure 1B), confirming that they were mediated by ATP acting via P2X receptor.

Some phasic currents were observed in the sniffer cells having astrocytes laying on their surface even before triggering the Ca^2+^-rise in the astrocyte, but at much lower frequency (Figure 1B). These baseline events were not detected in the absence of astrocytes in any of 10 native HEK293-P2X2 cells tested. Application of PPADS (10 µM) reduced the frequency of baseline and UV-evoked events correspondingly by 94%±3% and 97%±3% (Figure 1C), confirming the purinergic nature of all phasic currents. An exocytotic mechanism of ATP release was suggested by the activity-dependent staining of astrocytes with vesicular marker FM1-43 (Figure 1C).

**Figure 2:**
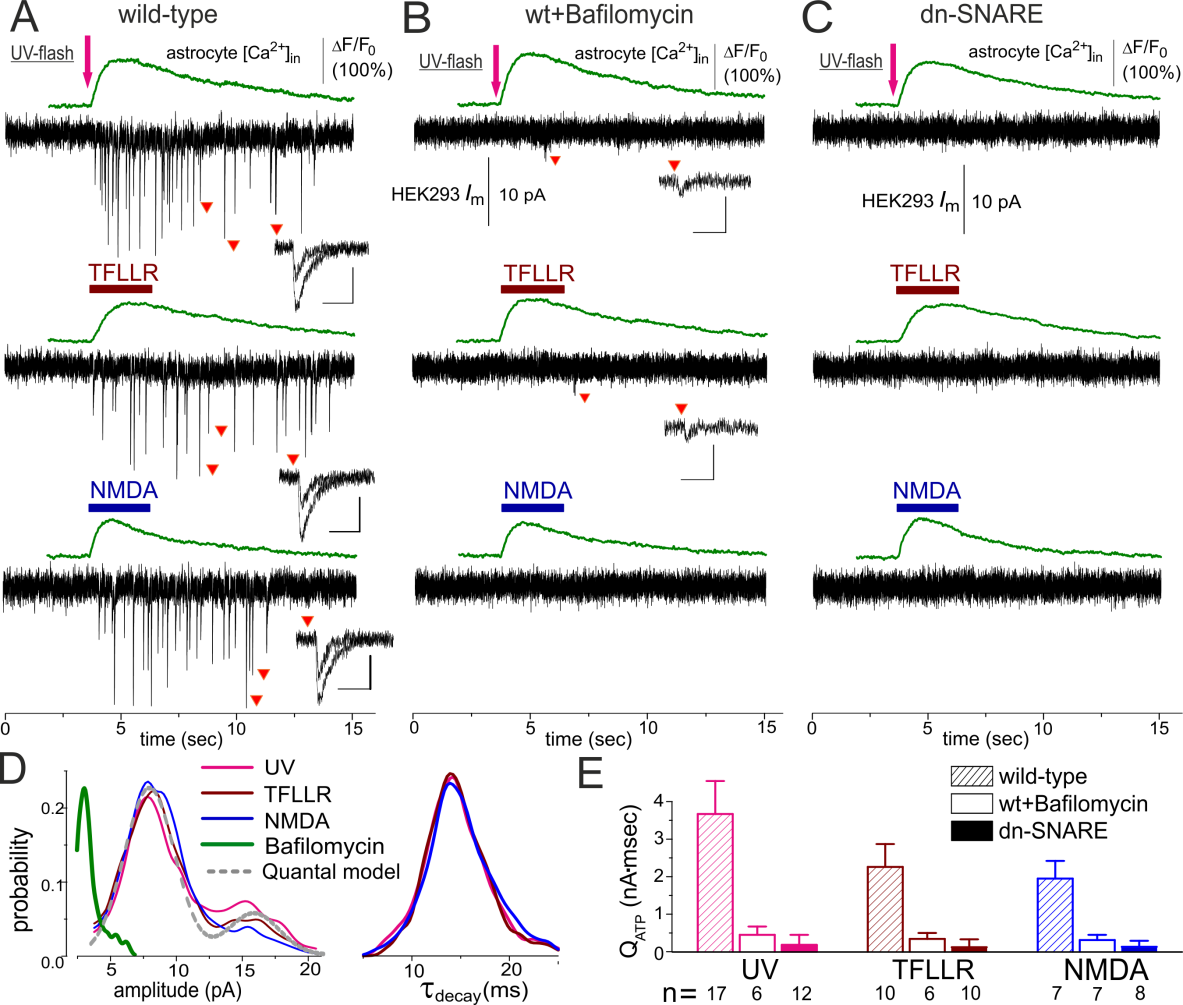
Ca^2+^-dependent release of ATP can be evoked in the cortical astrocytes of the wild-type but not of the dn-SNARE mice. Release of ATP from cortical astrocytes of wild-type (A, B) and dn-SNARE mice (C) was detected using the sniffer cells as described in Figure 1. (A) Elevation of cytosolic Ca^2+^ level was elicited in the astrocytes by UV uncaging and by rapid application of the agonist of PAR-1 metabotropic receptor TFLLR (10 µM) or the agonist of glutamate ionotropic receptor NMDA (20 µM). (B) Inhibition of vacuolar H-ATPase in the astrocytes with Bafilomycin A1 (1 µM for 2 h) dramatically decreased both the amplitude and frequency of phasic currents. (C) Elevation of the Ca^2+^ level in any of the dn-SNARE astrocytes did not lead to activation of phasic purinergic currents in the sniffer cell. Inlays in (A–C) show examples of individual phasic currents recorded at moments indicated; scale bars are 50 ms and 10 pA. (D) The amplitude and decay time distributions of purinergic currents recorded in the HEK293-P2X2 cells after stimulation of the astrocytes; data were pooled for number of experiments indicated in (E). The grey dotted line shows the best fit of quantal model to the distribution of UV-activated currents. (E) The pooled data (mean ± SD for indicated numbers of experiments) on net release of ATP were assessed as total charge transferred by spontaneous currents in the sniffer cell. The effects of bafylomicin and dn-SNARE expression on net charge transferred by purinergic currents were statistically significant at *p* = 0.005 (two-population *t* test).

To further test the role of an elevation of cytosolic Ca^2+^ concentration as a trigger for the release of ATP from astrocytes, we applied agonists of astroglial metabotropic PAR-1 receptors (see also Figure S1) [35],[36] and ionotropic NMDA receptors [6],[24]. The PAR-1 receptors were chosen as an ample method of astrocyte activation because of the ability to activate IP3 pathway and their predominant expression in glial but not in neuronal cells [35],[36]. Similar to UV-uncaging, activation of cytosolic Ca^2+^-transients in the astrocyte either by application of the PAR-1 agonist TFLLR (10 µM; *n* = 10) or by application of NMDA (20 µM; *n* = 7) elicited bursts of spontaneous currents in the adjacent ATP-sensitive sniffer cells (Figure 2A). TFLLR and NMDA did not evoke any activity in the HEK293-P2X2 cells when applied in the absence of astrocytes (*n* = 5 agonist, unpublished data). The phasic currents in the sniffer cells had amplitudes of 11.2±2.4 pA (*n* = 34) and rise and decay time of 1.6±0.5 ms and 15.2±3.9 ms correspondingly (Figure 2D), thus resembling parameters of purinergic synaptic currents [13],[14].

As a test of a vesicular mechanism of ATP release, we isolated astrocytes from cortical slices pretreated with blocker of vacuolar-type H-ATPase bafilomycin A1 (1 µM) for 2 h. Treatment with bafilomycin caused a decrease in the amplitudes and frequency of phasic currents initiated by Ca^2+^-elevation in the astrocytes (Figure 2B). The mean amplitude of phasic purinergic currents activated in the sniffer cell by stimulation of bafilomycin-treated astrocytes was only 3.1±0.4 pA (*n* = 19). The overall charge transferred by phasic currents activated after stimulation of bafilomycin-treated astrocytes by UV, TFLLR, and NMDA was 9.7%±4.2% (*n* = 7), 11.8%±5.6% (*n* = 5), and 12.6%±5.8% (*n* = 7) of the corresponding control values (Figure 2E).

In support of exocytotic mechanism of ATP release, astrocytes obtained from dn-SNARE mice elicited a diminished burst of purinergic currents in sniffer cells regardless of the method used to elevate cytosolic Ca^2+^-level (Figure 2C,E). We also observed the SNARE-complex-dependent release of ATP from the isolated hippocampal astrocytes (Figure S2).

The amplitude histograms of the phasic P2X-mediated currents activated by elevation of astrocytic Ca^2+^ exhibited prominent second peak (Figure 2D; see also Figure S3) at amplitude twice that of the primary peak. Fitting of amplitude distribution with simple multiquantal binomial model (shown in Figure 2D as dotted line) gave a quantal size of 7.9±0.13 pA and release probability of 0.28±0.04. Similarly, fitting of the distributions of P2X-currents evoked in sniffer cell after application of NMDA and TFLLR gave quantal size of 8.14±0.15 pA and 8.05±0.11 pA, respectively.

It should be noted that astrocyte-driven purinergic currents observed in our experiments had much faster kinetics (10–25 ms) than nonvesicular release of gliotransmitters from hippocampal astrocytes, which was mediated by TREK-1 potassium channels and best1 chloride channels [31]. To verify the lack of contribution of nonvesicular mechanisms to the quantal purinergic phasic currents in the sniffer cells, we activated astrocytes by TFLLR in presence of TREK-1 channels inhibitor fluoxetine [37] and large conductance chloride channels inhibitors DIDS and NPPB [38]. Application of fluoxetine (100 µM), DIDS (300 µM), and NPPB (100 µM) did not have marked effect on the astrocyte-evoked phasic currents in the HEK293 cells in any of five experiments for each inhibitor (Figure S4).

Combined, the above results strongly suggest that activity-dependent release of ATP from cortical astrocytes occurs mainly via quantal exocytotic mechanism, dependent on SNARE protein complex.

Vesicular mechanism of ATP release from neocortical astrocytes was also supported by immunostaining of living isolated astrocytes with antibodies to vesicular nucleotide transporter VNUT1 and various vesicular, neuronal, and glial marker proteins (Figures S5 and S6). Although immunostaining of live cells has certain limitations (see Text S1) and should be interpreted with great caution, our data suggest the good co-localization of VNUT1 and synaptic vesicle (SV) markers (Figure S5A), which is in agreement with previous reports of vesicular location of VNUT1 [26] and presence of synaptic-like vesicles in astrocytes [22],[24]. We observed weaker correlation between VNUT1 and lysosomal markers cathepsin D and LAMP3 (Figure S5B,C), which goes in line with previous observation of astroglial ATP release by the lysosome exocytosis [23].

However, lysosomal exocytosis from astrocytes exhibited much slower kinetics [23] than the purinergic currents measured in the sniffer cells (Figures 1 and 2). This argues against a major contribution of this mechanism to the present observations. As the kinetics of sniffer cell responses are more consistent with millisecond time-scale of SV exocytosis from astrocytes [24], we suggest that the astrocyte-driven purinergic currents observed in our experiments could be triggered by exocytosis of ATP from synaptic-like vesicles. We also observed an immunoreactivity for vesicular glutamate transporter (VGLUT1) in the fraction of cortical astrocytes (Figure S5D,E; Figure S6A), which goes in line with data reported previously for hippocampal and cortical astrocytes [22],[33]. Of course, further investigation of exocytosis of glutamate from neocortical astrocytes is required, which is beyond the scope of this article.

### Quantal Release of ATP From the Cortical Astrocytes in Situ

**Figure 3:**
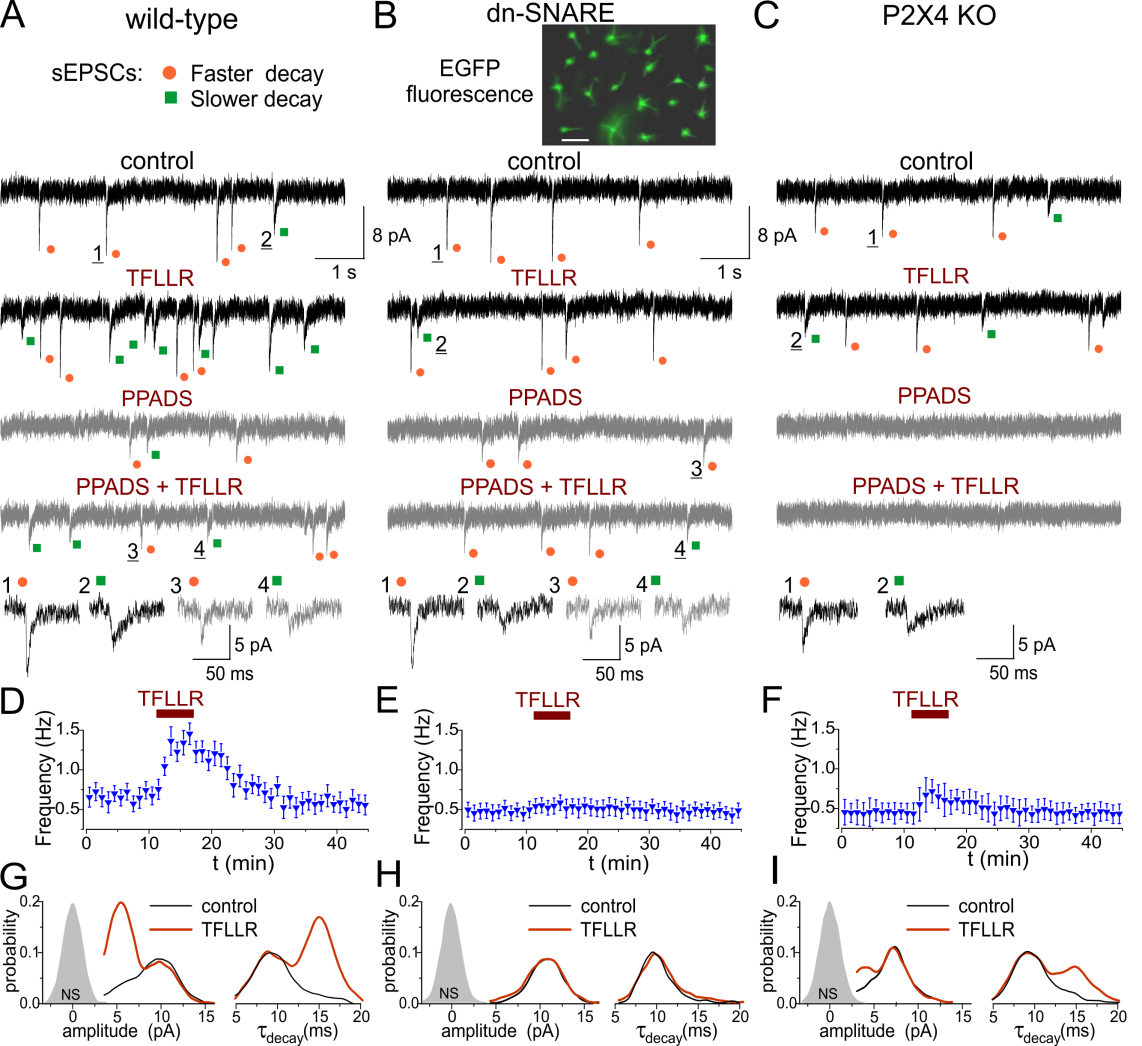
Quantal release of ATP from astrocytes triggers fast purinergic currents in the cortical neurons *in situ*. The spontaneous transmembrane currents were monitored in the layer 2/3 pyramidal neurons of neocortical slices of wild-type (A, D, G), dn-SNARE (B, E, H), and P2X4 KO mice (C, F, I). Expression of dn-SNARE in the astroglial cells of the neocortex is confirmed by EGFP-GFAP fluorescence (right column, scale bar 20 µm; see also Figure S15). Two distinct populations of spontaneous sEPSCs were recorded in the pyramidal neurons in the presence of TTX (1 µM), CNQX (50 µM), and D-APV (30 µM) at a holding potential of −80 mV: currents with larger amplitude and fast kinetics (orange dots) and currents with smaller amplitude and slow kinetics (green dots). (A, B, C) The representative transmembrane currents were recorded before and after activation of Ca^2+^ signaling in the astrocytes by selective agonist of astroglial PAR-1 receptor. The nonglutamatergic inward spontaneous currents (sEPSCs) were inhibited by P2 receptors antagonist PPADS (10 µM) and knocking out of P2X4 receptors. (D, E, F) Time course of changes in the frequency of sEPSCs currents during application of TFLLR (10 µM). Each dot represents the frequency calculated for a 1 min time window, and data show mean ± SD for number of neurons as follows: 17 (WT), 8 (dn-SNARE), and 12 (P2X4 KO). Activation of the PAR-1 receptor caused the appearance of a large number of spontaneous currents in the neurons of wild-type but not dn-SNARE mice; the frequency of sEPSCs was significantly lower in the P2X4 KO mice. (G, H, I) The amplitude and decay time distributions of purinergic sEPSCs recorded before and after application of TFLLR reveal the presence of a distinct population of spontaneous currents of smaller amplitude and slower kinetics in the wild-type but not in the dn-SNARE mice. Shaded areas show the amplitude distribution of background noise. Stimulation of astrocytes with TFLLR significantly increases the peaks corresponding to smaller and slower sEPSCs only in the neurons of the wild-type mice, whereas currents recorded in the neurons of dn-SNARE mice exhibit single-peaked amplitude and decay distributions. Deletion of P2X4 receptors attenuated amplitudes of both fast and slow inward currents and significantly reduced the proportion of slower currents evoked by TFLLR application.

Previously we have shown that cortical pyramidal neurons express ionotropic P2X purinoreceptor, which can be activated by synaptic release of ATP [13]. Hence, it might be plausible to detect the glia-driven contribution to purinergic current in neurons. We recorded whole-cell currents in the pyramidal neurons of neocortical layer 2/3 of brain slice at membrane potential of −80 mV in the presence of glutamate receptor antagonists CNQX (50 µM) and D-APV (30 µM) and irreversible blocker of GABA receptors picrotoxin (100 µM). Like our previous results [13], we observed residual nonglutamatergic excitatory spontaneous synaptic currents (Figure 3A,B); neither amplitude nor frequency of residual currents were affected by further increase in concentrations of glutamate and GABA receptors antagonists (*n* = 10 cells tested, unpublished data).

The amplitude of inward spontaneous excitatory currents (sEPSCs) was reduced by specific antagonists of P2X receptors PPADS (10 µM) and NF-279 (3 µM) correspondingly by 45%±1 3% (*n* = 7) and 56%±19% (*n* = 16); the sEPSC frequency was reduced by PPADS and NF-279 by 65%±22% and 69%±27%, respectively (Figures 3 and S7). At concentrations used, both PPADS and NF279 are selective for P2X receptors [39]–[41]. Based on these data as well as our previous work [11],[13], the spontaneous inward currents observed in cortical neurons in the presence of glutamatergic and GABAergic antagonists can be confidently attributed to the ATP receptors.

The partial inhibitory action of PPADS and NF-279 on nonglutamatergic sEPSCs could be explained by participation of homomeric P2X4 receptors, which are insensitive to these antagonists [1],[41],[42]. Since P2X4 subunit-containing receptors are abundantly expressed in the brain and could potentially contribute to neuronal purinergic signaling [1],[42]–[44], we used previously characterized P2X4 receptor knockout mice (P2X4 KO) [38]. In the P2X4 KO mice, application of 10 µM PPADS decreased the amplitude and frequency of nonglutamatergic sEPSCs (Figure 3C) by 74%±10% and 97%±6% correspondingly (*n* = 12); difference from the wild-type mice was statistically significant with *p* = 0.05 and 0.01. Taking into account that significant attenuation of sEPSCs can put them below the detection threshold, nonglutamatergic sEPSCs can be confidently attributed to neuronal P2X receptors.

We triggered the release of gliotransmitters from the astrocytes by rapid application of an agonist of PAR-1 receptor TFLLR to neocortical slices. As in hippocampus [35],[36], TFLLR (10 µM) triggered cytosolic Ca^2+^ rise predominantly in astrocytes (Figure S1). Application of TFLLR caused a dramatic increase in the frequency of ATP-mediated spontaneous currents (Figure 3A,D). TFLLR also elevated astrocytic Ca^2+^ in dn-SNARE mice, but the burst of purinergic spontaneous currents was not detected (Figure 3B,E). In the P2X4 knockout mice, the average increase in the purinergic sEPSCs frequency reached only 28%±15% (*n* = 12), which was significantly lower (*p*<0.005) than 72%±21% increase observed in wild-type mice (Figure 3C,F). Combined, these results demonstrate that activation of astrocytes can evoke synaptic-like purinergic currents in neurons. In addition to effects of knocking out and inhibiting P2X receptors, the purinergic nature of glia-driven spontaneous currents was corroborated by inhibitory action of ATP-hydrolyzing enzyme apyrase (Figure S8). The apyrase application significantly decreased the mean amplitude and frequency of sEPSCs and abolished the TFLLR-induced burst. The decrease in the sEPSCs frequency is most likely related to the reduction of their amplitude below threshold of detection.

**Figure 4:**
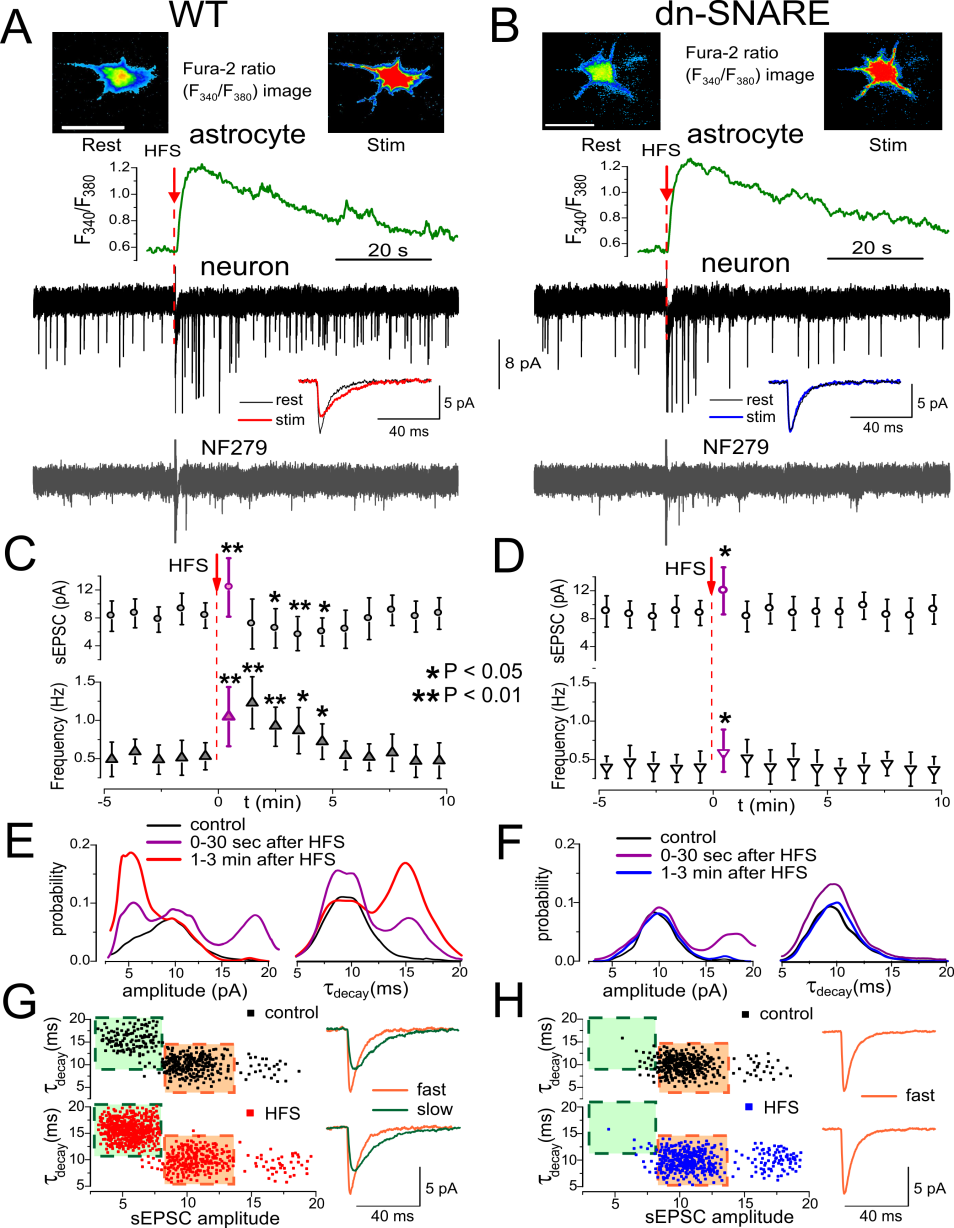
Exocytosis of ATP from astrocytes *in situ* can be triggered by synaptic stimulation. (A, B) Ca^2+^ signaling was monitored in astrocytes of somatosensory cortex layer 2/3 of wild-type (A) and dn-SNARE (B) mice simultaneously with voltage-clamp recordings of membrane currents in the pyramidal neurons. Spontaneous currents recorded in the pyramidal neurons at a holding potential of −80 mV in the presence of picrotoxin (100 µM) and CNQX (50 µM) were mediated by P2X receptors, as verified by inhibition with selective P2X antagonist NF279 (3 µM). The single episode of 100 Hz stimulation (HFS) triggered Ca^2+^ transients in the astrocytes of both wild-type and dn-SNARE mice; representatives are the Ca^2+^ transients and pseudo-color images (scale bar, 10 µm) recorded before (rest) and at the peak of response (stim). Ca^2+^ transients were followed by burst of spontaneous purinergic currents only in the neurons of wild-type mice (A), whereas neuronal spontaneous activity was not enhanced in the dn-SNARE mice (B). Inlays show the average waveforms (20 events) of spontaneous currents recorded before (rest) and 1 min after HFS (stim). (C, D). Each dot shows the average amplitude and frequency of spontaneous currents recorded in a 1 min time window in the pyramidal neurons of wild-type and dn-SNARE mice; data are presented as mean ± SD for six neurons. The asterisks (*) and (**) indicate the significant difference from the control values. The decrease in amplitude and significant increase in frequency of purinergic sEPSCs were observed in the wild-type but not in the dn-SNARE mice. (E, F) The amplitude and decay time distributions of purinergic sEPSCs recorded before, immediately after (0–30 s), and 1–3 min after HFS (pooled data for six neurons of each type) reveal the presence of a distinct population of spontaneous currents of smaller amplitude and slower kinetics in the wild-type but not in the dn-SNARE mice. Hence, these events are most likely activated by ATP released from astrocytes. The faster sEPSCs with larger amplitudes that underlie baseline activity both in wild-type and dn-SNARE mice are activated by ATP released from the nerve terminals. (G, H) The plot of decay time of purinergic sEPSCs against the amplitude demonstrates the presence of two populations of sEPSCs in the wild-type mice: slower currents of smaller amplitude (green area) and faster currents of higher amplitude (orange area). The corresponding waveforms (average of 20 traces) are shown in the inlay. HFS significantly increases the number of slower spontaneous currents. These currents were very rarely observed in the dn-SNARE mice, both in control and after HFS.

In the wild-type mice, purinergic sEPSCs showed bimodal amplitude distributions (Figure 3G–I; see also Figure S9) with peaks at 3.1±0.9 pA and 5.7±1.6 pA (*n* = 14); decay time distributions had peaks at 9.1±0.9 ms and 15.3±1.8 ms (Figure 3G). TFLLR selectively increased the probability of detection of smaller and slower sEPSCs in all 14 neurons tested. In contrast, recordings made from cortical neurons of dn-SNARE mice did not show two peaks in the distributions of amplitude or decay time; the amplitude and decay time were not altered after TFLLR application (Figure 4H). In the P2X4 KO mice, the amplitude distribution of purinergic sEPSCs showed peaks as 2.5±0.7 pA and 4.2±1.1 pA; decay time distribution had peaks at 9.0±0.9 and 15.7±1.7 in control. Activation of astrocytes caused just a moderate increase in the proportion of smaller and slower sEPSCs in the P2X4 KO neurons (Figure 3I).

Elimination of the distinct population of smaller and slower currents by astrocytic dn-SNARE expression strongly suggests that this population of purinergic currents was elicited by exocytosis of ATP from astrocytes. The vesicular origin of slower purinergic sEPSCs was also supported by elimination of these events by treatment of the cortical slices with 1 µM bafilomycin A1 (Table S2). The slower and faster purinergic currents recorded in cortical neurons exhibited different quantal behavior (Figure S7). The slower purinergic currents evoked by application of TFLLR in the presence of TTX (Figure S3A) exhibited multiquantal amplitude distribution, whereas faster currents exhibited monoquantal distribution typical for miniature synaptic currents (Figure S7B,C).

Thus, detailed analysis of purinergic sEPSCs in the pyramidal cortical neurons revealed two distinct populations of events, which differ by their amplitude and kinetics. Based on their insensitivity to astrocytic dn-SNARE expression, larger and faster sEPSCs most likely have a neuronal origin. In contrast, sEPSCs of smaller amplitude and slower kinetics can be attributed to the vesicular release of ATP from astrocytes. In the following sections, we provide further experimental support of this notion.

### Evaluation of ATP Release in the Neocortex With Microelectrode Biosensors

We sought to obtain a parallel line of evidence for the vesicular ATP release from cortical astrocytes via an alternative approach: we measured ATP concentration in neocortical slice using microelectrode biosensors (see Materials and Methods), a technique that has been applied previously for evaluation of ATP release in several brain areas [7],[19]. Selective activation of astrocytic PAR-1 receptor by TFLLR induced a robust increase in the extracellular ATP concentration in the cortical tissues of wild-type mice; this increase was impaired in the dn-SNARE mice and was blocked by bafilomycin, confirming its astroglial origin and vesicular nature (Figure S10A). The increase in the “tonic” concentration of extracellular ATP after activation of astrocytes in the wild-type mice reached 1.1±0.4 µM (Figure S10B) and was inhibited by 84%±5% (*n* = 7) after incubation with bafilomycin. The TFLLR-evoked elevation of ATP concentration in the dn-SNARE mice was decreased by 56%±12% (*n* = 12) as compared to wild-type. These results support the significant contribution of vesicular mechanism to the activity-dependent release of ATP from cortical astrocytes. Taking into account that bafilomycin can inhibit only re-charging of ATP-storing vesicles and not all the cortical astrocytes express dn-SNARE protein, one could not expect the full inhibition of vesicular ATP release in these experiments. Thus, the incomplete inhibition of TFLLR-evoked ATP transients in the dn-SNARE mice and in the bafilomycin-treated neocortical slices of wild-type mice could hardly be attributed to a large contribution of nonvesicular release.

### Neuronal Activity Causes Vesicular Release of ATP From the Cortical Astrocytes *in Situ*

We have shown previously that stimulation of intracortical afferents is able to significantly elevate cytosolic Ca^2+^-level in the cortical astrocytes acting via ionotropic and metabotropic receptors to glutamate and ATP [5],[6],[34]. We asked whether an episode of high-frequency stimulation (HFS) could similarly trigger release of ATP from astrocytes *in situ*. As before, we monitored spontaneous purinergic currents in the pyramidal cortical neurons at a membrane potential of −80 mV in the presence of CNQX and picrotoxin (Figure 4). The short HFS train triggered more than a 2-fold elevation of the frequency of purinergic sEPSCs in the pyramidal neurons of wild-type mice (Figure 4A,C). Such an elevation did not occur in the dn-SNARE mice, where HFS train caused only a modest transient increase in the sEPSC frequency (Figure 4B,D).

**Figure 5:**
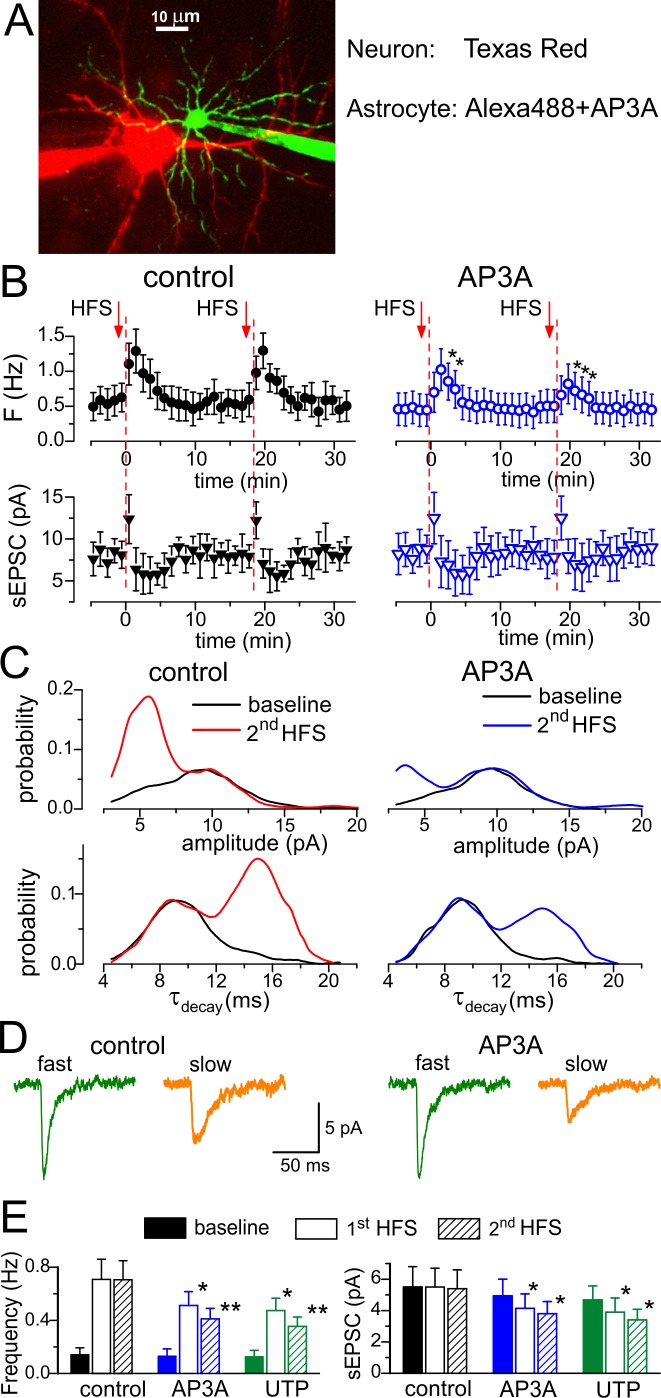
Intracellular perfusion of astrocyte with inhibitors of vesicular transport of ATP selectively affects slower purinergic EPSCs. Whole-cell recordings of spontaneous EPSCs in the pyramidal neurons of somatosensory cortex of wild-type mice were carried out simultaneously with intracellular perfusion of astrocyte located in close proximity. Spontaneous currents recorded in the pyramidal neurons at a holding potential of −80 mV in the presence of picrotoxin (100 µM), CNQX (50 µM), and intracellular MK801 (10 µM) were mediated by P2X receptors, as demonstrated in Figures 3 and 4; the intracellular solution contained fluorescent dye Texas Red (30 µM). Whole-cell recording configuration was also established for neighboring astrocytes 15 min prior to the start of recording in the neuron, and the holding potential was −80 mV. Intracellular solution contained either fluorescent dye AlexaFluor 488 (30 µM) alone or AlexaFluor 488 and 1 mM of diadenosine-triphosphate (AP3A) or UTP. (A) A representative two-photon image of the recording outline and green and red fluorescence images (projection of Z-stack) are merged. (B) Two episodes of 100 Hz stimulation (HFS) of cortical afferents were delivered to trigger Ca^2+^ transients in the astrocytes, as demonstrated in Figure 4. Each dot shows the average amplitude and frequency of spontaneous currents recorded in a 1 min time window in the pyramidal neurons during perfusion of astrocyte with AlexaFluor 488 only (contro) and AlexaFluor and AP3A; data are presented as mean ± SD for five neurons. The asterisk (*) indicates the significant difference (*p*<0.05, unpaired *t* test) from the control values. The decrease in amplitude and significant increase in frequency of purinergic sEPSCs were attenuated by perfusion of astrocyte with AP3A, and the effect was more prominent after the second HFS episode. (C) The amplitude and decay time distributions of purinergic sEPSCs recorded before and 1–3 min after the second HFS (pooled data for five neurons in each case) reveal the decrease in number of sEPSCs of smaller amplitude and slower kinetics after perfusion of astrocyte with AP3A. (D) The representative average waveforms (20 events) of fast and slow spontaneous sEPSCs (separated as shown in Figures 4 and S3) recorded 1 min after the 2-s HFS episode. Note the decrease in the amplitude of slow sEPSCs recorded in the presence of AP3A and lack of changes in the fast sEPSC. (E) Diagrams show the amplitude and frequency of slow purinergic sEPCS averaged within a 3 min time window before (baseline) and after HFS episodes delivered in control and during perfusion of astrocytes with AP3A and UTP. Data are shown as mean ± SD for the five neurons. The statistical significance of the difference from the control values was indicated as (*) *p*<0.05 and (**) *p*<0.01. Note the decrease in the mean frequency and amplitude of slow sEPSCs.

The HFS-induced changes in the amplitude and kinetics of phasic purinergic currents had a complex pattern in the wild-type mice (Figure 4C,E). During the first 30 s after HFS train, the average amplitude of events increased to 12.4±4.2 pA (*n* = 6) as compared to 8.6±2.4 pA in the baseline conditions and their decay time was slightly larger (11.2±2.3 ms, *n* = 6) than in control (9.9±2.7 ms). The sEPSCs appeared in the neurons of wild-type mice 1–3 min after HFS train had lower amplitudes (6.8±1.6 pA, *n* = 6) and much larger decay times (13.4±3.6 ms) than in control. Analysis of the amplitude and decay time distributions (Figure 4E) revealed a significant increase in the number of smaller, slower sEPSCs after HFS train that formed the distinct fraction of purinergic spontaneous events. In addition to smaller and slower currents, the number of fast sEPSCs with fast decay times (9.2±2.5 ms) and large amplitudes (19.6±2.7 pA) was observed during the first 30 s immediately after stimulation (Figure 5E, purple lines). The amplitudes of these large currents corresponded to the double of unitary amplitude of fast purinergic currents; this explains a short-lived increase in the average amplitude immediately after HFS train.

The existence of two functionally distinct populations of purinergic events in the wild-type mice was corroborated by correlation between amplitude and decay time of sEPSCs (Figure 4G). The slower currents (decay time of 15.4±2.2 ms) had smaller amplitudes (5.5±1.3 pA, *n* = 6), but the faster currents (decay time of 9.2±1.3 ms) had higher amplitude (9.9±2.4 pA, *n* = 6). The amplitudes of slower currents closely agree with quantal amplitude of TFLLR-evoked slow purinergic currents recorded at −80 mV, whereas quantal amplitude of fast currents is close to the unitary size of TFLLR-insensitive fast purinergic sEPSCs (Figure S6B). The train of HFS significantly increased the number of slower spontaneous currents with smaller amplitude (Figure 4G).

In contrast to wild-type mice, the only effect produced by HFS train on purinergic sEPSCs in the dn-SNARE mice was the transient increase in the number of double-quantal fast currents (Figure 4D,F), which led to the brief increase in the average amplitude. The expression of dn-SNARE in astrocytes caused a selective loss of the smaller and slower sEPSCs (Figure 4H). These data strongly support the different origins of fast and slow purinergic sEPSCs, from neuronal terminals and astrocytes correspondingly.

To verify that slower purinergic sEPSCs originated from astrocytic ATP release directly, we tested the effect of diadenosine triphosphate (AP3A) and UTP, which have been shown presviously to strongly inhibit transport of ATP into astrocytic vesicles [26]. Since these substance are not specific VNUT antagonists and can have an action on purinergic receptors, we applied them intracellulary to minimize side effects. In order to increase the impact of single-cell perfusion, we chose neuron-astrocyte pairs lying in a close proximity (Figure 5A). A similar strategy has been previously used to test the effects of perfusion of Ca^2+^-chelators into astrocytes [45]. The feasibility of this approach is based on the high probability of synapses of a single neuron falling within functional island enwraped and controlled by single nearby astrocyte [46]. When astrocytes of wild-type mice were perfused only with fluorescent dye, two consecutive HFS episodes caused the burst of slow purinergic sEPSCs in the neighboring neurons (Figure 5B) of the magnitude similar to previous experiments with intact astrocytes (Figure 4). Prolonged intracellular perfusion of astrocyte with 1 mM of AP3A or UTP significantly attenuated the frequency of purinergic sEPSCs (Figure 5B–E) in 10 of the 12 experiments. The effect was more prominent after the second HFS episode. This was most likely related to the depletion of the releasable pool of ATP in astrocytes. Analysis of amplitude and decay time distributions showed that inhibitors of vesicular nucleotide transporters selectively affected the fraction of slower sEPSCs (Figure 5C), significantly decreasing their amplitude and frequency (Figure 5D). Although slower purinergic sEPSCs were not abolished completely, this might be explained by incomplete perfusion of distal astrocytic processes or release from other astrocytes. These results strongly suggest that smaller and slower purinergic sEPSCs originated directly from vesicular release of ATP from neighboring astrocytes.

In summary, our data provide compelling evidence that quantal release of astrocytic ATP activates a distinct population of purinergic currents in cortical pyramidal neurons.

### Postsynaptic Action of Astroglial ATP: Modulation of Postsynaptic GABAA Receptors in the Neocortical Neurons

ATP release from astrocytes can activate neuronal P2X and P2Y receptors. The following increase in cytosolic Ca^2+^-signals may trigger a variety of intracellular cascades implicated in the modulation of synaptic strength [1],[2],[11]. In particular, phosphorylation of postsynaptic GABAA receptors might provide an endogenous pathway for Ca^2+^-dependent regulation of synaptic strength [47],[48].

**Figure 6:**
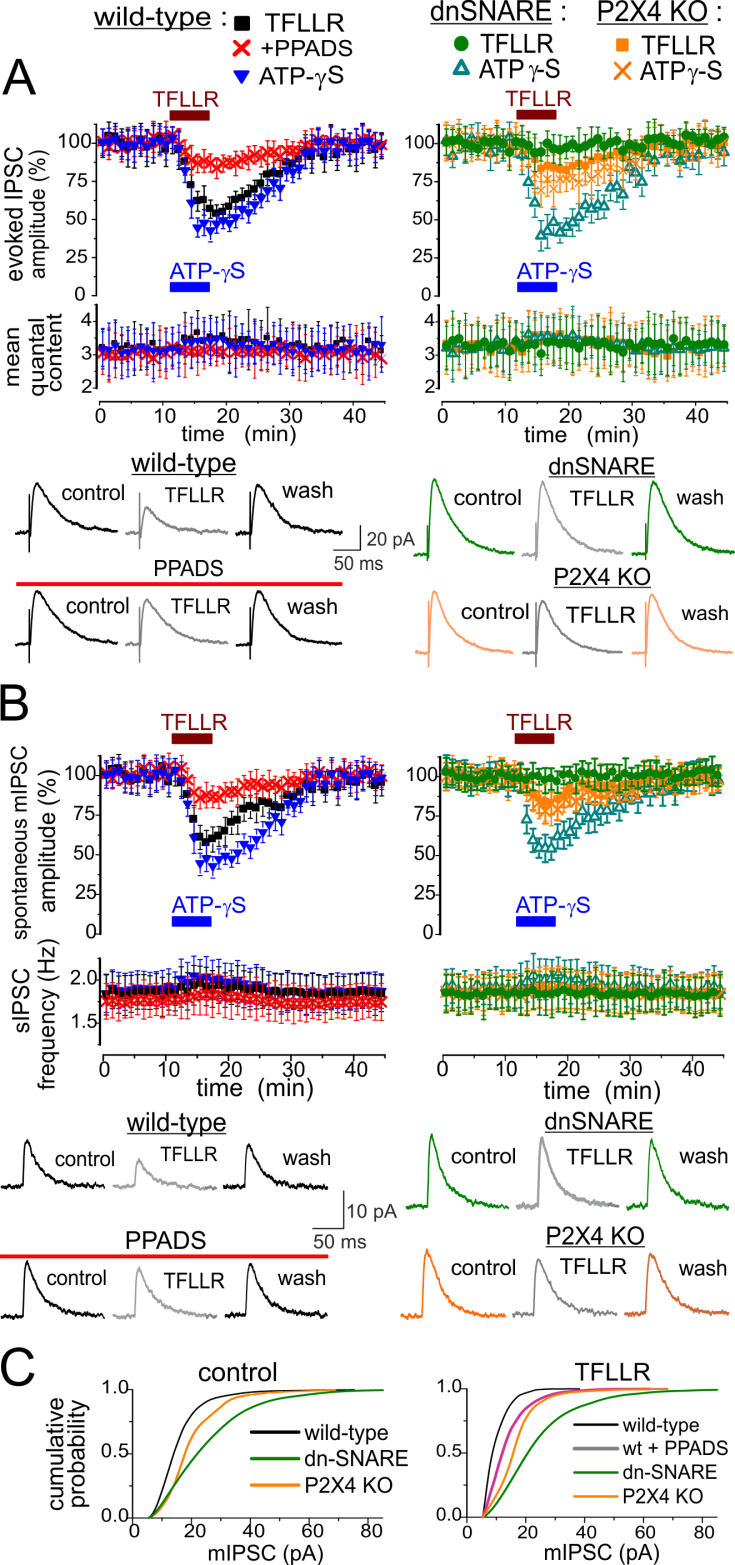
Exocytosis of ATP from astrocytes leads to down-regulation of inhibitory synaptic currents in neocortex. Astroglial exocytosis was elicited by application of PAR-1 receptor agonist as shown in Figures 2 and 3. (A) Plots show the time course of evoked IPSCs in the layer 2/3 pyramidal neurons of wild-type mice (left column) and dn-SNARE and P2X4 knockout mice (right column) during application of TFLLR (10 µM) and nonhydrolysable ATP-analog ATP-γS (10 µM). In the wild-type mice, TFLLR was also applied in the presence of P2 purinoreceptors antagonist PPADS (10 µM). Data points represent the mean amplitude and quantal content for six consecutive IPSCs evoked at −40 mV in the presence of CNQX (50 µM), D-APV (30 µM), and DPCPX (3 µM). Data show mean ± SD for the number of neurons as follows: 9 (WT, control), 7 (WT+PPADS), 6 (WT+ATP-γS), 8 (dn-SNARE), and 12 (P2X4-KO). The average waveforms (25 IPSCs) are shown below as indicated. (B) Time course of the amplitude of spontaneous mIPSCs recorded in the same conditions as above +1 µM TTX. Each point represents the average amplitude for a 1 min time window. Data points represent the mean amplitude and frequency of mIPSCs recorded with a 1 min time window. Data show mean ± SD for number of neurons as follows: 12 (WT, control), 5 (WT+PPADS), 5 (WT+ATP-γS), 8 (dn-SNARE), and 12 (P2X4-KO). The average waveforms (25 mIPSCs) are shown below. Note that the reduction in the inhibitory currents was strongly attenuated by the antagonist of ATP receptors and knocking out P2X4 receptors and there was no marked reduction in the IPSCs in the dn-SNARE mice. (C) Cumulative amplitude histograms of spontaneous mIPSCs (pooled for number of neurons indicated above) recorded in the baseline conditions (left) and after application of PAR-1 agonist (right). Histograms show the significant reduction in the mIPSCs amplitude caused by TFLLR. Amplitude histogram of mIPSCs recorded in dn-SNARE mice in control shows the marked right shift, indicating the up-regulation of baseline inhibitory transmission.

To test this hypothesis, we recorded inhibitory synaptic currents (IPSCs) in neocortical pyramidal neurons at a membrane potential of −40 mV (Figure 6) in the presence of glutamate receptor antagonists CNQX (50 µM) and D-APV (30 µM). Under these conditions, we observed inward purinergic currents simultaneously with outward Cl^−^-currents mediated by GABAA receptors (Figure 6). Similar to our previous results [13], outward IPSCs were completely inhibited by bicuculline in all 12 cells tested (unpublished data). The burst of ATP-mediated currents, induced in cortical neurons by activation of astrocytic Ca^2+^ signaling via PAR1 receptors (as shown in Figure 3A), was accompanied by significant decrease in the amplitude of GABA-mediated synaptic currents (Figure 6A,B). Amplitudes of evoked IPSCs and spontaneous mIPSCs recorded in the wild-type mice were reduced after application of 10 µM TFLLR by 44.3%±6.1% (*n* = 7) and 39.4%±6.3% (*n* = 12) correspondingly. In the dn-SNARE mice, the inactivation of GABA receptor-mediated synaptic currents was greatly diminished (Figure 6A,B). Application of TFLLR reduced the amplitude of evoked and spontaneous IPSCs in the cortical neurons of dn-SNARE mice just by 6.4%±8.7% (*n* = 8) and 4.3%±6.6% (*n* = 8), respectively. The difference in the action of TFLLR on the IPSCs in the wild-type and dn-SNARE was statistically significant with *p*<0.005 for both evoked and spontaneous currents. These results confirm the importance of astroglial exocytosis for the observed inactivation of GABA receptors. It also indicates the lack of unspecific action of PAR-1 agonist on inhibitory synaptic transmission.

The effect of TFLLR on inhibitory synaptic currents was mimicked by application of nonhydrolysable ATP analog ATP-γS (10 µM) and was considerably reduced by P2 receptor antagonist PPADS (10 µM). In the P2X4 KO mice, activation of astrocytes decreased the amplitude of evoked and spontaneous IPSCs (Figure 6A,B) only by 14.3%±8.2% and 15.7%±9.5% (*n* = 12); the difference between P2X4 KO and wild-type mice was significant with *p*<0.005. These data strongly support the participation of neuronal ATP receptors in the astrocyte-driven modulation of IPSCs.

We found out that exocytosis of gliotransmitters also caused long-term homeostatic modulation of inhibitory synaptic transmission. We observed a marked difference in the amplitude distribution of the baseline (control) amplitude of postsynaptic inhibitory currents in the wild-type and dn-SNARE mice (Figure 6C). The amplitude of mIPSCs in the cortical neurons before the application of TFLLR was much higher in the dn-SNARE than in the WT mice. The average baseline amplitude of mIPSCs was 23.5±8.3 pA in the dn-SNARE mice (*n* = 8) and 14.9±6.9 in the WT mice. Application of TFLLR caused the leftward shift of amplitude distribution of mIPSCs only in the WT mice; this shift was significantly reduced by PPADS (Figure 6C). The considerable difference in the baseline amplitude of miniature inhibitory currents in the WT and dn-SNARE mice provides the first evidence that vesicular release of gliotransmitters may be involved in the long-term homeostatic regulation of inhibitory transmission in the neocortex.

**Figure 7:**
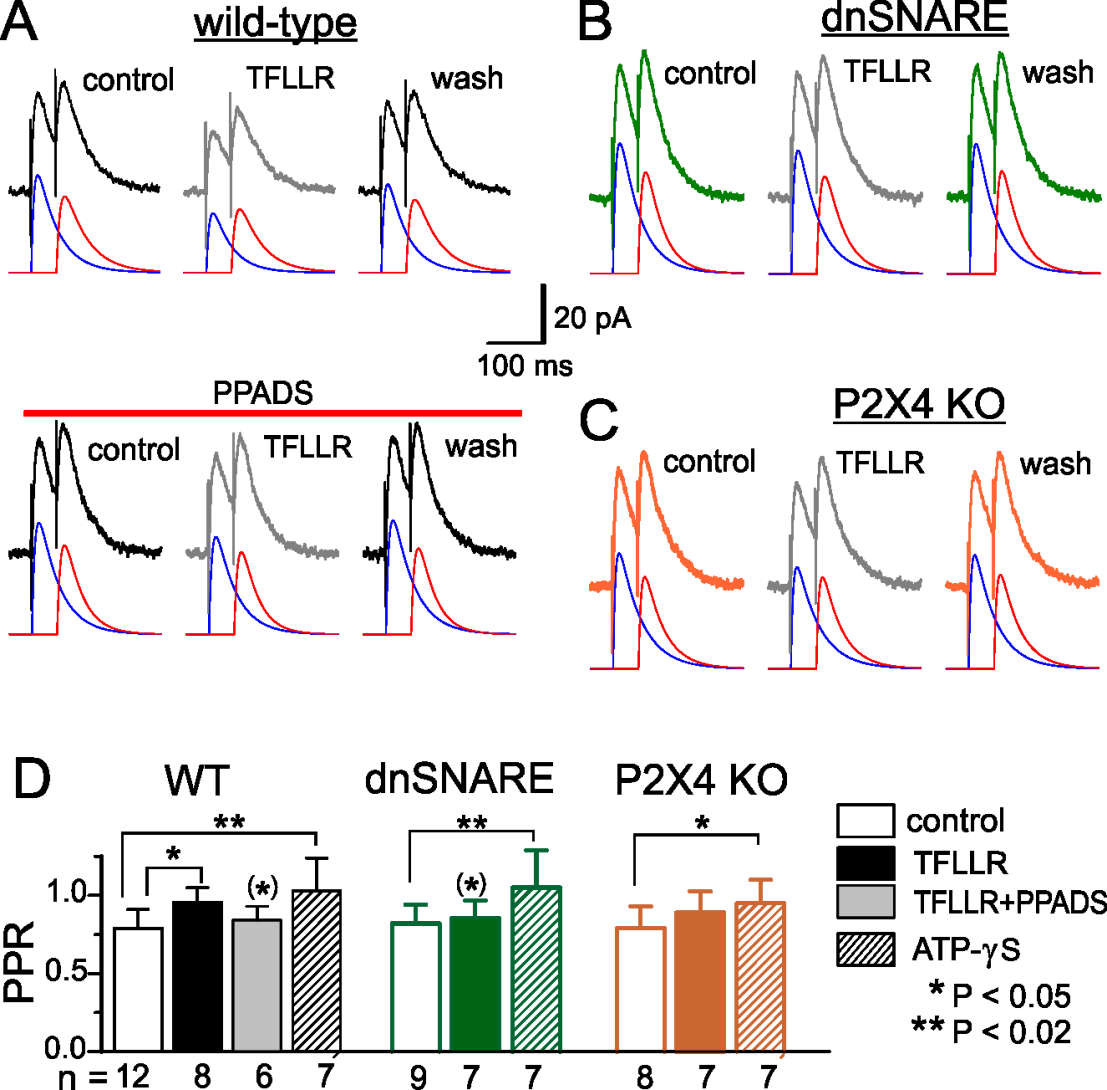
The presynaptic changes in the inhibitory synaptic currents in the neocortex evaluated by PPR. The IPSCs were recorded in the pyramidal neuron in the same conditions as described in Figure 6A. (A–C) Graphs show the representative IPSCs (average of 25 traces) evoked with a 50 ms interval in control and during application of 10 µM TFLLR. To calculate the PPR, the amplitude of IPSC was evaluated by best fit with two curves of mono-exponential rise and decay, depicted by red and blue lines. (D) The pooled data (mean ± SD) on PPR of IPCS measured before and during application of 10 µM TFLLR and 10 µM ATP-γS in the wild-type, dn-SNARE, and P2X4 knockout mice for number of cells as indicated. Asterisks * and ** indicate statistical significance of difference from the control of the same mice strain (two population *t* test), and asterisks in parentheses (*) indicate statistical significance of difference in the effect of TFLLR from the wild-type mice. Note the increase in PPR under action of TFLLR and ATP-γS, which contrasts with the decrease in the IPSCs amplitudes (Figure 6).

In order to elucidate the role of post- and presynaptic mechanisms in the modulation of inhibitory transmission, we evaluated the changes in the mean quantal content (Figure 6A), changes in the frequency of spontaneous mIPSCs (Figure 6B), and paired-pulse ratio (PPR) of evoked IPSCs (Figure 7). Neither the mean quantal content of IPSCs (Figure 6A) nor the mIPSCs frequency (Figure 6B) exhibited marked changes. However, we observed a moderate change in the PPR of IPSCs in the neocortical pyramidal neurons of wild-type mice (Figure 7A). The IPSCs evoked with a 50 ms interval in the control showed paired-pulse depression with mean PPR of 0.79±0.11 (*n* = 12), and application of TFLLR increased PPR by 0.16±0.07 (*n* = 8). The application of TFLLR did not cause a significant change in PPR in the dn-SNARE mice (Figure 7B,D), indicating the involvement of glial exocytosis in the mechanism. The effect of TFLLR was significantly reduced by PPADS (Figure 7A,D) and reproduced by application of ATP-γS both in the wild-type and dn-SNARE mice (Figure 7A,B,D). Hence, the presynaptic increase in the PPR of IPSCs was most likely mediated by ATP acting via P2 purinoreceptors. This result is consistent with previous reports of presynaptic facilitation of GABAergic synaptic transmission by ATP and P2 receptors [1],[2],[49].

There was no significant difference in the effects of TFLLR and ATP-γS between wild-type and P2X4 KO mice (Figure 7C,D). So the P2X4 receptors hardly make the large contribution in the increase of PPR, contrasting with their prominent role in the astrocyte-induced decrease of IPCS amplitude (Figure 6). One could speculate that presynaptic facilitation of IPSCs by glia-driven ATP can be mediated by other subtypes of P2X receptors and P2Y receptors, whose role in the presynaptic modulation in the brain was widely reported [1],[2].

More importantly, the above data clearly demonstrate that the large decrease in the amplitude of evoked and spontaneous IPSCs cannot be attributed to the presynaptic modulation of GABA release, and astrocyte-induced down-regulation of inhibitory transmission operates via postsynaptic mechanism.

The postsynaptic mechanism of IPSCs inactivation was corroborated by experiments where the Ca^2+^-dependent phosphorylation of GABA receptors was impaired by intracellular agents. First, we found that activation of P2X receptors in pyramidal neocortical neurons caused marked reduction of GABA-activated currents via Ca^2+^- and protein kinase C–dependent mechanism (Figure S11). Second, application of TFLLR to the cortical slices of wild-type mice did not cause marked reduction in the mIPSCs recorded in neurons perfused with intracellular Ca^2+^-chelator EGTA (10 µM) or protein kinase C antagonist GF109203X (30 nM). The mIPSC amplitude was reduced by just 2.5%±5.9% (*n* = 6) in the presence of intracellular EGTA and by 11.2%±5.5% (*n* = 4) in the presence of the protein kinase C antagonist (Figure S12). These results agree with previous reports on Ca^2+^- and PKC-dependent down-regulation of GABA receptors [47].

**Figure 8:**
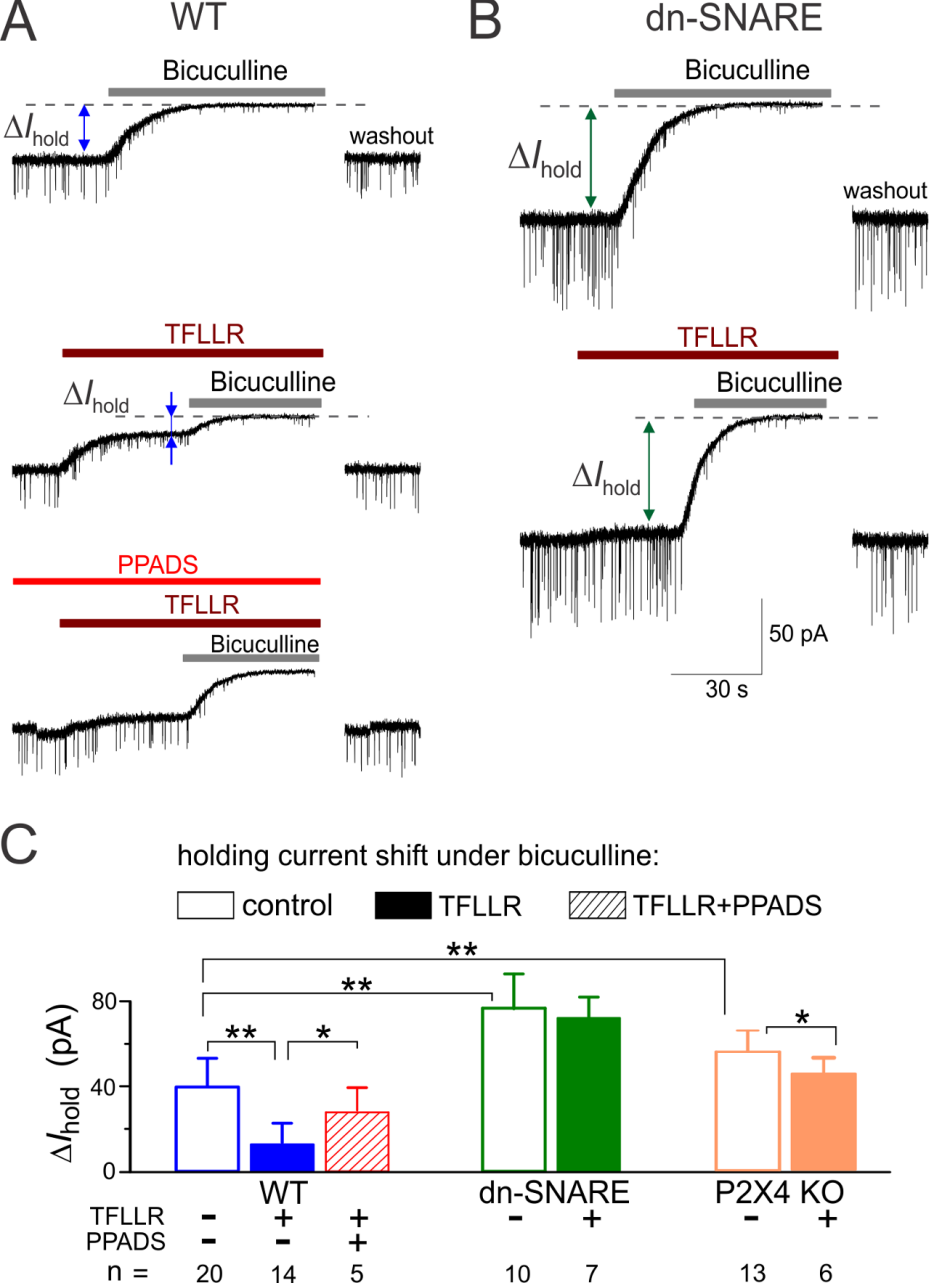
Exocytosis of ATP from astrocytes down-regulates the tonic inhibitory synaptic signaling. (A, B) Tonic GABA-mediated signaling was evaluated by upward shift in the whole-cell holding current (ΔI_hold_) caused by application of bicuculline (50 µM) to the layer 2/3 pyramidal neurons in brain slices of wild-type (A) and dn-SNARE (B) mice. Bicuculline was applied either in control (upper traces) or after activation of astrocytic signaling by 10 µM TFLLR (middle traces) or after application of TFLLR in the presence of ATP receptors antagonist PPADS (bottom trace). Currents were recorded in the presence of CNQX (50 µM) and D-APV (30 µM) at a holding potential of −80 mV. (C) The average amplitude of the tonic GABA-mediated current measured in different conditions in the wild-type, dn-SNARE, and P2X4 knockout mice for number of cells as indicated; statistical significance of difference in mean amplitude was as indicated (*) *p*<0.05 and (**) *p*<0.01. In the wild-type mice, activation of astroglial Ca^2+^ signaling significantly decreased the tonic GABA-mediate current; reduction in tonic current was attenuated by PPADS. In the dn-SNARE and P2X4 KO mice, tonic inhibitory current was significantly up-regulated, and activation of the astroglial Ca^2+^ had a much smaller effect on the tonic current than in the wild-type mice. These results suggest that exocytosis of ATP can lead to down-regulation of tonic inhibition in the neocortical neurons, most likely via P2X receptors.

In addition to the fast IPSCs (“phasic inhibition”), central neurons also receive a diffusional inhibitory signal mediated by the extrasynaptic GABA receptors continuously activated by small concentrations of GABA present in the extrasynaptic space (“tonic inhibition”) [50],[51]. Extrasynaptic GABA receptors, responsible for tonic inhibition, have been reported to undergo Ca^2+^-dependent phosphorylation [47],[48]. Thus, one could expect the impact of astrocyte-driven ATP not only on phasic but also on tonic inhibitory signaling in the neocortical pyramidal neurons. To verify this hypothesis, we used a conventional experimental paradigm where tonic inhibition is assessed by the change in the whole-cell holding current under action of GABA receptor blockers [50], in our case 50 µM bicuculline. In the wild-type mice (Figure 8A, top), layer 2/3 neurons showed the tonic current of 39.9±8.3 pA (*n* = 20) at membrane potential of −80 mV. The tonic current in the pyramidal neurons dn-SNARE mice was almost two times higher than in the wild-type (Figure 8B, top), reaching 76.9±15.1 pA (*n* = 10). A significant difference in the amplitude of tonic current between wild-type and dn-SNARE mice suggests that exocytosis of gliotransmitters from cortical astrocytes can modulate the activity of extrasynaptic GABA receptors in the adjacent neurons. Consistent with this notion, activation of gliotransmitter release by TFLLR caused a marked upward shift in the holding current in the pyramidal neurons of wild-type mice (Figure 8A, middle), and the rest of holding current was efficiently diminished by bicuculline. Application of TFLLR did not have a notable effect on tonic current in the dn-SNARE mice (Figure 8B, middle). The amplitude of tonic current recorded under action of TFLLR in the wild-type, dn-SNARE, and P2X4 KO mice was correspondingly 12.6±6.8 pA (*n* = 14), 72.2±9.1 pA (*n* = 7), and 43.4±7.2 pA (*n* = 6). The relative decrease in the amplitude of tonic current caused by TFLLR was, respectively, 68%±14%, 6%±8%, and 25%±11%. The down-regulation of tonic current by PAR-1 agonist was considerably attenuated by antagonist of ATP receptors (Figure 8A, bottom). The effects of TFLLR and PPADS in the wild-type mice as well as a difference between mice strains were statistically significant as indicated in Figure 7C. It has been recently shown that Ca-dependent modulation of astrocytic GABA GAT3 transporters in the hippocampus can alter an extracellular GABA level elevating the tonic current and decreasing the mIPSCs due to GABA receptor desensitization [52]. One should note that this pathway did not contribute significantly to PAR-1 agonist-induced modulation of inhibitory currents in neocortical neurons, since we observed a decrease in both phasic (Figure 6) and tonic currents (Figure 7). This notion was corroborated by our finding that blocking of the astroglial GAT3 GABA transporter increased the tonic current and decreased the amplitude of mIPSCs both in the wild-type and dn-SNARE mice (Figure S13). These data also show that dn-SNARE expression does significantly alter the activity of GABA transporters in cortical astrocytes.

Taken together, our experiments in brain slices demonstrate that exocytosis of ATP from cortical astrocytes *in situ* can activate the ATP receptors in the adjacent pyramidal neurons, leading to down-regulation of synaptic and extrasynaptic GABA receptors.

## Discussion

Detection of ATP released from acutely isolated cortical astrocytes using sniffer cells demonstrated the SNARE protein-dependent exocytosis of ATP from cortical (Figures 1 and 2) and hippocampal (Figure S2) astrocytes. Our experiments *in situ* show that release of ATP from astrocytes can be sensed by neighboring neurons where glia-derived ATP can activate neuronal purinoreceptors. Quantal behavior of astrocyte-induced phasic purinergic currents (Figures 3, 4, and S6) and their elimination in the bafilomycin-treated cortical slices (Table S2) confirm that they originated from the vesicular release of ATP. Combined with previous observation of astroglial purinergic modulation of neuronal activity in the hippocampus, cortex, and basal forebrain [12],[20], these data strongly support the universal character of a vesicular mechanism of ATP release as a gliotransmitter.

In the sniffer cell experiment we used P2X2 receptors as a detector for ATP. They have a moderate affinity for ATP (EC_50_ around 10 µM) that allowed us to avoid saturation and resolve the quantal behavior of astrocyte-evoked purinergic currents with a mean quantal size of about 8 pA (Figure 2D). To activate such response, the concentration of ATP released from astrocytes should have reached at least the low micromolar range. Detailed calculations, performed using the approach applied for extrasynaptic release and diffusion of glutamate [16],[53],[54], show that such concentration can be reached after release of about 1,000 molecules from a single glial synaptic-like vesicle, and the size of the “active spot” due to diffusion of ATP can reach as far as 1–2 µM (Figures S14A and S15). This estimation is in line with our data obtained using ATP microelectrode biosensors (Figure S10) as well as with previous evidence that the extracellular ATP level in the brain can reach 1–100 µM depending on the physiological and pathological context [1],[7],[55].

Similar calculations, as above, argue against involvement of lysosomal release in generation of purinergic currents observed in our experiments. Indeed, the typical lysosome has a diameter of about 300 nm and can contain more than 1,000,000 molecules of gliotransmitter [16]—that is, 1,000 times more than in the synaptic-like vesicle. Even though lysosome undergoes kiss-and-run exocytosis releasing only 10% of its content [16], one could expect the peak of a sniffer cell response to reach at least 200–500 pA, with most receptors on its surface saturated with agonist. So the observed quantal size of sniffer cell response (about 8 pA) is in much better agreement with release of ATP from synaptic-like vesicles.

The low micromolar level of ATP concentration can be sufficient for activation of P2X purinoreceptors abundantly expressed in the central neurons [1],[42],[44]. The data of electron microscopy and single molecular imaging [39],[56],[57] show both synaptic (mostly at the periphery of synaptic density) and extrasynaptic location of P2X receptors. Interestingly, P2X2 and P2X4 receptors are generally located peri-synaptically (i.e., at the edges of postsynaptic density) [56], which makes them accessible by ATP released from both nerve terminals and extrasynaptic glial release sites. This is consistent with our observation of strong sensitivity of astrocyte-driven sIPSCs to deletion of P2X4 receptors and inhibition of P2X2 receptors (Figures 3, 4, and S7). The distance to the source of ATP would affect the kinetics and amplitude of input mediated by peri-synaptic P2X receptors. Due to diffusion and rapid conversion to ADP, the transient of ATP concentration reaching P2X receptor after vesicular release from the distal (astroglial) site will have less magnitude but will decay longer than ATP transient after release from a close intrasynaptic site (as illustrated in Figure S15). This would lead to smaller quantal amplitude and slower kinetics of purinergic current of the glial origin as compared to neuronally activated synaptic currents. So the diversity in location of the ATP source could be the most plausible explanation for the difference in the amplitude and kinetics of purinergic sEPSCs of neuronal and glial origin observed in the neocortical neurons (Figures 3 and 4). An alternative explanation might be that EPSCs of smaller amplitude and slow kinetics were generated at neuronal synapses at a much longer electrotonic distance. In this case, however, the attenuation factor would increase gradually with distance, leading to smooth single-peaked distributions of EPSC amplitude and decay time due to the presence of large “intermediate” EPSCs. This contrasts with our observation of two peaks in the amplitude and decay time histograms (Figures 3–5). These arguments are supported by results of computer simulation using the model of neurotransmitter spillover [53],[54] adapted for release of ATP from glial and synaptic sites (Figures S14 and S15). Importantly, the key experimental evidence of the astroglial origin of purinergic currents of slower kinetics and smaller quantal size has been provided by their selective inhibition by disruption of vesicular ATP transport in astrocytes (Figure 5).

Although the possibility of nonvesicular gliotransmitter release from astrocytes has been previously reported in several brain regions [4],[7],[15],[58], our results do not show a strong contribution of nonvesicular pathways to the release of ATP in the neocortex. On a contrary, both sniffer cell and biosensor data suggest that vesicular mechanism brings major contribution to activity-dependent ATP release. One should note that incomplete inhibition of ATP release in the biosensor experiments could be explained by incomplete inhibitory action of bafilomycin treatment and dn-SNARE expression on vesicular release rather than notable contribution of nonvesicular mechanisms. It is possible that vesicular and nonvesicular ATP release operates in the neocortex in the different time scale, similarly to spinal cord astrocytes [15], where the fast initial exocytosis of ATP can be followed by slow secondary release of ATP through the pannexin and connexin hemichannels [15]. So it is plausible that vesicular and nonvesicular mechanisms of ATP release coexists in the neocortex, but nonvesicular release was not activated in our experimental context.

It is traditionally considered that physiological astroglial Ca^2+^ signaling is driven mainly by InsP_3_-mediated release from intracellular stores [16]. Calcium signals arising from activation of metabotropic receptors are believed to be primarily responsible for control of exocytotic release of gliotransmitters [9],[16],[17]. Although the role of InsP_3_-induced Ca^2+^ signaling in astroglial physiology was questioned [27], more recent data strongly support the importance of this pathway for glia-derived modulation of synaptic transmission in the hippocampus [29],[45] and neocortex [28],[30]. We have shown previously that ionotropic NMDA and P2X receptors can mediate a significant fraction of the synaptically driven Ca^2+^ rise in cortical astrocytes [5],[6],[59]. Our present data suggest that astrocytic NMDA receptors can trigger the release of ATP, acting in parallel to the metabotropic receptors (Figure 2). The capability of ionotropic receptors to control of exocytosis of gliotransmitters (Figure 2) might account for a lesser than expected impact of modulation of astroglial InsP_3_ signaling on synaptic transmission and plasticity.

Our results shown in Figures 1–4 provide a strong evidence of ATP release by exocytosis and support previous observations of presence of vesicular ATP transporters and synaptic-like vesicles in astrocytes [22],[24],[26]. Observation of vesicular location of VNUT1 (Figures S5 and S6), even with inherent limitations of immunostaining procedure (see Text S1), agrees with previous reports [22],[24],[26] and with our functional data as well. Co-localization of VNUT1 and synaptic vesicle markers (Figure S5), rather small quantal size and fast kinetics of glia-driven purinergic currents (Figures 1–4), suggest that release of ATP from synaptic-like vesicles can make a significant contribution into purinergic gliotransmission in cortical astrocytes.

Until recently, the action of ATP as gliotransmitter was associated mainly with presynaptic adenosine receptors [4],[12],[20]. It was also shown that astrocyte-derived ATP could operate through P2X7 receptors to enhance presynaptic release of glutamate in hypothalamic neurons [10]. Although we observed a moderate presynaptic modulation of inhibitory transmission (Figure 7), our main finding is that glia-driven ATP can directly activate excitatory currents in neurons acting via postsynaptic P2X receptors (Figures 3–5). We showed that activation of neuronal purinoreceptors by astrocyte-driven ATP caused a dramatic change in the inhibitory transmission in neocortex inhibiting postsynaptic and extrasynaptic GABAA receptors (Figures 6 and 7). ATP receptors can act through the Ca^2+^-dependent phosphorylation by protein kinase C (Figures S11 and S12); the latter mechanism is intrinsic for GABA receptors [43],[60].

The physiological relevance of exocytosis of gliotransmitters was strongly supported by our finding of significant increase in the baseline phasic and tonic inhibitory signaling in the cortical neurons of dn-SNARE mice (Figures 6 and 7). This result suggests that release of gliotransmitters, presumably ATP, can be involved in long-term homeostatic regulation of neuronal GABA receptors by a mechanism that has yet to be investigated. Our evidence of down-regulation of inhibitory synaptic transmission in the neocortex goes in line with observations that enhanced Ca^2+^ signaling in cortical astrocytes contributed to neuronal excitotoxicity and epilepsy [18],[61].

Our results outline a novel mechanism of action of ATP as a gliotransmitter: in addition to catabolism of ATP to ADP and adenosine and modulation of synaptic transmission via presynaptic purinoreceptors [12], ATP can enhance neuronal excitability by down-regulating the phasic and tonic inhibition acting via postsynaptic P2 receptors. Our present (Figures 6–8) and previous [11] results show that modulation of postsynaptic receptors activated by Ca^2+^ influx via purinergic P2X receptors can provide an efficient mechanism of regulation of signaling within tripartite synapse. Data on a large contribution of P2X4 receptors to glia-driven modulation of neuronal signaling (Figures 6–8) go in line with a previous observation of a facilitatory role for astroglial release of ATP [12] and P2X4 receptors [43] in the long-term potentiation of synaptic transmission in the hippocampus.

Our findings give further insight into the role of P2X receptors in the CNS. There is growing consensus that, despite a clear evidence of participation of P2X receptors in the excitatory synaptic transmission in several brain areas, they bring notable contribution to slow neuromodulation rather than fast excitation [44]. The above results suggest that P2X receptors can also mediate glia-to-neuron signals, which can be activated in the millisecond time scale and have more long-lasting consequences for neuronal excitability.

Hippocampal astrocytes have been recently reported to decrease the amplitude of mIPSCs via Ca^2+^-dependent modulation of GABA transport [52] and increase the frequency of mIPSCs via P2Y receptors of inhibitory interneurons, presumably activated by slow release of ATP from astrocytes through connexin hemichannels [62].

Our data suggest that presynaptic ATP receptors can enhance GABA release in the neocortex (Figure 7). Down-regulation of both phasic and tonic postsynaptic GABA receptors by astrocyte-driven ATP (Figures 7 and 8) can act downstream of these pathways and significantly affect their impact on inhibitory transmission. Interplay between post- and presynaptic pathways of glial modulation could have diverse effects on neuronal excitability in different physiological contexts. Apparently, the down-regulation of inhibitory transmission provided by postsynaptic P2X receptors prevails in the neocortical pyramidal neurons, at least in our experimental conditions (Figures 6 and 7).

It becomes evident now that even release of one gliotransmitter, ATP (followed by formation of ADP and adenosine), can activate a variety of pre- and postsynaptic regulatory cascades that can affect synaptic efficacy in opposite ways. Combined with diversity of vesicular and nonvesicular pathways of ATP release and the possibility of release of other gliotransmitters, such as glutamate and D-serine [9],[33],[63], this may confer more complex behavior to tripartite synapse than previously assumed [16],[27].

In summary, our data suggest that Ca^2+^ elevation in cortical astrocytes, which might occur in response to signals from neurons and/or propagation of glial Ca^2+^ waves, can trigger exocytosis of ATP from synaptic-like vesicles and activate neuronal P2X receptors that are located at the periphery of synapse. Ca^2+^ signaling via neuronal ATP receptors can cause phosphorylation-dependent inhibition of postsynaptic GABA receptors acting downstream of astroglial modulation of presynaptic GABA release and GABA uptake. Our results strongly support physiological importance of exocytosis of gliotransmitters, in particular ATP, in communication between astrocytes and neurons and modulation of synaptic efficacy.

## Materials and Methods

Experiments were performed on astrocytes and neurons in the somato-sensory cortex of dn-SNARE transgenic mice [12],[20] and their wild-type (WT) littermates; in some experiments, the P2X4 receptor knockout mice [43] were used. Genotypes of animals were verified by PCR from ear samples. Initial experiments to investigate release of ATP from astrocytes were also performed in transgenic mice expressing enhanced green fluorescent protein (EGFP) under the control of the glial fibrillary acidic protein (GFAP) promoter [34],[59],[64]. Data obtained in the experiments on GFAP–EGFP mice (*n* = 5–6 for each type of experiments) did not differ significantly from data obtained in the WT mice of the same age. For clarity, all data referred to here as wild-type are reported solely for wild-type littermates of dn-SNARE mice; usage of GFAP–EGFP mice was explicitly stated where appropriate.

### Slice and Cell Preparation

Mice (8–12 wk and 9 mo old) were anaesthetized by halothane and then decapitated, in accordance with UK legislation. Brains were removed rapidly after decapitation and placed into ice-cold physiological saline containing (mM) NaCl 130, KCl 3, CaCl_2_ 0.5, MgCl_2_ 2.5, NaH_2_PO_4_ 1, NaHCO_3_ 25, glucose 15, pH of 7.4 gassed with 95% O_2_ to 5% CO_2_. Transverse slices (280–300 µm) were cut at 4°C and then placed in physiological saline containing (mM) NaCl 130, KCl 3, CaCl_2_ 2.5, MgCl_2_ 1, NaH_2_PO_4_ 1, NaHCO_3_ 22, glucose 15, pH of 7.4, and kept for 1–4 h prior to cell isolation and recording.

Astrocytes were acutely isolated using the modified “vibrating ball” technique [34],[59]. The glass ball (200 µm diameter) was moved slowly some 10–50 µm above the slice surface, while vibrating at 100 Hz (lateral displacements 20–30 µm). Isolation protocol was adjusted to provide a high yield of astroglial cells. This technique preserves the function of membrane proteins and therefore is devoid of many artifacts of enzymatic cell isolation and culturing procedures. In particular, vibro-dissociated astrocytes retain many morphological features (e.g., GFAP–EGFP fluorescence, size, proximal processes) and functional properties (e.g., high potassium conductance, glutamate transporters, Ca^2+^ signaling) while being completely isolated from neuronal somata and nerve terminals. The composition of external solution for all isolated cell experiments was (mM) 135 NaCl, 2.7 KCl, 2.5 CaCl_2_, 1 MgCl_2_, 10 HEPES, 1 NaH_2_PO_4_, 15 glucose, pH adjusted with NaOH to 7.3. Astrocytes were identified by their morphology under DIC observation, EGFP fluorescence (astrocytes from dn-SNARE and GFAP-EGFP mice), and functional properties as described previously (see also Table S1) [34],[59].

In the experiments with dn-SNARE mice, administration of Dox [12],[20] has been removed 4 wk prior to electrophysiological studies. According to our observations, about 70% of astrocytes in the layer 2/3 of somatosensory cortex *in situ* and freshly isolated cortical astrocytes exhibited EGFP fluorescence. The astrocytic identity of EGFP reporter and dn-SNARE-transgene-expressing cells has previously been confirmed for the dn-SNARE line [12],[20]. So one can expect that at least 60%–65% of astrocytes in the somatosensory cortex of dn-SNARE mice express the dn-SNARE domain of synaptobrevin II [20]. In the experiments with freshly isolated astrocytes, only the fluorescent cells have been selected to ensure the impairment of SNARE complex function. In the experiments in the somatosensory cortex of dn-SNARE mice *in situ*, electrophysiological recordings have been performed in the areas with higher density of EGFP-expressing astrocytes to maximize the putative impact of the loss of SNARE function on neighboring neurons. To increase the probability of a neuron lying within a functional island of synapses [46] controlled by dn-SNARE astrocytes, we recorded from the neurons located in the close proximity to at least two fluorescent cells, as illustrated in Figure S16.

### Detection of ATP Release of Astrocytes Using Sniffer Cells

After isolation from a brain slice, cortical astrocytes were incubated with 5 µM Ca^2+^ indicator Fluo4-AM (or Rhod-2 AM) and 10 µM of photoliable Ca^2+^ chelator *o*-nitrophenyl-EGTA-AM (NP-EGTA) for 30 min, re-suspended in a small volume (200–300 µl) of fresh extracellular medium, and placed over cultured HEK293 cells expressing GFP-tagged P2X2 receptors to ATP (HEK293-P2X2, gift from Prof. R. Evans, University of Leicester, UK). To evaluate release of ATP, the transmembrane currents were recorded in the HEK293-P2X2 cells that had an astrocyte lying on their surface; simultaneously, elevation in cytosolic Ca^2+^ concentration was induced in the astrocytes by UV uncaging.

It has to be noted that contrary to the experiments with astrocytes in culture [21], the spatial density of acutely isolated cells in our experiments was rather low, as shown previously [34]. So we could easily select HEK cells contacting astrocytes with no other cell lying in the immediate vicinity (e.g., as shown in Figures 1 and S2). We performed recordings from the HEK293 cell–astrocyte couples, which were separated from any other cells by at least 15–20 microns of free space to ensure that HEK293-P2X2 cells were activated by ATP released only from contacting astrocyte.

Flash photolysis and fluorescent imaging were performed with the aid of a IX51 inverted microscope and epifluorescent illumination via UPLSAPO60XW/NA1.2 objective (Olympus, Tokyo, Japan). To monitor the intracellular Ca^2+^ level, astrocytes were constantly illuminated at 480±10 nm using OptoLED light source (Cairn Research, Faversham, UK), and fluorescence was measured at 535±25 nm. Astrocytes from dn-SNARE and GFAP-EGFP mice were loaded with Ca^2+^ indicator Rhod-2 and illuminated at 530±10 nm; fluorescence was measured at 590±30 nm. The fluorescent images were recorded using Retiga 2000R enhanced CCD camera (*QImaging*, Canada); exposure time was 35 ms at 2X2 binning. Elevation in the intracellular Ca^2+^ level was evaluated by a ΔF/F_0_ ratio averaged over the whole cell image after background subtraction.

For uncaging of intracellular Ca^2+^, cells were illuminated by a brief pulse (200 ms) of UV light (365±10 nm) emitted by high-power LED NCSU033AT (Nichia, Tokyo, Japan), peak power 500 mW, and estimated power at an objective >150 mW. Illumination was delivered via OptoLED dual-port epifluorescence condenser (Cairn Research, UK). In addition to photolysis, Ca^2+^ rise was induced in the astrocytes by fast application of agonists of PAR-1 receptors (10 µM TFLLR) or NMDA receptors (20 µM NMDA); these agonists did not cause any response in HEK293-P2X2 cells when applied without astrocytes placed over HEK cells.

### Electrophysiological Recordings

Whole-cell voltage clamp recordings from HEK293-P2X2 cells, neurons, and astrocytes were made with patch pipettes (4–5 MΩ for neurons and HEK293-P2X2 cells and 6–8 MΩ for astrocytes) filled with intracellular solution (in mM): 110 KCl, 10 NaCl, 10 HEPES, 5 MgATP, pH 7.35. Intracellular solution for recording in neurons and astrocytes contained 0.2 mM EGTA, and the solution for HEK293-P2X2 cells contained 10 mM EGTA and 1 mM CaCl_2_. In some experiments (simultaneous recording of GABA-mediated and ATP-mediated synaptic currents), KCl was replaced by KGluconate. Currents were monitored using an AxoPatch200B patch-clamp amplifier (Axon Instruments, USA) filtered at 2 kHz and digitized at 4 kHz. Experiments were controlled by the PCI-6229 data acquisition board (NI, USA) and WinFluor software (Strathclyde University, UK); data were analyzed by self-designed software. Liquid junction potentials were compensated with the patch-clamp amplifier. Series and input resistances were, respectively, 5–7 MΩ and 500–1100 MΩ in the HEK cells and neurons and 8–12 MΩ and 50–150 MΩ; both series and input resistance varied by less than 20% in the cells accepted for analysis. For activation of synaptic inputs, axons originating from layer IV–VI neurons were stimulated with a bipolar coaxial electrode (WPI, USA) placed in the layer V close to the layer IV border, approximately opposite the site of recording; the stimulus duration was 300 µs. For the recording of EPSCs and IPSCs, the stimulus magnitude was set 3–4 times higher than minimal stimulus adjusted to activate the single-axon response in the layer II pyramidal neurons as described in [13]. In order to trigger synaptically driven astroglial Ca^2+^ transients, the single episode of theta-burst stimulation (HFS) was delivered; the HFS episode consisted of 5 pulses of 100 Hz stimulation, repeated 2 times with 200 ms interval (total 10 pulses per episode).

### Fluorescent Ca^2+^ Imaging in Astrocytes Situ

To monitor the cytoplasmic-free Ca^2+^concentraton [Ca^2+^]_i_, cortical astrocytes were loaded by 40 min incubation with Fura-2AM. For fura-2 excitation, cells were alternately illuminated at wavelengths of 340±5 nm and 380±5 nm using the OptoScan monochromator (Cairn, Faversham, UK). Fluorescent images were recorded using Olympus BX51 microscope equipped with UMPLFL20x/NA0.95 objective and 2× intermediate magnification and Andor iXon885 EMCCD camera; exposure time was 35 ms at 2×2 binning; experiments were controlled by WinFluor software. The [Ca^2+^]_i_ levels were expressed as F_340_/F_380_ ratio averaged over the whole-cell image.

To investigate the Ca^2+^ signaling activated by PAR-1 receptor agonist, cortical neurons and astrocytes of wild-type and dn-SNARE mice were loaded with 50 µM Fluo-4. The whole-cell voltage-clamp recordings were used to confirm the identification of neurons and astrocytes and verify the lack of changes in the basic electrophysiological properties of the dn-SNARE astrocytes. The Fluo-4 fluorescence signal was excited at 488±10 nm and measured at 530±20 nm; the fluorescent images were recorded and analyzed as described above.

### Multiphoton Fluorescent Microscopy in Astrocytes

In parts of the experiments (Figure 1, Figure 5, Figure S5, Figure S16), two-photon imaging of neurons and astrocytes was performed using Zeiss LSM-7MP multiphoton microscope coupled with the SpectraPhysics MaiTai pulsing laser; experiments were controlled by ZEN LSM software (Carl Zeiss, Germany). Images were further analyzed offline using ZEN LSM (Carl Zeiss) and ImageJ (NIH) software.

For investigation of vesicular dynamics (Figure 1C), acutely isolated cortical astrocytes were loaded with FM1-43 fluorescent dye (2.5 µM), and FM1-43 fluorescence was excited at 820 nm and observed at 560±20 nm.

For immunolabeling of vesicular transporters, secretory organelles, and astroglial markers, acutely isolated astrocytes were incubated with 0.1 µg/ml of antibodies following antibodies: rabbit polyclonal anti-VNUT1 (T-12), goat polyclonal anti-cathepsin D (N-19, Santa Cruz Biotechnology), mouse monoclonal anti-VGLUT1 (McKA1), mouse monoclonal anti-PSD95 (6G6-1C9), mouse monoclonal anti-GLT-1 (10B7, Abcam), mouse monoclonal anti-NeuN (A60), rabbit monoclonal anti-S100b (EP1576Y, Millipore), goat monoclonal anti-SV2A, mouse monoclonal anti-NG2, and rabbit polyclonal anti-GFAP (Sigma). Prior to cell loading, antibodies were conjugated to the green fluorescent dye DyLight488 (VNUT1, VGLUT1, PSD-95, NeuN, NG2, and GFAP) or red fluorescent dye DyLight594 (VNUT1, SV2A, cathepsin-D, LAMP3, GLT-1, S100β) using Lighting-Link antibody conjugation system (Innova Bioscience, Cambridge, UK) according to the manufacturer's protocol. Antibodies to VNUT1 (recognizing intraluminal epitopes), VGLUT1 (recognizing cytoplasmic epitope), and cathepsin-D (intraluminal epitope) were applied to living astrocytes directly; other antibodies were conjugated with BioPORTER protein delivery reagent (Genlantis, San Diego, CA) 10 min prior to incubation. Immediately after isolation from the neocortical slice, living astrocytes were pre-incubated with 2% of normal bovine serum (Sigma) in the extracellular saline for 30 min to block unspecific binding sites. After them, cells were gently washed two times with clean extracellular saline for 5 min and then incubated with antibodies for 60 min at room temperature. After incubation, cells were washed with laminar flow of extracellular solution in the microscope recording chamber for 30 min prior to image recording.

Fluorescence was excited at 820 nm, GFAP-EGFP and DyLight488 signal was observed at 520±10 nm, and DyLight594 signal was observed at 590±20 nm. The photomultiplier gain of the two-photon microscope was adjusted to avoid saturation in both channels but in the same time did not differ more than 10% between red and green channel. Colocalization analysis of images was carried out using ImageJ software and methods described in [65].

### Measurement of Extracellular ATP Concentration in Brain Tissue

The concentration of ATP within cortical slices (Figure S10) was measured using microelectrode biosensors obtained from Sarissa Biomedical Ltd (Coventry, UK). A detailed description of the properties of ATP biosensors and recording procedure has been published previously in [7],[55],[58]. Briefly, biosensors consisted of ATP metabolizing enzymes immobilized within a matrix on thin (25–50 µM) Pt/Ir wire. This allowed insertion of the sensors into the cortical slice and minimized the influence of a layer of dead surface tissue. The concentration of ATP has been calculated from the difference in the signals of two sensors: a screened ATP sensor and screened null-sensor, possessing the matrix but no enzymes. This allowed us to compensate for release of any nonspecific electro-active interferents. Biosensors show a linear response to increasing concentration of ATP and have a rise time of less than 10 s [55]. Biosensors were calibrated with known concentrations (10 µM) of ATP before the slice was present in the perfusion chamber and after the slice had been removed. This allowed compensation of any reduction in sensitivity during the experiment. Biosensor signals were acquired at 1 kHz with a 1400 CED interface and analyzed using Spike 6.1 software (Cambridge Electronics Design, Cambridge, UK).

### Data Analysis

All data are presented as mean ± SD, and the statistical significance of difference between data groups was tested by two-population *t* test, unless indicated otherwise. The spontaneous transmembrane currents recorded in HEK293-P2X2 cells and neurons were analyzed off-line using methods described previously [13],[54]. Briefly, phasic transmembrane currents of an amplitude higher than 2 SD of baseline noise were selected for the initial detection of spontaneous events. Then every single spontaneous event was analyzed within the 140 ms time window, and its amplitude and kinetics were determined by fitting the model curve with single exponential rise and decay phases. As a rule, mean square error of fit amounted to 5%–20% of peak amplitude depending on the background noise. Whenever error of fit exceeded 25%, spontaneous currents were discarded from further analyses.

The amplitude distributions of spontaneous and evoked currents were analyzed with the aid of probability density functions and likelihood maximization techniques [54]; all histograms shown were calculated as probability density functions. The amplitude distributions were fitted with either multiquantal binomial model or bimodal function consisting of two Gaussians with variable peak location, width, and amplitude. The decay time distributions were fitted with bimodal functions. Parameters of models were fit using likelihood maximization routine. For each particular distribution, the fit with quantal or bimodal model was accepted only when it has a confidence level α≤0.05. To monitor and analyze the time course of changes in the amplitude and frequency of spontaneous currents, the amplitude and frequency were averaged over the 1 min time window.

For the basic analysis of the time course of quantal parameters of the evoked IPSCs, the mean quantal content was evaluated as reciprocal square of coefficient of variation [54], and the quantal size was calculated as ratio of mean amplitude to the mean quantal content.

### Ethics

All animal work has been carried out in accordance with UK legislation and "3R" strategy. Research has not involved non-human primates.

## Supporting Information

Data S1 **Raw data for Figure S5.**
(ZIP)

Data S2 **Raw data for Figure S6.**
(ZIP)

Figure S1 **Differential action of PAR-1 receptor agonist in the pyramidal neurons and astrocytes of neocortex.** The pyramidal neurons (A–C) and astrocytes (E–G) of somatosensory cortex layer 2/3 of wild-type and dn-SNARE mice were loaded with Ca^2+^ indicator Fluo-4 via patch-pipette. Ca^2+^-signals were evoked in neurons and astrocytes by a 30-s-long rapid bath application of 30 µM TFLLR and 100 µM L-Glutamate to cortical slices. The membrane holding potential during Ca^2+^ measurements was −40 mV in the neurons and −80 mV in the astrocytes. (A) The representative gradient-contrast image and (below) electrophysiological characterization of cortical pyramidal neurons of dn-SNARE mice; the high input resistance and fast voltage-gated Na^+^ current can be seen. (B) The representative Ca^2+^ transients evoked in the neuron of dn-SNARE mouse by application of TFLLR and glutamate; the panel (C) shows representative pseudocolor fluorescent images recorded at rest and at the peak of Ca^2+^ transients as indicated. Panel (D) shows the pooled data (mean ± SD for number of neurons indicated) of peak Ca^2+^ elevation; the difference between TFLLR and glutamate-evoked response was statistically significant with (*) *p* = 0.004 (one-way ANOVA). The PAR-1 receptors did not elicit the notable rise in the intracellular Ca^2+^ level in cortical pyramidal neurons of both wild-type and dn-SNARE mice. (E) The gradient contrast and EGFP fluorescence images and electrophysiological characterization of cortical astrocyte of dn-SNARE mouse; the low input resistance and large K^+^ current can be seen. Panels (F) and (G) show the elevation in the intracellular Ca^2+^ level evoked in the cortical astrocyte of dn-SNARE mouse by application of TFLLR and glutamate. Panel (H) shows the pooled data (mean ± SD for number of astrocytes indicated) of peak Ca^2+^ elevation; the was no significant difference between TFLLR-evoked response in the wild-type and dn-SNARE mice (one-way ANOVA). The PAR-1 receptors mediated substantial Ca^2+^ elevation in cortical astrocytes. These data also indicate the lack of adverse effect on expression of dn-SNARE protein on the basic functional properties of cortical astrocytes.
(TIF)

Figure S2 **Detection of ATP released from the hippocampal CA1 astrocytes with the aid of sniffer cells.** (A) The astrocytes acutely dissociated from the CA1 hippocampal area of the brain slice of EGFP-GFAP mouse [61] have been resuspended, loaded with UV-photoliable Ca^2+^-chelator NP-EGTA and Ca^2+^-indicator Rhod-2 AM, and placed over cultured HEK293 cells expressing P2X2 receptors. Lower panel shows functional characterization of astrocytes, using voltage-clamp recordings (performed after the uncaging experiment). From left to right, whole-cell currents activated by the series of depolarizing steps from a holding potential of −80 mV and currents evoked by fast application of 20 µM NMDA and 100 µM glutamate at −80 mV. The current evoked by glutamate in the presence of 30 µM NBQX and 50 µM D-AP5 is mediated by glutamate transporters. (B) Rhod-2 fluorescent signals have been monitored in the hippocampal astrocytes simultaneously with whole-cell recording of transmembrane current in HEK293 cells voltage-clamped at −80 mV in the control (upper panels) and in the presence of 10 µM PPADS (low panels). Flash of UV-light (365 nM) caused an elevation of cytosolic Ca^2+^ in the astrocyte followed by the burst of phasic currents in the HEK293 cell; a few spontaneous events could be observed in control in the absence of UV illumination. Similar burst of activity in the sniffer cell was induced by the specific agonist of astroglial PAR-1 receptors (TFLLR). Both the baseline and UV-elicited spurious currents were strongly inhibited (decrease in the amplitude was 89%±6% and 93%±5%, respectively; *n* = 4) after application of P2X receptor antagonist PPADS, confirming that they were mediated by receptors to ATP. (C) The amplitude and frequency of spurious currents measured in the HEK293 cell before (over 10 min time window) and after stimulation of astrocytes isolated from hippocampal CA1 region of GFAP-EGFP and dn-SNARE mice by UV-flash and application of 10 µM TFLLR and 20 µM NMDA (8 s time window). The data are presented as mean ± SD for number of cells as indicated; statistical significance of the difference between GFAP-EGFP and dn-SNARE astrocytes in the mean amplitude and frequency was as indicated by asterisks (*) *p* = 0.05 and (**) *p* = 0.005 (two-population *t* test). The significant decrease in the ATP release from CA1 astrocytes of dn-SNARE mice argues for a major role of exocytotic mechanism.
(TIF)

Figure S3 **Amplitude histograms for astrocyte-induced purinergic currents in the HEK293-P2X2.** Bar histograms show amplitude distributions built by conventional binning method for the same datasets as distributions shown in Figure 2D; bin size was 1 pA for currents recorded in the control and 0.5 pA for bafilomycin. Solid (color) lines correspond to distributions, depicted in Figure 2D, scaled by the corresponding sample size. Note that the distributions shown in Figures 2D are probability density functions, calculated using independent method as described in [51], rather than smoothed bin histograms. Probability density functions do not use amplitude binning. Good agreement between bin histograms and probability density functions verifies that quantal pattern of sniffer cell currents was not an artifact of analysis procedure.
(TIF)

Figure S4 **Lack of contribution of TREK-1 and Best1 channels to the release of ATP from cortical astrocytes.** Release of ATP from cortical astrocytes of wild-type mice was detected using the sniffer cells as described in Figures 1 and 2. Elevation of the cytosolic Ca^2+^ level was elicited in the astrocytes by rapid application of agonist PAR-1 metabotropic receptor TFLLR (10 µM) in control and in the presence of inhibitor of TREK-1 potassium channels fluoxetine (100 µM) and inhibitors of Ca^2+^-sensitive chloride channels DIDS (300 µM) and NPPB (100 µM). The latter inhibitors efficiently block best1 channels. (A, B) Representative astrocytic Ca^2+^ transients and recordings of transmembrane currents in HEK293-P2X2 cell voltage-clamped at −80 mV. (C) The diagrams show the mean amplitude and frequency of phasic currents in the sniffer cells (mean ± SD for 15 cells in the control and 5 cells for each inhibitor). Note the lack of effect of TREK-1 and Best1 channels blockers on astrocyte-driven purinergic currents in the sniffer cells.
(TIF)

Figure S5 **Colocalization of vesicular ATP transporters with exocytotic organelle markers.** Living, acutely isolated cortical astrocytes were labeled with antibodies to vesicular transporters (VNUT1 and VGLUT1) and synaptic vesicle (SV2A) and lysosomal (LAMP3 and Cathepsin-D) markers as described in Materials and Methods. Antibodies were conjugated to fluorescent dyes DyLight488 (green) and DyLight594 (red) prior to astrocyte labeling. To verify the identification of astrocytes and confirm the lack of unspecific immunostaining, cells were also immunolabeled with glial and neuronal markers. After image recordings, electrophysiological characterization of cells was performed as described in Materials and Methods and shown in the Figures 1, S1, and S2 and Table S1. (A–D) The representative two-photon fluorescence images (maximal intensity projections of Z-stack) and results of colocalization analysis, carried out using NIH ImageJ 1.43 software. The correlation between green and red fluorescence (images in the right column) is depicted as a product of the relative differences from the mean (PDM) for each pixel; the pseudocolor PDM images were generated as an output of ImageJ analysis routine. Positive values (bright yellow) are indicative for good co-localization of green and red signals, negative values (blue-violet) indicate segregation, and black color shows the lack of correlation. Note the different extent of scale for PDM values in (A–D). All scale bars in (A–D) are 5 µm. Raw images for panels (A–D) are uploaded as Data S1. (A) Images show good correlation between VNUT1 and SV2A markers. (B, C) Staining of cortical astrocytes for lysosomal markers and correlation between VNUT1 and LAMP3 or cathepsin-D was weaker than for synaptic vesicle marker. Note that VNUT1 was stained using antibodies recognizing the intraluminal epitope and therefore could very likely be labeled as a result of active endocytosis. (D) Images show examples of astrocyte immunostained with VGLUT1; only a fraction of cortical astrocytes was positive for VGLUT1 (5 of 12 tested). Note the weak correlation between VGLUT1 and VNUT1. (F) The diagrams show the pooled data (mean ± SD for the number of cells indicated in the upper graph) on the ratio of average fluorescent signal, intensity correlation quotient (ICQ), and Pearson's correlation coefficient (*Rr*) for pairs of different markers. ICQ and Rr were calculated with the aid of the intensity correlation analysis routine implemented in ImageJ and described in [65]. ICQ was calculated as a relative number of pixels with positive PDM values; Pearson's coefficient was calculated according to conventional definition. The theoretical limits for ICQ and *Rr* are ±0.5 and ±1, respectively; random staining should show ICQ and *Rr* around 0, and positive values are characteristic for dependent staining. Note the good correlation between VNUT1 and SV2A and weaker correlation between VNUT1 and lysosomal markers. Average ICQ value of correlation between VNUT1 and VGLUT1 is shown separately for five cells exhibiting good staining with VGLUT1 and for all 12 cells tested.
(TIF)

Figure S6 **Immunostaining of cortical astrocytes and neurons with synaptic and astroglial markers.** Living isolated astrocytes and neurons were cross-stained with various glial and neuronal markers to provide positive and negative control for the immunostaining procedure; the protocols of labeling and analysis were the same as in . (A) The representative two-photon fluorescence images of neocortical astrocytes and pyramidal neuron labeled with antibodies for synaptic marker PSD95 and vesicular ATP and glutamate transporters. Astrocyte and neuron shown in the middle and bottom row were from the same preparation. Note the lack of significant staining of astrocytes with anti-PSD95 contrasting with strong staining of neuron with anti-PSD95 and VGLUT1. (B) Images of astrocyte and neuron stained with antibodies to astrocyte-specific glutamate transporter protein GLT-1 and neuron-specific Neuronal Nuclei protein; cells were from the same preparation. Note the very weak labeling of astrocyte with anti-NeuN and very weak labeling of neuron with anti-GLT-1. (C, D) Good staining of astrocytes with astrocytic markers GLT-1, GFAP, and S100β confirms the efficiency of antibody delivering technique; lack of cross-staining between GLT-1 and NG2 indicates the lack of nonspecific staining. All scale bars in (A–D) are 5 µm. Raw images for panels (A–C) are uploaded as Data S2. (E) The bar diagrams show the pooled data (mean ± SD for the five cells) on the ratio of average fluorescent signal, intensity correlation quotient (ICQ), and Pearson's correlation coefficient (*Rr*) for pairs of different markers. Although there are certain limitations of usage of immunofluorescence as a quantitative approach, these data clearly demonstrate a qualitative difference in the labeling of neurons and astrocytes. The lack of staining of neurons with astroglial marker GLT-1 and very weak staining of astrocytes with PSD-95 and NeuN strongly suggest the low effect of nonspecific labeling on the immunostainig procedure used. The lack of correlation between VNUT1 and PSD-95 fluorescence supports the astrocytic origin of punctate VNUT1 staining shown in Figures S5. Good correlation between VGLUT1 and PSD-95 in neurons suggests that weak VGLUT1 labeling of astrocytes (Figure S5) was not an artifact of staining procedure.
(TIF)

Figure S7 **Functional properties of two populations of spontaneous purinergic currents in the neocortical neurons.** (A) An example of spontaneous currents recorded in the layer 2/3 pyramidal neocortical neuron wild-type mouse before and after application of glial PAR-1 receptor agonist TFLLR (10 µM) in control and after application of 3 µM NF279. Whole-cell voltage-clamp recordings were performed at a membrane potential of −80 mV in the presence of 100 µM picrotoxin, 1 µM TTX, 50 µM NBQX, and 30 µM D-AP5. Dots indicate the fast (orange) and slow (green) spontaneous currents categorized by the decay time as shown in the panel (B). Both fast and slow spontaneous currents were inhibited by selective P2X receptor antagonist NF279, supporting their purinergic nature. (C) The amplitude distributions of fast and slow purinergic currents were analyzed separately, and the corresponding waveforms (average of 20 traces) are shown in the graph on the left. In the control, the fast sEPSCs exhibited the unimodal amplitude distribution with quantal size of 9.96 pA; slow spontaneous currents had quantal size of 5.54 pA. Application of TFLLR altered neither the frequency nor the amplitude of fast currents but significantly increased the frequency of slow currents. The amplitude distribution of slow currents underwent significant changes showing the secondary peak located at double quantal size (11.2 pA); individual slow events of double quantal size are indicated in panel (A1) by double dots. (D) The pooled data on the frequency, quantal amplitude, and mean quantal content of fast and slow spontaneous currents (mean ± SD for 12 neurons). Stimulation of astrocytes with TFLLR increased the frequency and quantal content of slow currents but did not alter the fast currents. Effects of TFLLR on frequency and quantal content of slow currents were statistically significant with *p*<0.005 (one-way ANOVA). NF279 significantly reduced the quantal size and frequency of both fast and slow spontaneous currents (*p<0.01). These results support the different origin of fast and slow purinergic currents. The character of changes in the slow currents is compliant with the increase in the probability of ATP release under TFLLR.
(TIF)

Figure S8 **Apyrase inhibits nonglutamatergic sEPSCs in neocortical neurons.** (A) Left column shows the representative traces of whole-cell currents were recorded in the layer 2/3 pyramidal cortical neuron at of −80 mV in the presence of 50 µM NBQX, 30 µM D-AP5, and 100 µM picrotoxin (as a control), after application of apyrase (50 U/mL), and after application of PAR1 agonist TFLLR (10 µM) in the presence of apyrase. Right column shows the corresponding waveforms of spontaneous excitatory purinergic currents (average of 25 sEPSCs each). (B) The time course of changes in the average amplitude and frequency of nonglutamatergic sEPSCs recorded in eight pyramidal neurons during application of apyrase (50 U/mL) and TFLLR (10 µM). Dots represent mean ± SD values for mEPCS recorded in a 1-min window. Treatment with apyrase dramatically reduced the baseline amplitude and frequency of nonglutamatergic sEPSCs and prevented the TFLLR-induced burst of sEPSCs. The effects of apyrase on the frequency and amplitude of sEPSCs were statistically significant with *p*<0.01 both for baseline conditions and TFLLR application.
(TIF)

Figure S9 **Amplitude histograms for astrocyte-induced purinergic sEPSCs in the pyramidal neurons.** (A) Bar histograms show amplitude distributions build using conventional binning for the sEPSCs recorded in pyramidal neurons of wild-type mice as shown in Figure 3A. Two upper rows show examples of amplitude distributions for individual neurons before (left column) and after TFLLR application (right column). Blue and red lines show probability density functions calculated for the same datasets. Third row shows the bin histograms build for the data from all cells tested, and blue and red lines correspond to distributions, depicted in Figure 2D, scaled by the corresponding sample size. Bin size is 1 pA for all histograms. Note that amplitude distributions of sEPSCs recorded from individual wild-type neurons exhibit distinct fraction of small events, and number of small events rises dramatically under action of TFLLR. Good agreement between bin histograms and probability density functions, both in the individual cells and in the whole sample, verifies that the existence of two populations of purinergic currents was not an artifact of analysis procedure. (B) Similar comparison was made for sEPSCs recorded in the neurons of dn-SNARE mice. Note the much lower number of small currents as compared to wild-type neurons.
(TIF)

Figure S10 **Detection of ATP release in neocortex** ***in situ*** **using microelectrode biosensors.** (A) The representative responses of cortical slices of wild-type and dn-SNARE mice to the application of 10 µM TFLLR were recorded using microelectrode biosensors [19],[58] to ATP placed in the layer II/III. Each point represents mean ± SD of ATP concentration measured in the 10 s time window. (B) The pooled data on the peak of ATP transient evoked by application of TFLLR; data shown are mean ± SD for number of experiments as indicated. The difference between the average ATP responses was significant with *p*<0.01 (one-way ANOVA). In the experiments with bafilomycin A1, slices were pretreated for 2 h with 1 µM of drugs prior to recording. The significant reduction in the ATP response in the cortical slices from dn-SNARE mice and bafilomycin-treated slices strongly supports the vesicular mechanism of ATP release from astrocytes.
(TIF)

Figure S11 **Interaction between P2X and GABA receptors in the mouse neocortical neurons depends on intracellular Ca^2+^ and activity of protein kinase C.** (A) Whole-cell transmembrane currents were recorded in the acutely isolated pyramidal neuron of layer 2/3 of somatosensory cortex at a membrane potential of −80 mV. The currents were elicited by rapid application of GABA (100 µM) and nonhydrolysable ATP analogue α,β-meATP (20 µM) at time intervals indicated below. The cell was perfused with an intracellular solution containing a low concentration of Ca^2+^-chelator EGTA (0.2 mM). Note the significant decrease in the amplitude of GABA-activated current recorded 2 s after application of P2X receptor agonist. (B) GABAergic and purinergic currents were recorded using the same protocol as in (A) but with intracellular solution containing 10 mM EGTA+1 mM CaCl_2_ to clamp cytosolic Ca^2+^ at baseline level. Clamping of cytosolic Ca^2+^ eliminated the P2X receptor-triggered inhibition of GABA-evoked currents. (C) Pooled data (mean ± SD for eight cells) on the GABA receptor-mediated currents (% of control) recorded 2 s after application of P2X receptor agonists in the presence of high and low concentrations of intracellular EGTA and in the presence of protein kinase inhibitors H-89 (3 µM), KT5720 (200 nM), staurosporine (5 nM), and GF109203x (30 nM). At concentrations used, H-89 and KT5720 were selective for protein kinase A, GF109203x was selective for protein kinase C, and staurosporine was effective on both PKA and PKC. The asterisk (*) indicates the statistically significant (*p*<0.005) difference in the mean amplitude of GABA-induced current recorded before and after application of P2X receptor agonist. The significant reduction of GABA-evoked currents in the presence of low intracellular EGTA strongly supports its dependence on cytosolic Ca^2+^ elevation. Note that the reduction in the GABA-evoked current was abolished by inhibitors of PKC but not PKA. There was no statistically significant difference in the effects of ATP and its nonhydrolysable analog, suggesting that the observed effect was not mediated by phosphorylation of extracellular domain of GABA receptor by ectonuleotidases.
(TIF)

Figure S12 **Astrocyte-driven down-regulation of inhibitory transmission in the neocortical neurons depends on intracellular Ca^2+^ and activity of protein kinase C.** (A) Representative mIPSCs recorded in the pyramidal neurons of layer 2/3 before (control) and 20 s after application of PAR-1 receptor agonist TFLLR (10 µM). The whole-cell currents were recorded at a membrane potential of −40 mV using intracellular solution containing either 0.2 mM EGTA (upper traces) or 10 mM EGTA+1 mM CaCl_2_ to clamp cytosolic Ca^2+^ at physiological resting level (middle traces) or 0.2 mM EGTA and 100 nM of PKC blocker GF109203x. Extracellular medium contained 1 µM TTX, 50 µM NBQX, and 30 µM D-AP5. Outward currents were mediated by GABA receptors (as shown in Figure 6), and inward currents were mediated by P2X receptors. Activation of cortical astrocytes by TFLLR (as shown in Figure 2, Figure 3, and Figure S3) caused the marked increase in the number of inward spontaneous currents in all recordings. The significant decrease in the amplitude of outward mIPSCs was observed only when intracellular medium contained 0.2 mM EGTA. (B) The graph on the left shows the pooled data (mean ± SD) on the amplitude of mIPSCs recorded at different conditions in the number of neurons indicated in brackets. The asterisk (**) indicates the statistically significant (*p*<0.005) difference in the mean amplitude of mIPSCs recorded in control and TFLLR for 0.2 mM EGTA. The graphs on the right show changes in the frequency and amplitude of inward currents. The increase in the frequency and decrease in the mean amplitude of sEPSCs occurred due to appearance of a large number of smaller and slower sEPSCs, as shown in Figure 3 and Figure S3. There was no significant difference in the effect of TFLLR on the frequency and amplitude of sEPSCs at different conditions, verifying the independence of astroglial ATP release on intracellular perfusion of neurons. The selective attenuation of GABAergic synaptic currents in the presence of low intracellular EGTA strongly supports the postsynaptic mechanism of effect and its dependence on cytosolic Ca^2+^ elevation and activity of protein kinase C.
(TIF)

Figure S13 **Inhibition of GABA GAT3 transporters decreased the GABAergic mIPSCs and increased the tonic current in neurons of wild-type and dn-SNARE mice.** (A) Responses of acutely isolated wild-type (A_1_) and dn-SNARE (A_2_) astrocytes were activated by rapid application of 100 µM GABA in the constant presence of 100 µM picrotoxin at a membrane potential of −80 mV. The specific antagonist of glial GAT3 GABA transporters (S)-SNAP5114 (30 µM) was pre-applied 3 min before application of GABA. Diagram in panel (A_3_) shows pooled data (mean ± SD) on the density of GABA-elicited currents averaged over an indicated number of wild-type and dn-SNARE astrocytes. Significant effect of (S)-SNAP 5114 suggests the major contribution of GAT3 transporters to GABA-evoked currents. There was no significant difference in the GABA transporter-mediated current and effect of (S)-SNAP5114 between the wild-type and dn-SNARE mice. (B_1_, B_2_) Upper graph shows the time course of whole-cell transmembrane currents was recorded in the layer 2/3 neocortical pyramidal neurons of wild-type and dn-SNARE mice at −80 mV in the presence of 50 µM CNQX, 30 µM D-AP5, and 10 µM PPADS. Inhibition of astrocytic GAT3 transporters by (S)-SNAP5114 (30 µM) caused an increase in the tonic current, manifested in the downward shift of membrane holding current. Simultaneously, inhibition of GABA transporters caused a decrease in the amplitude of synaptic mIPSCs. Examples of mIPSCs, recorded at moments indicated, and average waveform of phasic GABAergic current (average of 25 mIPSCs) are shown in the inlays below. (B_3_) Diagrams show the pooled data on, from left to right, the change in the magnitude of membrane holding current, the amplitude of synaptic mIPSCs, and relative changes in the amplitudes of synaptic and tonic GABAergic currents. Data are shown as mean ± SD for the number of neurons indicated. The asterisks indicate the statistical significance of difference from the control values, (*) *p*<0.05 and (**) *p*<0.01. Note the opposite changes in the synaptic and tonic currents. There was no statistically significant difference in the effect of (S)-SNAP5114 between wild-type and dn-SNARE mice.
(TIF)

Figure S14 **Computer simulation of ATP diffusion.** To estimate the time course of ATP concentration in the extracellular space, we adapted a computer model previously used to simulate spillover of glutamate [53],[54]. The model was based on a simplified description of neurotransmitter diffusion suggested by Rusakov and Kullman [53]. The movement of ATP molecules released from the nerve terminal (A) was considered a free two-dimensional diffusion inside of the cylindrical synaptic cleft of a radius of 100 nm and height of 20 nm and a three-dimensional diffusion in the porous medium outside of the synaptic cleft. Movement of ATP after release from the glial site (B) was also calculated as a three-dimensional diffusion in the porous medium with the following parameters: diffusion coefficient D = 0.2 µm/ms (50% lower than for glutamate diffusion), free volume factor 0.12, and tortuosity factor of 1.34. To account for the quick breakdown of ATP by ectonucleotidases, the kinetic scheme for transporter uptake was replaced by Michaelis–Menten kinetics of ATP->ADP conversion with V_max_ and K_M_ values of 2.2 µM/s and 33 µM [63]. In both cases, the conservative estimate (i.e., longer travel) of linear spine density as 1 spine/µm [46] was used. (A) Graph shows simulated time course for ATP concentration after release of 1,000 molecules from the vesicle at the centre of synaptic cleft (r = 0) for the different distances: middle of synaptic cleft (r = 0.05 µm), edge of synaptic cleft (r = 0.1 µm), and middle of typical intersynaptic distance (0.5 µm).(B) Graph shows simulated time course for ATP concentration after release of 1,000 molecules from ectopic (glial) site located at the middle of the intersynaptic distance, for the following locations: vicinity of the release site (r = 0.05 µm), edge of the nearest synapse (r = 0.5 µm), and centre of nearest synapse (r = 0.6 µm). Red horizontal line in both graphs indicates a minimal level of ATP that can reliably activate P2X2 and P2X4 receptors, set as 10% of EC50 (highest estimate for both receptors is 10 µM). Note that release of 1,000 ATP molecules from ectopic site is sufficient to activate extrasynaptic (r = 0.05) and perisynaptic (r = 0.5) but cannot reliably activate receptors inside synaptic cleft (because of hampered diffusion from extracellular space into the narrow cleft).
(TIF)

Figure S15 **Computer simulation of P2X receptor response after synaptic and ectopic release of ATP.** The simulation of net response (calculated as a net conductance) of synaptic and extrasynaptic P2X receptors located within 0.5 µM of centre of synaptic cleft. The same model of ATP release and diffusion was used as in Figure S14. To account for preferential location of the P2X receptor on the periphery of the synapse, their density was set as 5×10^−3^/nm^2^ within the radius of r = 0.1±0.01 (periphery of dendritic spine) nm and 5×10^−4^/nm^2^ elsewhere. This is a conservative estimate since maximal density is set at less than 1 receptor per 10 nm. The equal proportion of P2X2 and P2X4 receptors was assumed. Kinetic model of receptor activation used in [54] was replaced by the simplified model of P2X2 receptor kinetics [42],[66] and was used for both receptors, with an activation time of P2X4 receptors decreased to 8.8 ms accordingly to [67]; maximal conductance and open probability for the P2X2 and P2X4 receptors were set correspondingly as 21 and 9 pS and 0.6 and 0.2 [39],[64],[67]. Synaptic release (black and blue lines): the response to release of 1,000 ATP molecules from the vesicle at the central part of (r = 0) and peripheral part of synaptic cleft (r = 0.05). Glial release (orange and red lines): the response to vesicular release of 1,000 ATP molecules from the glial site located at the middle of the intrasynaptic space of (r = 0.05) and near the peripheral part of synaptic cleft (r = 0.5). The response to synaptic release does not show strong dependence on distance to release site (A), resulting from the saturation of receptors. Response to glial release does not show steep dependence either, and this can be explained by the decrease in the ATP concentration at r = 0.5 (edge of synapse) that is compensated by an increase in the density of receptors. Note that the simulated P2X responses to glial release have smaller amplitudes and slower kinetics than responses to the synaptic release.
(TIF)

Figure S16 **Whole-cell recording from pyramidal neocortical neuron of dn-SNARE mouse.** Images show the typical outline of experiments described in Figures 6–8. The two-photon image of EGFP fluorescence of dn-SNARE astrocytes (maximal projection of Z-stack) is merged with the fluorescent image of the pyramidal neuron perfused with intracellular solution containing fluorescent dye Texas Red (20 µM). Note the three green dn-SNARE-expressing astrocytes in the immediate vicinity of pyramidal neuron.
(TIF)

Table S1 **Properties of WT and dn-SNARE astrocytes.**
(PDF)

Table S2 **Action of bafilomycin A1 on phasic purinergic currents in the neocortical neurons in situ.**
(PDF)

Text S1 **Limitations of immunostaining of living astrocytes.**
(PDF)

## Abbreviations

AP3A: di(adenosine 5′)-triphosphate
DIDS: 4,4′-diisothiocyanostilbene-2,2′-disulfonic acid
EPCS: excitatory postsynaptic current
HFS: high-frequency stimulation
IPSC: inhibitory postsynaptic current
NP-EGTA: *o*-nitrophenyl-EGTA-AM
NPPB: 5-nitro-2-(3-phenylpropylamino)benzoic acid
PPADS: pyridoxalphosphate-6-azophenyl-2′-4′-disulphonic acid
PPR: paired-pulse ratio
VGLUT1: vesicular glutamate transporter (type 1)
VNUT1: vesicular nucleotide transporter (type 1)

## References

[1] Burnstock G (2007) Physiology and pathophysiology of purinergic neurotransmission. Physiol Rev 87: 659–797. 17429044 10.1152/physrev.00043.2006

[2] Abbracchio MP, Burnstock G, Verkhratsky A, Zimmermann H (2009) Purinergic signalling in the nervous system: an overview. Trends Neurosci 32: 19–29. 19008000 10.1016/j.tins.2008.10.001

[3] Guthrie PB, Knappenberger J, Segal M, Bennett MV, Charles AC, et al (1999) ATP released from astrocytes mediates glial calcium waves. J Neurosci 19: 520–528. 9880572 10.1523/JNEUROSCI.19-02-00520.1999 PMC6782195

[4] Fields RD, Burnstock G (2006) Purinergic signalling in neuron-glia interactions. Nat Rev Neurosci 7: 423–436. 16715052 10.1038/nrn1928 PMC2062484

[5] Lalo U, Pankratov Y, Parpura V, Verkhratsky A (2010) Ionotropic receptors in neuronal-astroglial signalling: what is the role of “excitable” molecules in non-excitable cells. Biochim Biophys Acta 1813: 992–1002. 20869992 10.1016/j.bbamcr.2010.09.007

[6] Palygin O, Lalo U, Verkhratsky A, Pankratov Y (2010) Ionotropic NMDA and P2X1/5 receptors mediate synaptically induced Ca2+ signalling in cortical astrocytes. Cell Calcium 48: 225–231. 20926134 10.1016/j.ceca.2010.09.004

[7] Pearson RA, Dale N, Llaudet E, Mobbs P (2005) ATP released via gap junction hemichannels from the pigment epithelium regulates neural retinal progenitor proliferation. Neuron 46: 731–744. 15924860 10.1016/j.neuron.2005.04.024

[8] Di Virgilio F, Ceruti S, Bramanti P, Abbracchio MP (2009) Purinergic signalling in inflammation of the central nervous system. Trends Neurosci 32: 79–87. 19135728 10.1016/j.tins.2008.11.003

[9] Halassa MM, Fellin T, Haydon PG (2007) The tripartite synapse: roles for gliotransmission in health and disease. Trends Mol Med 13: 54–63. 17207662 10.1016/j.molmed.2006.12.005

[10] Gordon GR, Iremonger KJ, Kantevari S, Ellis-Davies GC, MacVicar BA, et al (2009) Astrocyte-mediated distributed plasticity at hypothalamic glutamate synapses. Neuron 64: 391–403. 19914187 10.1016/j.neuron.2009.10.021 PMC4107870

[11] Pankratov YV, Lalo UV, Krishtal OA (2002) Role for P2X receptors in long-term potentiation. J Neurosci 22: 8363–8369. 12351710 10.1523/JNEUROSCI.22-19-08363.2002 PMC6757784

[12] Pascual O, Casper KB, Kubera C, Zhang J, Sul JY, et al (2005) Astrocytic purinergic signaling coordinates synaptic networks. Science 310: 113–116. 16210541 10.1126/science.1116916

[13] Pankratov Y, Lalo U, Verkhratsky A, North RA (2007) Quantal release of ATP in mouse cortex. J Gen Physiol 129: 257–265. 17325196 10.1085/jgp.200609693 PMC2151610

[14] Jo YH, Schlichter R (1999) Synaptic corelease of ATP and GABA in cultured spinal neurons. Nat Neurosci 2: 241–245. 10195216 10.1038/6344

[15] Garre JM, Retamal MA, Cassina P, Barbeito L, Bukauskas FF, et al (2010) FGF-1 induces ATP release from spinal astrocytes in culture and opens pannexin and connexin hemichannels. PNAS 107: 22659–22664. 21148774 10.1073/pnas.1013793107 PMC3012468

[16] Hamilton NB, Attwell D (2010) Do astrocytes really exocytose neurotransmitters? Nat Rev Neurosci 11: 227–238. 20300101 10.1038/nrn2803

[17] Volterra A, Meldolesi J (2005) Astrocytes, from brain glue to communication elements: the revolution continues. Nat Rev Neurosci 6: 626–640. 16025096 10.1038/nrn1722

[18] Carmignoto G, Haydon PG (2012) Astrocyte calcium signaling and epilepsy. Glia 60: 1227–1233. 22389222 10.1002/glia.22318 PMC4532388

[19] Gourine AV, Kasymov V, Marina N, Tang F, Lane S, et al (2010) Astrocytes control breathing through pH-dependent release of ATP. Science 329: 571–575. 20647426 10.1126/science.1190721 PMC3160742

[20] Halassa MM, Florian C, Fellin T, Munoz JR, Lee SY, et al (2009) Astrocytic modulation of sleep homeostasis and cognitive consequences of sleep loss. Neuron 61: 213–219. 19186164 10.1016/j.neuron.2008.11.024 PMC2673052

[21] Bal-Price A, Moneer Z, Brown GC (2002) Nitric oxide induces rapid, calcium-dependent release of vesicular glutamate and ATP from cultured rat astrocytes. Glia 40: 312–323. 12420311 10.1002/glia.10124

[22] Bezzi P, Gundersen V, Galbete JL, Seifert G, Steinhauser C, et al (2004) Astrocytes contain a vesicular compartment that is competent for regulated exocytosis of glutamate. Nat Neurosci 7: 613–620. 15156145 10.1038/nn1246

[23] Li D, Ropert N, Koulakoff A, Giaume C, Oheim M (2008) Lysosomes are the major vesicular compartment undergoing Ca2+-regulated exocytosis from cortical astrocytes. J Neurosci 28: 7648–7658. 18650341 10.1523/JNEUROSCI.0744-08.2008 PMC6670856

[24] Marchaland J, Cali C, Voglmaier SM, Li H, Regazzi R, et al (2008) Fast subplasma membrane Ca2+ transients control exo-endocytosis of synaptic-like microvesicles in astrocytes. J Neurosci 28: 9122–9132. 18784293 10.1523/JNEUROSCI.0040-08.2008 PMC2846455

[25] Montana V, Ni Y, Sunjara V, Hua X, Parpura V (2004) Vesicular glutamate transporter-dependent glutamate release from astrocytes. J Neurosci 24: 2633–2642. 15028755 10.1523/JNEUROSCI.3770-03.2004 PMC6729507

[26] Sawada K, Echigo N, Juge N, Miyaji T, Otsuka M, et al (2008) Identification of a vesicular nucleotide transporter. PNAS 105: 5683–5686. 18375752 10.1073/pnas.0800141105 PMC2311367

[27] Agulhon C, Fiacco TA, McCarthy KD (2010) Hippocampal short- and long-term plasticity are not modulated by astrocyte Ca2+ signaling. Science 327: 1250–1254. 20203048 10.1126/science.1184821

[28] Takata N, Mishima T, Hisatsune C, Nagai T, Ebisui E, et al (2011) Astrocyte calcium signaling transforms cholinergic modulation to cortical plasticity in vivo. J Neurosci 31: 18155–18165. 22159127 10.1523/JNEUROSCI.5289-11.2011 PMC6634158

[29] Navarrete M, Perea G, Fernandez de Sevilla D, Gomez-Gonzalo M, Nunez A, et al (2012) Astrocytes mediate in vivo cholinergic-induced synaptic plasticity. PLoS Biol 10: e1001259 doi:10.1371/journal.pbio.1001259 22347811 10.1371/journal.pbio.1001259 PMC3279365

[30] Chen N, Sugihara H, Sharma J, Perea G, Petravicz J, et al (2012) Nucleus basalis-enabled stimulus-specific plasticity in the visual cortex is mediated by astrocytes. Proc Natl Acad Sci U S A 109: E2832–E2841. 23012414 10.1073/pnas.1206557109 PMC3478642

[31] Woo DH, Han KS, Shim JW, Yoon BE, Kim E, et al (2012) Trek-1 and Best1 channels mediate fast and slow glutamate release in astrocytes upon GPRC activation. Cell 151: 25–40. 23021213 10.1016/j.cell.2012.09.005

[32] Li D, Herault K, Silm K, Evrard A, Wojcik S, et al (2013) Lack of evidence for vesicular glutamate transporter expression in mouse astrocytes. J Neurosci 33: 4434–4455. 23467360 10.1523/JNEUROSCI.3667-12.2013 PMC6704936

[33] Martineau M, Shi T, Puyal J, Knolhoff AM, Dulong J, et al (2013) Storage and uptake of D-serine into astrocytic synaptic-like vesicles specify gliotransmission. J Neurosci 33: 3413–3423. 23426669 10.1523/JNEUROSCI.3497-12.2013 PMC3772647

[34] Lalo U, Pankratov Y, Kirchhoff F, North RA, Verkhratsky A (2006) NMDA receptors mediate neuron-to-glia signaling in mouse cortical astrocytes. J Neurosci 26: 2673–2683. 16525046 10.1523/JNEUROSCI.4689-05.2006 PMC6675155

[35] Shigetomi E, Bowser DN, Sofroniew MV, Khakh BS (2008) Two forms of astrocyte calcium excitability have distinct effects on NMDA receptor-mediated slow inward currents in pyramidal neurons. J Neurosci 28: 6659–6663. 18579739 10.1523/JNEUROSCI.1717-08.2008 PMC2866443

[36] Lee CJ, Mannaioni G, Yuan H, Woo DH, Gingrich MB, et al (2007) Astrocytic control of synaptic NMDA receptors. J Physiol 581: 1057–1081. 17412766 10.1113/jphysiol.2007.130377 PMC2170820

[37] Heurteaux C, Lucas G, Guy N, El Yacoubi M, Thummler S, et al (2006) Deletion of the background potassium channel Trek-1 results in a depression-resistant phenotype. Nat Neurosci 9: 1134–1141. 16906152 10.1038/nn1749

[38] Marsey LL, Winpenny JP (2010) Bestrophin expression and function in the human pancreatic duct cell line, Cfpac-1. J Physiol 587: 2211–2224. 10.1113/jphysiol.2008.159087 PMC2697294 19237432

[39] Gu JG, Bardoni R, Magherini PC, MacDermott AB (1998) Effects of the P2-purinoceptor antagonists suramin and pyridoxal-phosphate-6-azophenyl-2′,4′-disulfonic acid on glutamatergic synaptic transmission in rat dorsal horn neurons of the spinal cord. Neurosci Lett 253: 167–170. 9792237 10.1016/s0304-3940(98)00632-6

[40] Rettinger J, Schmalzing G, Damer S, Muller G, Nickel P, et al (2000) The suramin analogue NF279 is a novel and potent antagonist selective for the P2X(1) receptor. Neuropharmacology 39: 2044–2053. 10963748 10.1016/s0028-3908(00)00022-8

[41] Suzuki E, Kessler M, Montgomery K, Arai AC (2004) Divergent effects of the purinoceptor antagonists suramin and pyridoxal-5′-phosphate-6-(2′-naphthylazo-6′-nitro-4′,8′-disulfonate) (PPNDS) on alpha-amino-3-hydroxy-5-methyl-4-isoxazolepropionic acid (AMPA) receptors. Mol Pharmacol 66: 1738–1747. 15448189 10.1124/mol.104.003038

[42] North RA (2002) Molecular physiology of P2X receptors. Physiol Rev 82: 1013–1067. 12270951 10.1152/physrev.00015.2002

[43] Sim J, Chaumont S, Jo J, Ulmann L, Young M, Cho K, et al (2006) Altered hippocampal synaptic potentiation in P2X4 knock-out mice. J Neurosci 26: 9006–9009. 16943557 10.1523/JNEUROSCI.2370-06.2006 PMC6675341

[44] Khakh BS, North RA (2012) Neuromodulation by extracellular ATP and P2X receptors in the CNS. Neuron 76: 51–69. 23040806 10.1016/j.neuron.2012.09.024 PMC4064466

[45] Panatier A, Vallee J, Haber M, Murai KK, Lacaille JC, et al (2011) Astrocytes are endogenous regulators of basal transmission at central synapses. Cell 146: 785–798. 21855979 10.1016/j.cell.2011.07.022

[46] Halassa MM, Fellin T, Takano H, Dong JH, Haydon PG (2007) Synaptic islands defined by the territory of a single astrocyte. J Neurosci 27: 6473–6477. 17567808 10.1523/JNEUROSCI.1419-07.2007 PMC6672436

[47] Jacob TC, Moss SJ, Jurd R (2008) GABA(A) receptor trafficking and its role in the dynamic modulation of neuronal inhibition. Nat Rev Neurosci 9: 331–343. 18382465 10.1038/nrn2370 PMC2709246

[48] Kittler JT, Moss SJ (2003) Modulation of GABAA receptor activity by phosphorylation and receptor trafficking: implications for the efficacy of synaptic inhibition. Curr Opin Neurobiol 13: 341–347. 12850219 10.1016/s0959-4388(03)00064-3

[49] Khakh BS, Gittermann D, Cockayne DA, Jones A (2003) ATP modulation of excitatory synapses onto interneurons. J Neurosci 23: 7426–7437. 12917379 10.1523/JNEUROSCI.23-19-07426.2003 PMC6740451

[50] Semyanov A, Walker MC, Kullmann DM, Silver RA (2004) Tonically active GABA A receptors: modulating gain and maintaining the tone. Trends Neurosci 27: 262–269. 15111008 10.1016/j.tins.2004.03.005

[51] Glykys J, Mody I (2007) Activation of GABAA receptors: views from outside the synaptic cleft. Neuron 56: 763–770. 18054854 10.1016/j.neuron.2007.11.002

[52] Shigetomi E, Tong X, Kwan KY, Corey DP, Khakh BS (2012) TRPA1 channels regulate astrocyte resting calcium and inhibitory synapse efficacy through GAT-3. Nat Neurosci 15: 70–80. 10.1038/nn.3000 PMC3282183 22158513

[53] Rusakov DA, Kullmann DM (1998) Extrasynaptic glutamate diffusion in the hippocampus: ultrastructural constraints, uptake, and receptor activation. J Neurosci 18: 3158–3170. 9547224 10.1523/JNEUROSCI.18-09-03158.1998 PMC6792642

[54] Pankratov YV, Krishtal OA (2003) Distinct quantal features of AMPA and NMDA synaptic currents in hippocampal neurons: implication of glutamate spillover and receptor saturation. Biophys J 85: 3375–3387. 14581239 10.1016/S0006-3495(03)74757-2 PMC1303615

[55] Frenguelli BG, Wigmore G, Llaudet E, Dale N (2007) Temporal and mechanistic dissociation of ATP and adenosine release during ischaemia in the mammalian hippocampus. J Neurochem 101: 1400–1413. 17459147 10.1111/j.1471-4159.2006.04425.x PMC1920548

[56] Rubio ME, Soto F (2001) Distinct Localization of P2X receptors at excitatory postsynaptic specializations. J Neurosci 21: 641–653. 11160443 10.1523/JNEUROSCI.21-02-00641.2001 PMC6763822

[57] Richler E, Shigetomi E, Khakh BS (2011) Neuronal P2X2 receptors are mobile ATP sensors that explore the plasma membrane when activated. J Neurosci 31: 16716–16730. 22090499 10.1523/JNEUROSCI.3362-11.2011 PMC3282184

[58] Huckstepp RT, id Bihi R, Eason R, Spyer KM, Dicke N, et al (2010) Connexin hemichannel-mediated CO2-dependent release of ATP in the medulla oblongata contributes to central respiratory chemosensitivity. J Physiol 588: 3901–3920. 20736421 10.1113/jphysiol.2010.192088 PMC3000581

[59] Lalo U, Palygin O, North RA, Verkhratsky A, Pankratov Y (2011) Age-dependent remodelling of ionotropic signalling in cortical astroglia. Aging Cell 10: 392–402. 21272193 10.1111/j.1474-9726.2011.00682.x

[60] Brandon NJ, Jovanovic JN, Smart TG, Moss SJ (2002) Receptor for activated C kinase-1 facilitates protein kinase C-dependent phosphorylation and functional modulation of GABA(A) receptors with the activation of G-protein-coupled receptors. J Neurosci 22: 6353–6361. 12151513 10.1523/JNEUROSCI.22-15-06353.2002 PMC6758144

[61] Ding S, Fellin T, Zhu Y, Lee SY, Auberson YP, et al (2007) Enhanced astrocytic Ca2+ signals contribute to neuronal excitotoxicity after status epilepticus. J Neurosci 27: 10674–10684. 17913901 10.1523/JNEUROSCI.2001-07.2007 PMC2917229

[62] Torres A, Wang F, Xu Q, Fujita T, Dobrowolski R, et al (2012) Extracellular Ca2+ acts as a mediator of communication from neurons to glia. Science Sig 5 (208) ra8. 10.1126/scisignal.2002160 PMC3548660 22275221

[63] Henneberger C, Papouin T, Oliet SH, Rusakov DA (2010) Long-term potentiation depends on release of D-serine from astrocytes. Nature 463: 232–236. 20075918 10.1038/nature08673 PMC2807667

[64] Nolte C, Matyash M, Pivneva T, Schipke CG, Ohlemeyer C, et al (2001) GFAP promoter-controlled EGFP-expressing transgenic mice: a tool to visualize astrocytes and astrogliosis in living brain tissue. Glia 33: 72–86. 11169793

[65] Li Q, Lau A, Morris TJ, Guo L, Fordyce CB, Stanley EF (2004) A Syntaxin 1, Gα_o_, and N-type calcium channel complex at a presynaptic nerve terminal: analysis by quantitative immunocolocalization. J Neurosci 24 (16) 4070–4081. 15102922 10.1523/JNEUROSCI.0346-04.2004 PMC6729428

[66] Ding S, Sachs F (1999) Single channel properties of P2X2 purinoceptors. J Gen Physiol 113: 695–720. 10228183 10.1085/jgp.113.5.695 PMC2222910

[67] Yan Z, Liang Z, Obsil T, Stojilkovic SS (2006) Participation of the Lys313-Ile333 sequence of the purinergic P2X4 receptor in agonist binding and transduction of signals to the channel gate. J Biol Chem 281: 32649–32659. 16954225 10.1074/jbc.M512791200

