# Correction: Exocytosis of ATP From Astrocytes Modulates Phasic and Tonic Inhibition in the Neocortex

**DOI:** 10.1371/journal.pbio.1001857

**Published:** 2014-04-14

**Authors:** The PLOS Biology Staff

The authors noticed an error in Panel 2A of Figure 2, and they have now provided a corrected version here.

**Figure 2:**
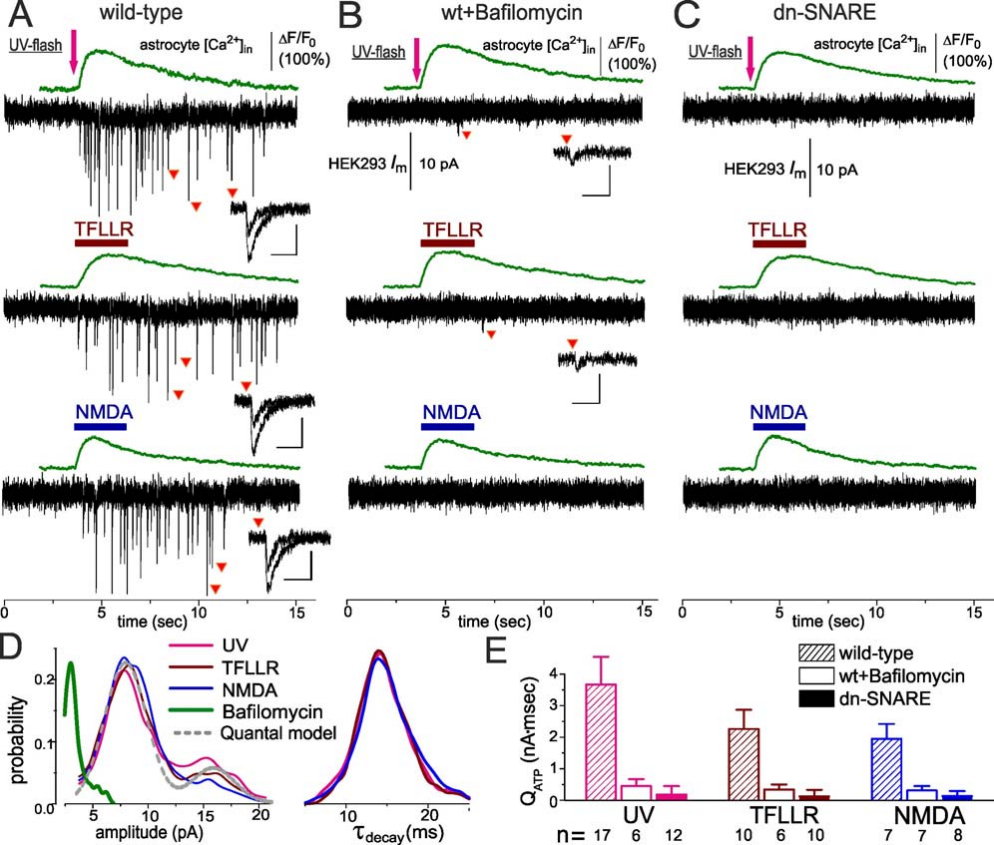
Ca2+-dependent release of ATP can be evoked in the cortical astrocytes of the wild-type but not of the dn-SNARE mice. Release of ATP from cortical astrocytes of wild-type (A, B) and dn-SNARE mice (C) was detected using the sniffer cells as described in Figure 1. (A) Elevation of cytosolic Ca2+ level was elicited in the astrocytes by UV uncaging and by rapid application of the agonist of PAR-1 metabotropic receptor TFLLR (10 µM) or the agonist of glutamate ionotropic receptor NMDA (20 µM). (B) Inhibition of vacuolar H-ATPase in the astrocytes with Bafilomycin A1 (1 µM for 2 h) dramatically decreased both the amplitude and frequency of phasic currents. (C) Elevation of the Ca2+ level in any of the dn-SNARE astrocytes did not lead to activation of phasic purinergic currents in the sniffer cell. Inlays in (A–C) show examples of individual phasic currents recorded at moments indicated; scale bars are 50 ms and 10 pA. (D) The amplitude and decay time distributions of purinergic currents recorded in the HEK293-P2X2 cells after stimulation of the astrocytes; data were pooled for number of experiments indicated in (E). The grey dotted line shows the best fit of quantal model to the distribution of UV-activated currents. (E) The pooled data (mean ± SD for indicated numbers of experiments) on net release of ATP were assessed as total charge transferred by spontaneous currents in the sniffer cell. The effects of bafylomicin and dn-SNARE expression on net charge transferred by purinergic currents were statistically significant at p  =  0.005 (two-population t test).
