# Supplementary figures and images for: Exocytosis of ATP From Astrocytes Modulates Phasic and Tonic Inhibition in the Neocortex
Source: PLoS Biol. 2014 Jan 7;12(1):e1001747. doi: 10.1371/journal.pbio.1001747 (PMC3883644; doi:10.1371/journal.pbio.1001747)

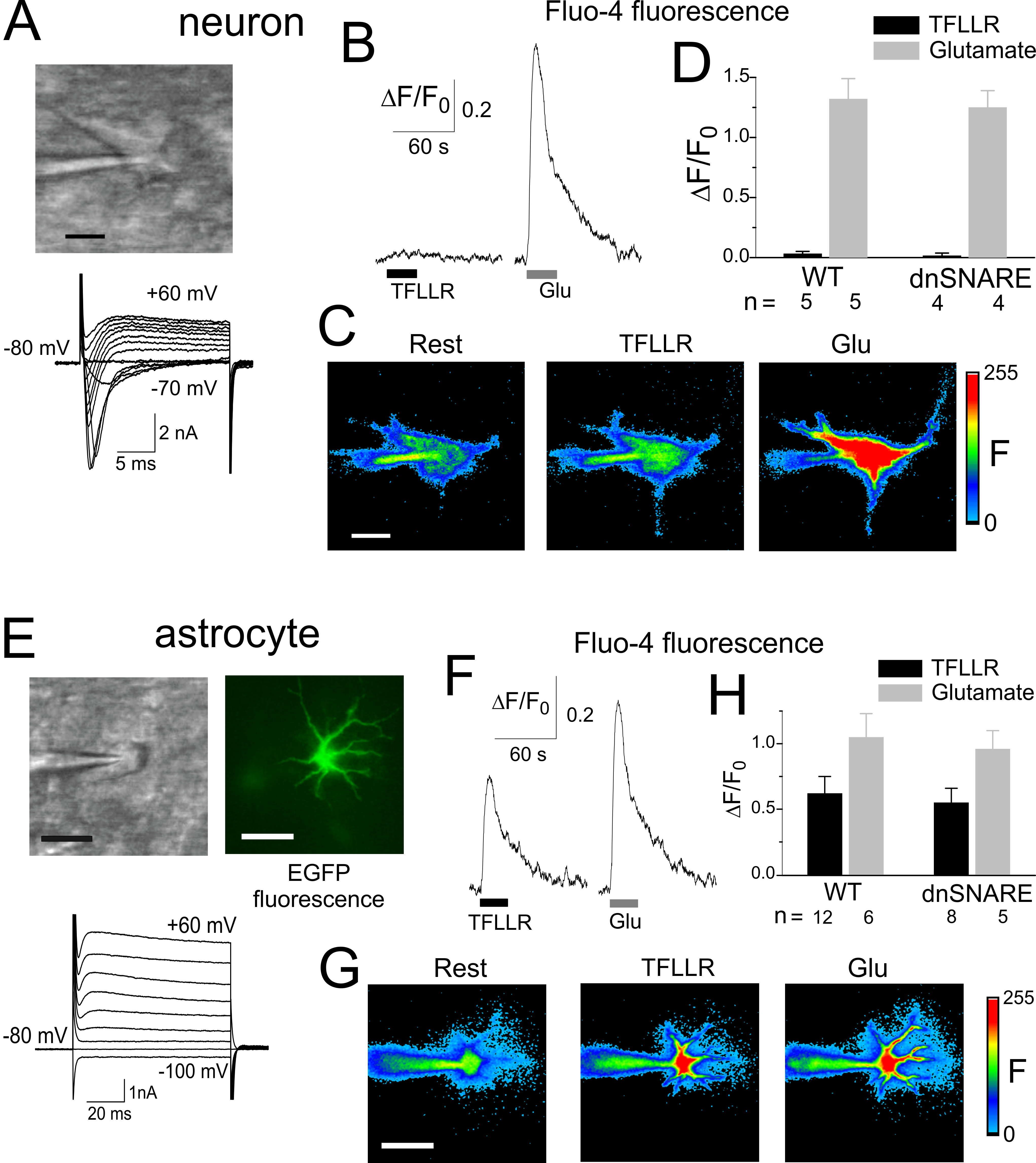

Supplement: Figure S1 — Differential action of PAR-1 receptor agonist in the pyramidal neurons and astrocytes of neocortex. The pyramidal neurons (A–C) and astrocytes (E–G) of somatosensory cortex layer 2/3 of wild-type and dn-SNARE mice were loaded with Ca2+ indicator Fluo-4 via patch-pipette. Ca2+-signals were evoked in neurons and astrocytes by a 30-s-long rapid bath application of 30 µM TFLLR and 100 µM L-Glutamate to cortical slices. The membrane holding potential during Ca2+ measurements was −40 mV in the neurons and −80 mV in the astrocytes. (A) The representative gradient-contrast image and (below) electrophysiological characterization of cortical pyramidal neurons of dn-SNARE mice; the high input resistance and fast voltage-gated Na+ current can be seen. (B) The representative Ca2+ transients evoked in the neuron of dn-SNARE mouse by application of TFLLR and glutamate; the panel (C) shows representative pseudocolor fluorescent images recorded at rest and at the peak of Ca2+ transients as indicated. Panel (D) shows the pooled data (mean ± SD for number of neurons indicated) of peak Ca2+ elevation; the difference between TFLLR and glutamate-evoked response was statistically significant with (*) p = 0.004 (one-way ANOVA). The PAR-1 receptors did not elicit the notable rise in the intracellular Ca2+ level in cortical pyramidal neurons of both wild-type and dn-SNARE mice. (E) The gradient contrast and EGFP fluorescence images and electrophysiological characterization of cortical astrocyte of dn-SNARE mouse; the low input resistance and large K+ current can be seen. Panels (F) and (G) show the elevation in the intracellular Ca2+ level evoked in the cortical astrocyte of dn-SNARE mouse by application of TFLLR and glutamate. Panel (H) shows the pooled data (mean ± SD for number of astrocytes indicated) of peak Ca2+ elevation; the was no significant difference between TFLLR-evoked response in the wild-type and dn-SNARE mice (one-way ANOVA). The PAR-1 receptors mediated substantial Ca2+ ele [file pbio.1001747.s003.tif]

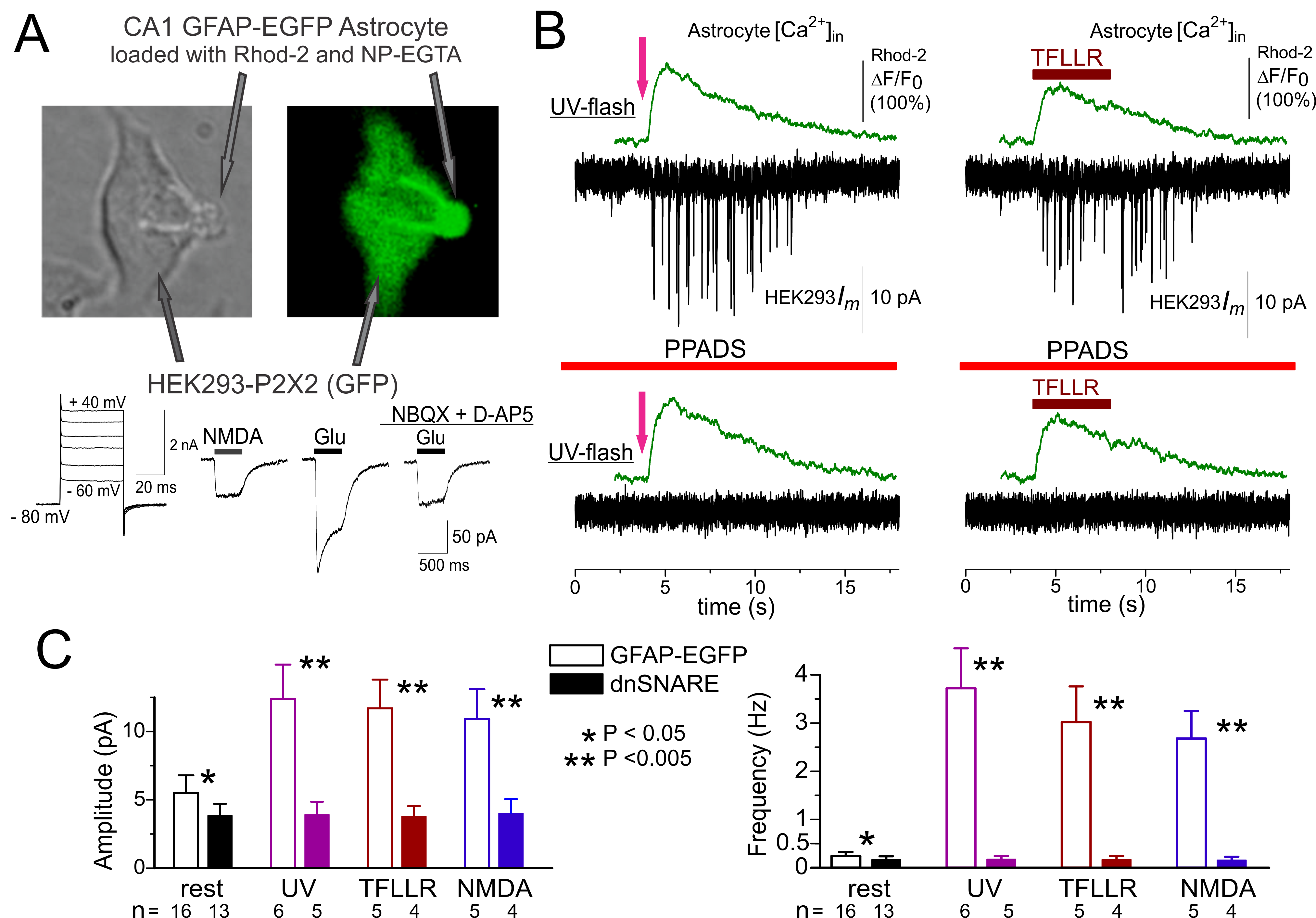

Supplement: Figure S2 — Detection of ATP released from the hippocampal CA1 astrocytes with the aid of sniffer cells. (A) The astrocytes acutely dissociated from the CA1 hippocampal area of the brain slice of EGFP-GFAP mouse [61] have been resuspended, loaded with UV-photoliable Ca2+-chelator NP-EGTA and Ca2+-indicator Rhod-2 AM, and placed over cultured HEK293 cells expressing P2X2 receptors. Lower panel shows functional characterization of astrocytes, using voltage-clamp recordings (performed after the uncaging experiment). From left to right, whole-cell currents activated by the series of depolarizing steps from a holding potential of −80 mV and currents evoked by fast application of 20 µM NMDA and 100 µM glutamate at −80 mV. The current evoked by glutamate in the presence of 30 µM NBQX and 50 µM D-AP5 is mediated by glutamate transporters. (B) Rhod-2 fluorescent signals have been monitored in the hippocampal astrocytes simultaneously with whole-cell recording of transmembrane current in HEK293 cells voltage-clamped at −80 mV in the control (upper panels) and in the presence of 10 µM PPADS (low panels). Flash of UV-light (365 nM) caused an elevation of cytosolic Ca2+ in the astrocyte followed by the burst of phasic currents in the HEK293 cell; a few spontaneous events could be observed in control in the absence of UV illumination. Similar burst of activity in the sniffer cell was induced by the specific agonist of astroglial PAR-1 receptors (TFLLR). Both the baseline and UV-elicited spurious currents were strongly inhibited (decrease in the amplitude was 89%±6% and 93%±5%, respectively; n = 4) after application of P2X receptor antagonist PPADS, confirming that they were mediated by receptors to ATP. (C) The amplitude and frequency of spurious currents measured in the HEK293 cell before (over 10 min time window) and after stimulation of astrocytes isolated from hippocampal CA1 region of GFAP-EGFP and dn-SNARE mice by UV-flash and application of 10 µM TFLLR and 20 µM NMDA (8 s time window) [file pbio.1001747.s004.tif]

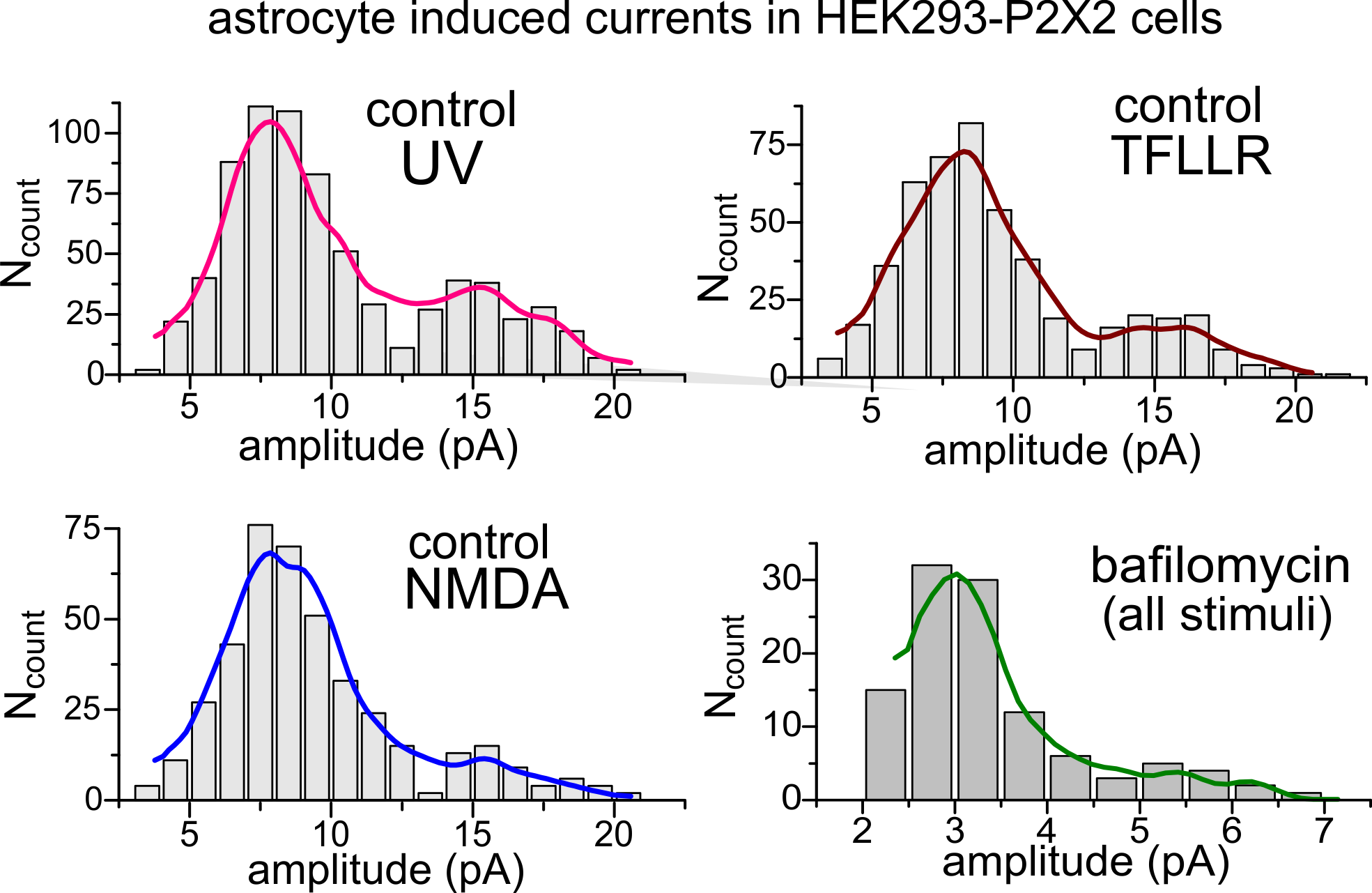

Supplement: Figure S3 — Amplitude histograms for astrocyte-induced purinergic currents in the HEK293-P2X2. Bar histograms show amplitude distributions built by conventional binning method for the same datasets as distributions shown in Figure 2D; bin size was 1 pA for currents recorded in the control and 0.5 pA for bafilomycin. Solid (color) lines correspond to distributions, depicted in Figure 2D, scaled by the corresponding sample size. Note that the distributions shown in Figures 2D are probability density functions, calculated using independent method as described in [51], rather than smoothed bin histograms. Probability density functions do not use amplitude binning. Good agreement between bin histograms and probability density functions verifies that quantal pattern of sniffer cell currents was not an artifact of analysis procedure. (TIF) [file pbio.1001747.s005.tif]

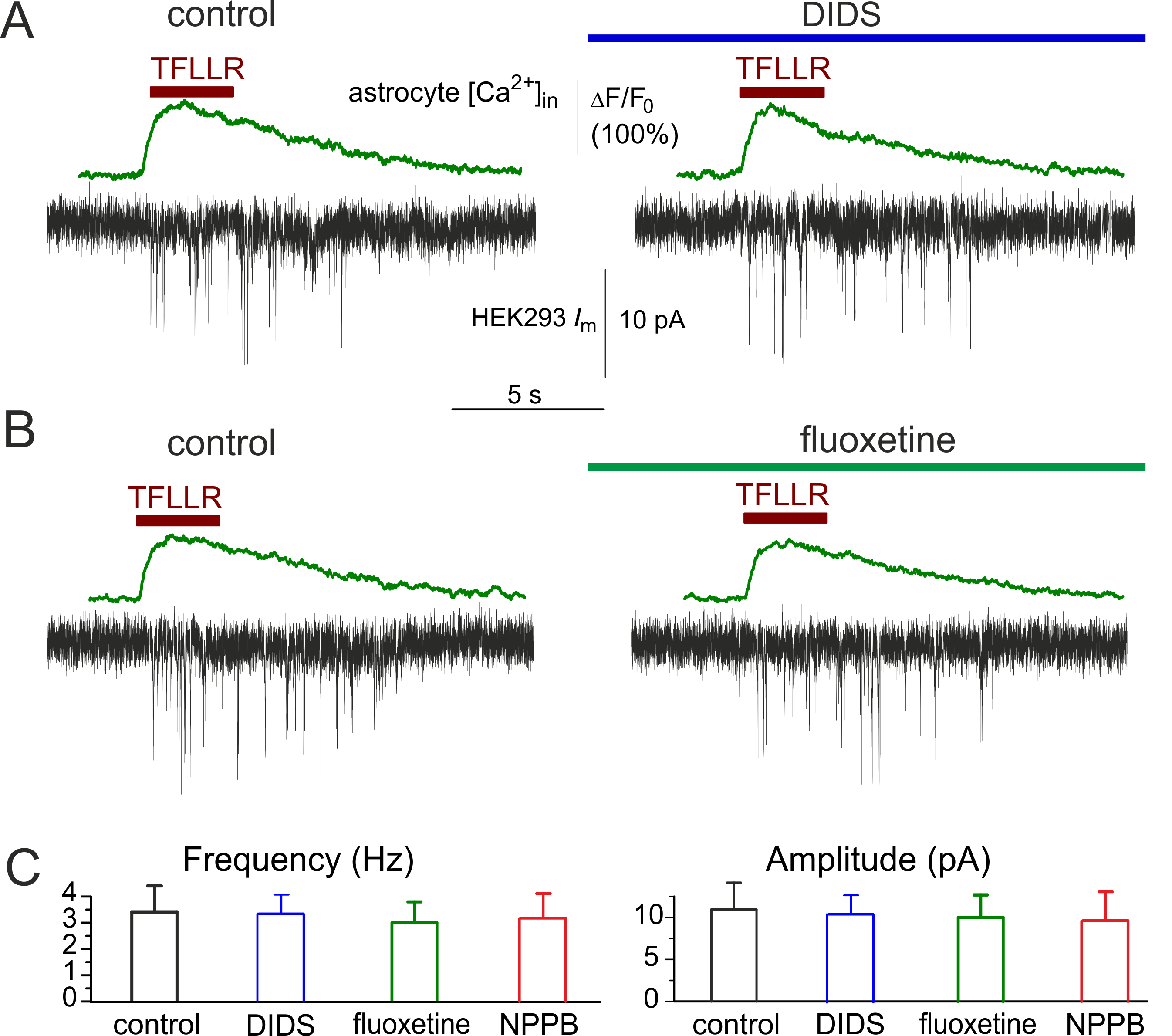

Supplement: Figure S4 — Lack of contribution of TREK-1 and Best1 channels to the release of ATP from cortical astrocytes. Release of ATP from cortical astrocytes of wild-type mice was detected using the sniffer cells as described in Figures 1 and 2. Elevation of the cytosolic Ca2+ level was elicited in the astrocytes by rapid application of agonist PAR-1 metabotropic receptor TFLLR (10 µM) in control and in the presence of inhibitor of TREK-1 potassium channels fluoxetine (100 µM) and inhibitors of Ca2+-sensitive chloride channels DIDS (300 µM) and NPPB (100 µM). The latter inhibitors efficiently block best1 channels. (A, B) Representative astrocytic Ca2+ transients and recordings of transmembrane currents in HEK293-P2X2 cell voltage-clamped at −80 mV. (C) The diagrams show the mean amplitude and frequency of phasic currents in the sniffer cells (mean ± SD for 15 cells in the control and 5 cells for each inhibitor). Note the lack of effect of TREK-1 and Best1 channels blockers on astrocyte-driven purinergic currents in the sniffer cells. (TIF) [file pbio.1001747.s006.tif]

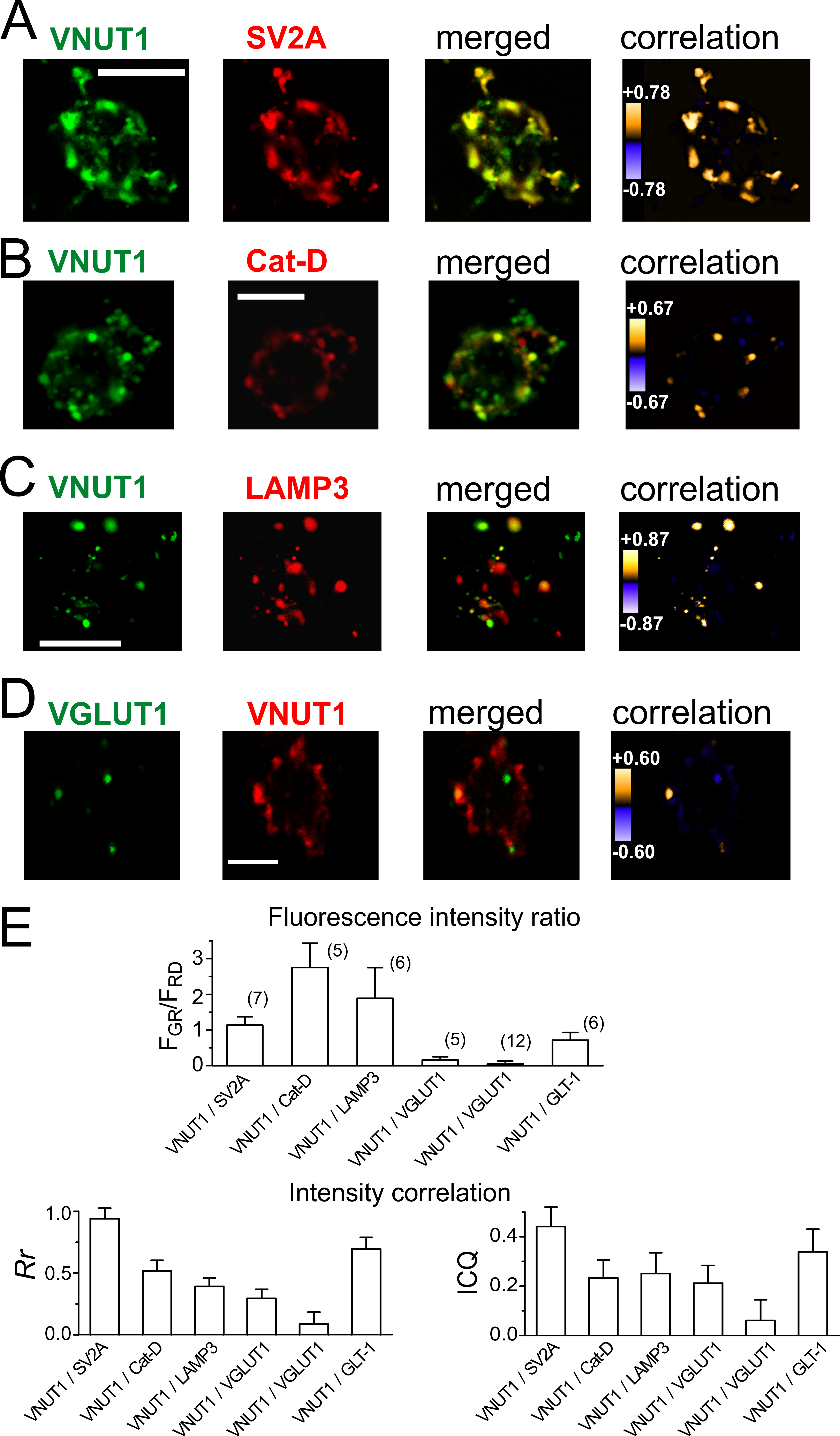

Supplement: Figure S5 — Colocalization of vesicular ATP transporters with exocytotic organelle markers. Living, acutely isolated cortical astrocytes were labeled with antibodies to vesicular transporters (VNUT1 and VGLUT1) and synaptic vesicle (SV2A) and lysosomal (LAMP3 and Cathepsin-D) markers as described in Materials and Methods. Antibodies were conjugated to fluorescent dyes DyLight488 (green) and DyLight594 (red) prior to astrocyte labeling. To verify the identification of astrocytes and confirm the lack of unspecific immunostaining, cells were also immunolabeled with glial and neuronal markers. After image recordings, electrophysiological characterization of cells was performed as described in Materials and Methods and shown in the Figures 1, S1, and S2 and Table S1. (A–D) The representative two-photon fluorescence images (maximal intensity projections of Z-stack) and results of colocalization analysis, carried out using NIH ImageJ 1.43 software. The correlation between green and red fluorescence (images in the right column) is depicted as a product of the relative differences from the mean (PDM) for each pixel; the pseudocolor PDM images were generated as an output of ImageJ analysis routine. Positive values (bright yellow) are indicative for good co-localization of green and red signals, negative values (blue-violet) indicate segregation, and black color shows the lack of correlation. Note the different extent of scale for PDM values in (A–D). All scale bars in (A–D) are 5 µm. Raw images for panels (A–D) are uploaded as Data S1. (A) Images show good correlation between VNUT1 and SV2A markers. (B, C) Staining of cortical astrocytes for lysosomal markers and correlation between VNUT1 and LAMP3 or cathepsin-D was weaker than for synaptic vesicle marker. Note that VNUT1 was stained using antibodies recognizing the intraluminal epitope and therefore could very likely be labeled as a result of active endocytosis. (D) Images show examples of astrocyte immunostained with VGLUT1; only a fr [file pbio.1001747.s007.tif]

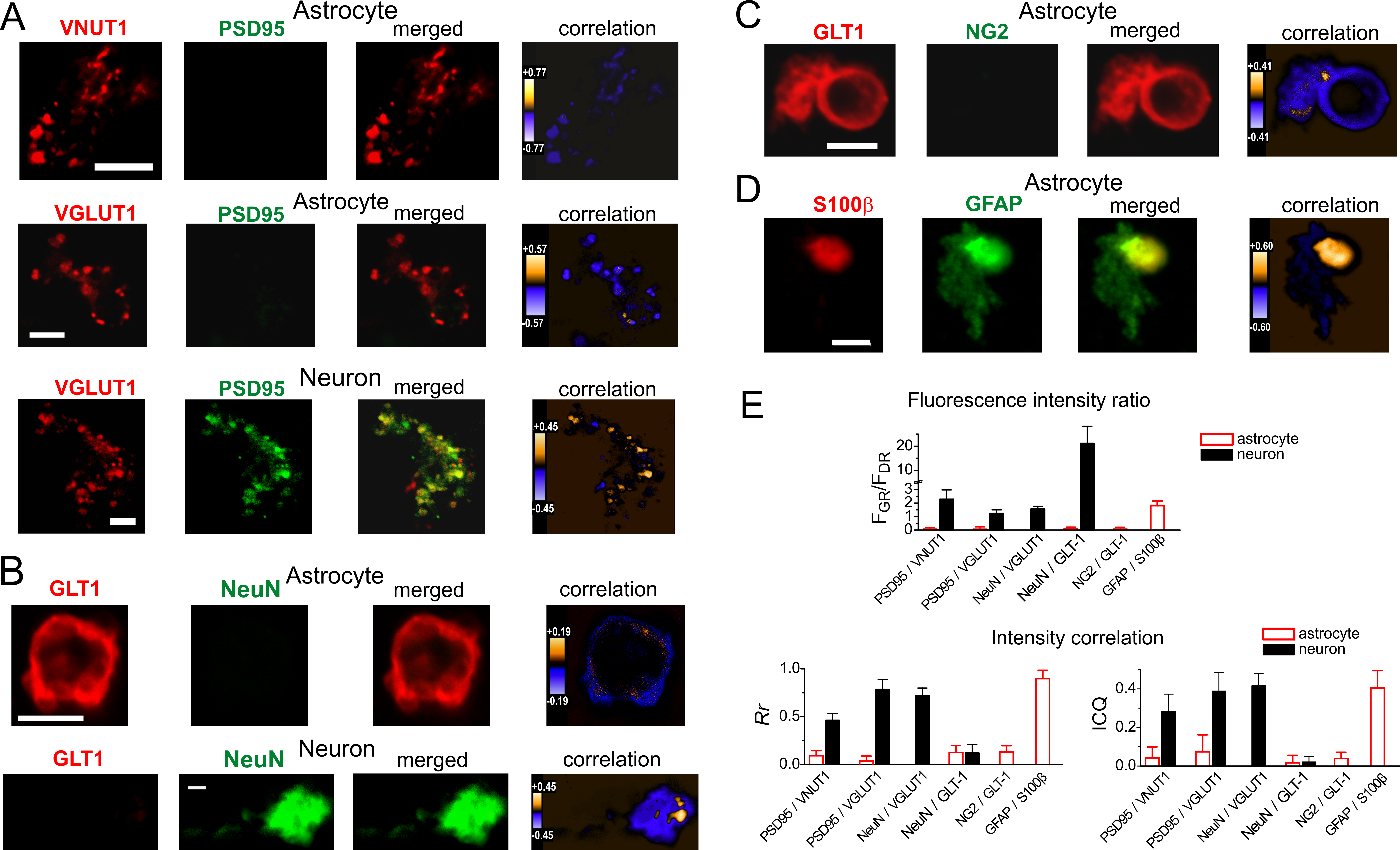

Supplement: Figure S6 — Immunostaining of cortical astrocytes and neurons with synaptic and astroglial markers. Living isolated astrocytes and neurons were cross-stained with various glial and neuronal markers to provide positive and negative control for the immunostaining procedure; the protocols of labeling and analysis were the same as in . (A) The representative two-photon fluorescence images of neocortical astrocytes and pyramidal neuron labeled with antibodies for synaptic marker PSD95 and vesicular ATP and glutamate transporters. Astrocyte and neuron shown in the middle and bottom row were from the same preparation. Note the lack of significant staining of astrocytes with anti-PSD95 contrasting with strong staining of neuron with anti-PSD95 and VGLUT1. (B) Images of astrocyte and neuron stained with antibodies to astrocyte-specific glutamate transporter protein GLT-1 and neuron-specific Neuronal Nuclei protein; cells were from the same preparation. Note the very weak labeling of astrocyte with anti-NeuN and very weak labeling of neuron with anti-GLT-1. (C, D) Good staining of astrocytes with astrocytic markers GLT-1, GFAP, and S100β confirms the efficiency of antibody delivering technique; lack of cross-staining between GLT-1 and NG2 indicates the lack of nonspecific staining. All scale bars in (A–D) are 5 µm. Raw images for panels (A–C) are uploaded as Data S2. (E) The bar diagrams show the pooled data (mean ± SD for the five cells) on the ratio of average fluorescent signal, intensity correlation quotient (ICQ), and Pearson's correlation coefficient (Rr) for pairs of different markers. Although there are certain limitations of usage of immunofluorescence as a quantitative approach, these data clearly demonstrate a qualitative difference in the labeling of neurons and astrocytes. The lack of staining of neurons with astroglial marker GLT-1 and very weak staining of astrocytes with PSD-95 and NeuN strongly suggest the low effect of nonspecific labeling on the immunostainig procedure [file pbio.1001747.s008.tif]

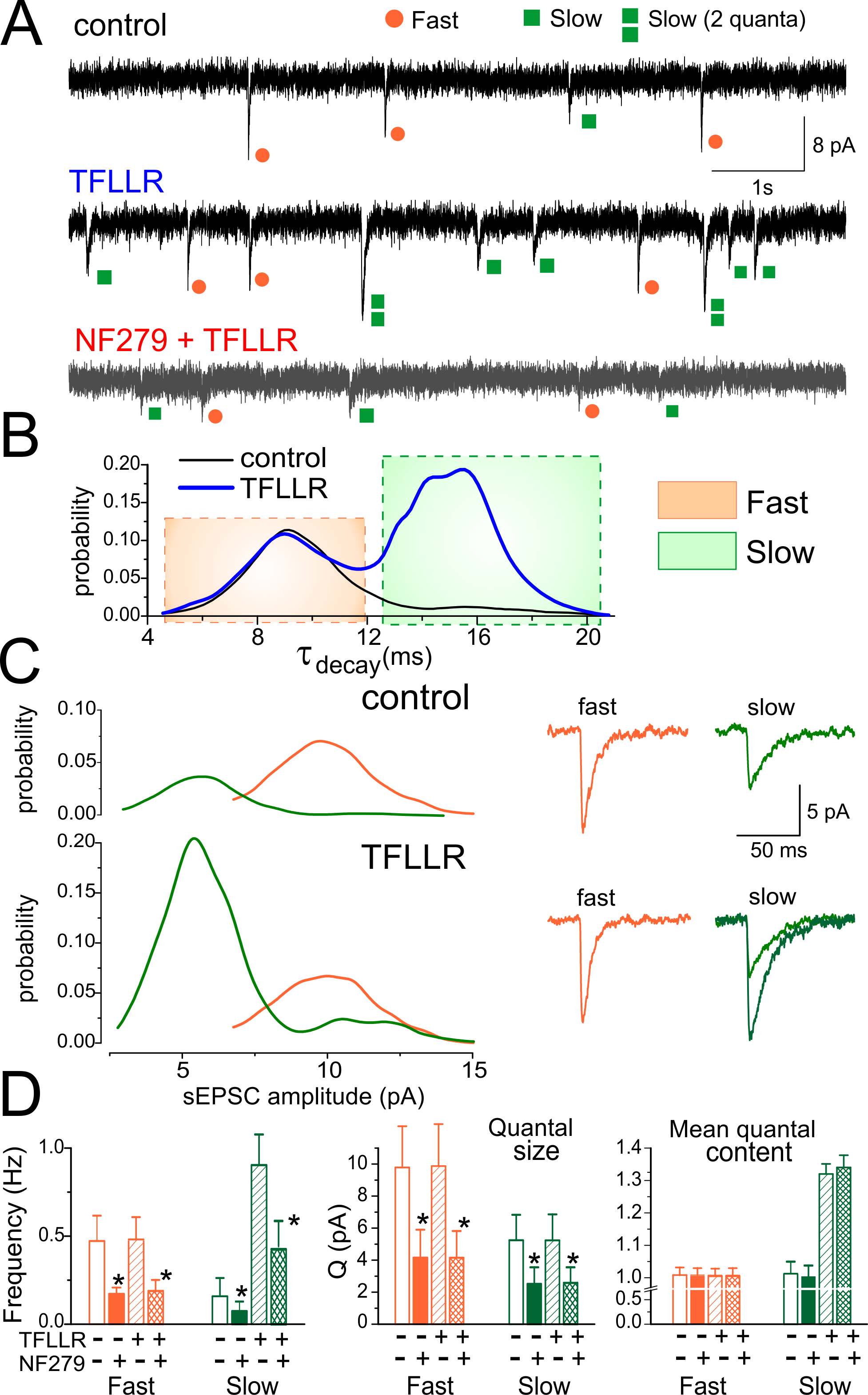

Supplement: Figure S7 — Functional properties of two populations of spontaneous purinergic currents in the neocortical neurons. (A) An example of spontaneous currents recorded in the layer 2/3 pyramidal neocortical neuron wild-type mouse before and after application of glial PAR-1 receptor agonist TFLLR (10 µM) in control and after application of 3 µM NF279. Whole-cell voltage-clamp recordings were performed at a membrane potential of −80 mV in the presence of 100 µM picrotoxin, 1 µM TTX, 50 µM NBQX, and 30 µM D-AP5. Dots indicate the fast (orange) and slow (green) spontaneous currents categorized by the decay time as shown in the panel (B). Both fast and slow spontaneous currents were inhibited by selective P2X receptor antagonist NF279, supporting their purinergic nature. (C) The amplitude distributions of fast and slow purinergic currents were analyzed separately, and the corresponding waveforms (average of 20 traces) are shown in the graph on the left. In the control, the fast sEPSCs exhibited the unimodal amplitude distribution with quantal size of 9.96 pA; slow spontaneous currents had quantal size of 5.54 pA. Application of TFLLR altered neither the frequency nor the amplitude of fast currents but significantly increased the frequency of slow currents. The amplitude distribution of slow currents underwent significant changes showing the secondary peak located at double quantal size (11.2 pA); individual slow events of double quantal size are indicated in panel (A1) by double dots. (D) The pooled data on the frequency, quantal amplitude, and mean quantal content of fast and slow spontaneous currents (mean ± SD for 12 neurons). Stimulation of astrocytes with TFLLR increased the frequency and quantal content of slow currents but did not alter the fast currents. Effects of TFLLR on frequency and quantal content of slow currents were statistically significant with p<0.005 (one-way ANOVA). NF279 significantly reduced the quantal size and frequency of both fast and slow spontaneous current [file pbio.1001747.s009.tif]

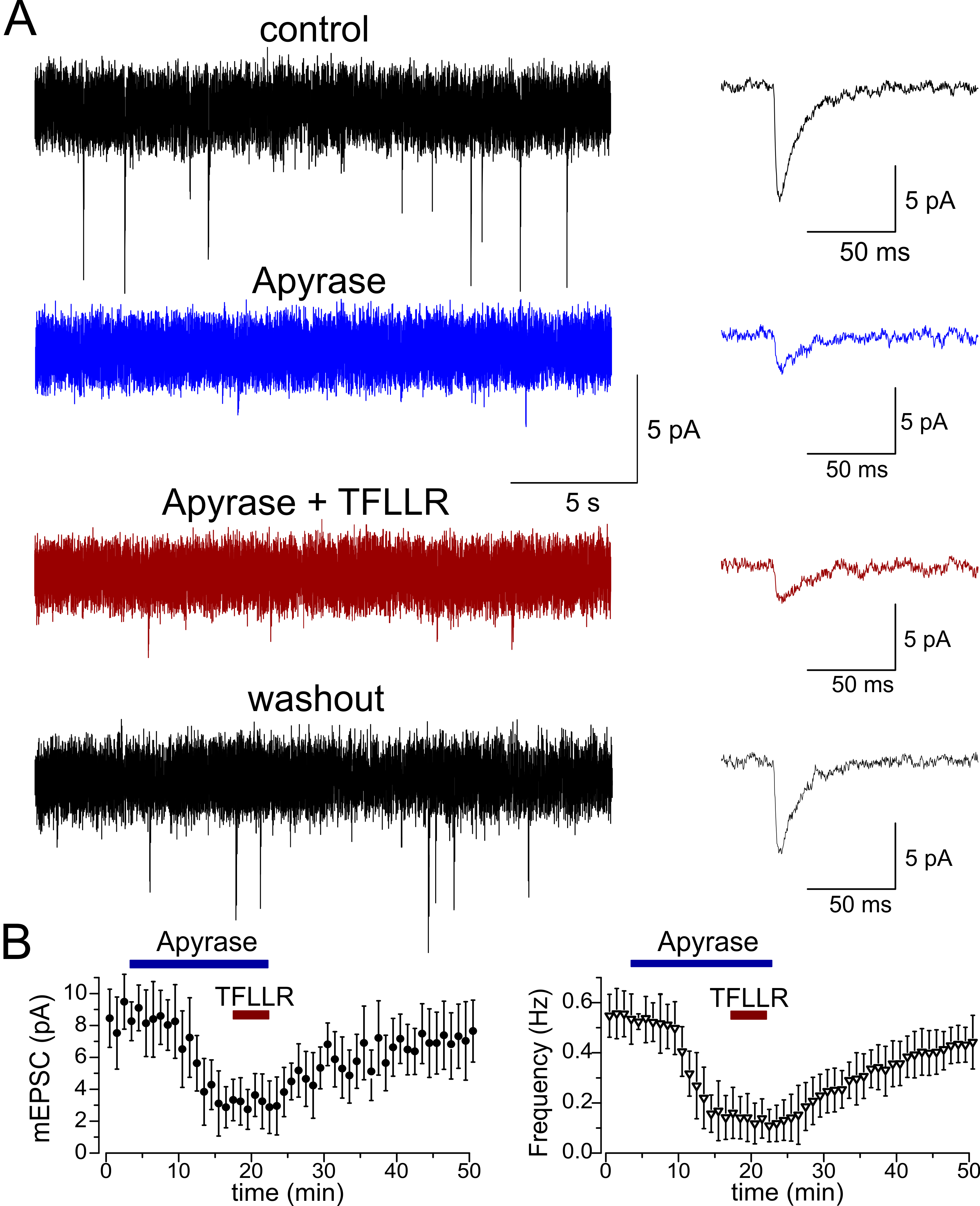

Supplement: Figure S8 — Apyrase inhibits nonglutamatergic sEPSCs in neocortical neurons. (A) Left column shows the representative traces of whole-cell currents were recorded in the layer 2/3 pyramidal cortical neuron at of −80 mV in the presence of 50 µM NBQX, 30 µM D-AP5, and 100 µM picrotoxin (as a control), after application of apyrase (50 U/mL), and after application of PAR1 agonist TFLLR (10 µM) in the presence of apyrase. Right column shows the corresponding waveforms of spontaneous excitatory purinergic currents (average of 25 sEPSCs each). (B) The time course of changes in the average amplitude and frequency of nonglutamatergic sEPSCs recorded in eight pyramidal neurons during application of apyrase (50 U/mL) and TFLLR (10 µM). Dots represent mean ± SD values for mEPCS recorded in a 1-min window. Treatment with apyrase dramatically reduced the baseline amplitude and frequency of nonglutamatergic sEPSCs and prevented the TFLLR-induced burst of sEPSCs. The effects of apyrase on the frequency and amplitude of sEPSCs were statistically significant with p<0.01 both for baseline conditions and TFLLR application. (TIF) [file pbio.1001747.s010.tif]

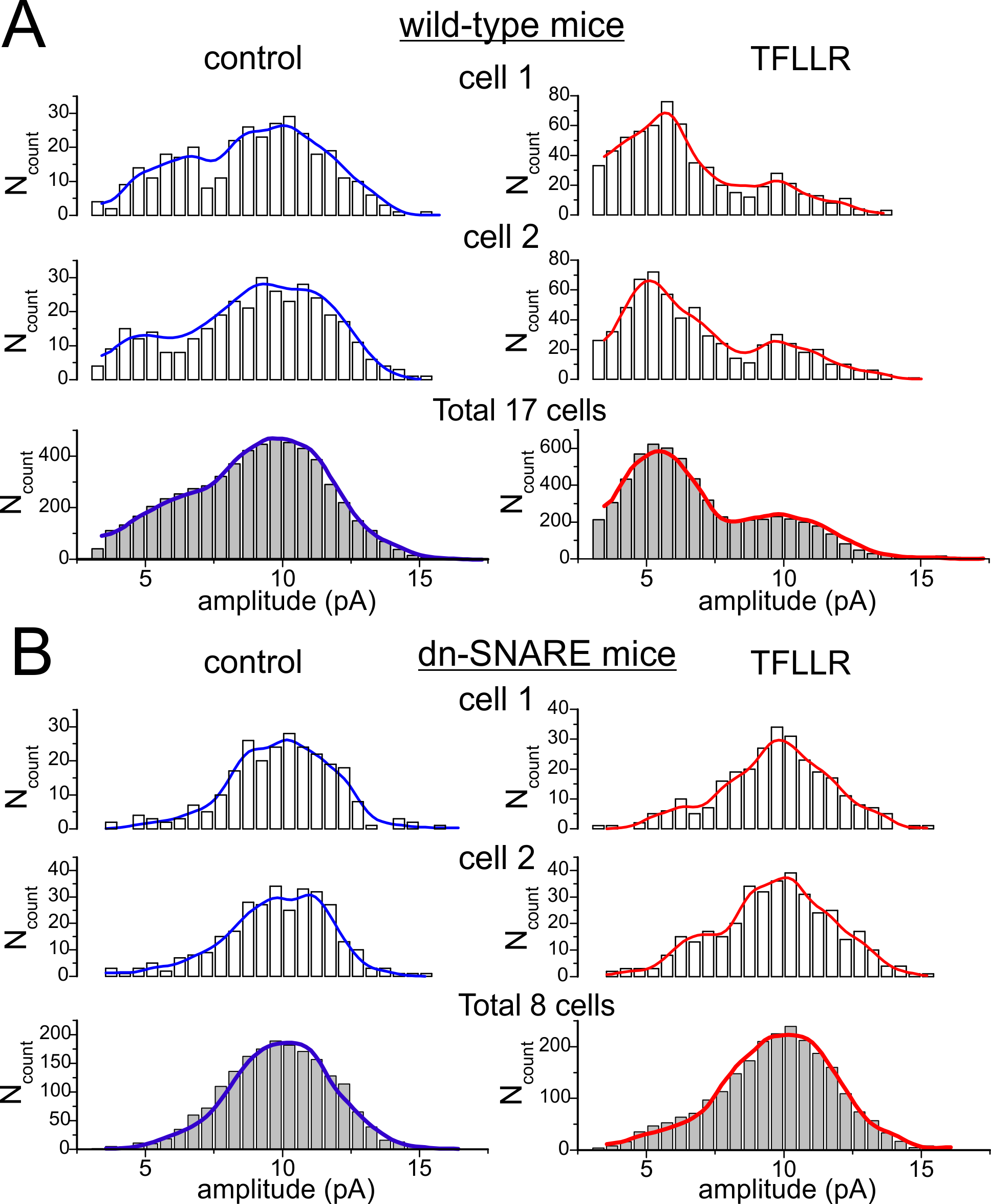

Supplement: Figure S9 — Amplitude histograms for astrocyte-induced purinergic sEPSCs in the pyramidal neurons. (A) Bar histograms show amplitude distributions build using conventional binning for the sEPSCs recorded in pyramidal neurons of wild-type mice as shown in Figure 3A. Two upper rows show examples of amplitude distributions for individual neurons before (left column) and after TFLLR application (right column). Blue and red lines show probability density functions calculated for the same datasets. Third row shows the bin histograms build for the data from all cells tested, and blue and red lines correspond to distributions, depicted in Figure 2D, scaled by the corresponding sample size. Bin size is 1 pA for all histograms. Note that amplitude distributions of sEPSCs recorded from individual wild-type neurons exhibit distinct fraction of small events, and number of small events rises dramatically under action of TFLLR. Good agreement between bin histograms and probability density functions, both in the individual cells and in the whole sample, verifies that the existence of two populations of purinergic currents was not an artifact of analysis procedure. (B) Similar comparison was made for sEPSCs recorded in the neurons of dn-SNARE mice. Note the much lower number of small currents as compared to wild-type neurons. (TIF) [file pbio.1001747.s011.tif]

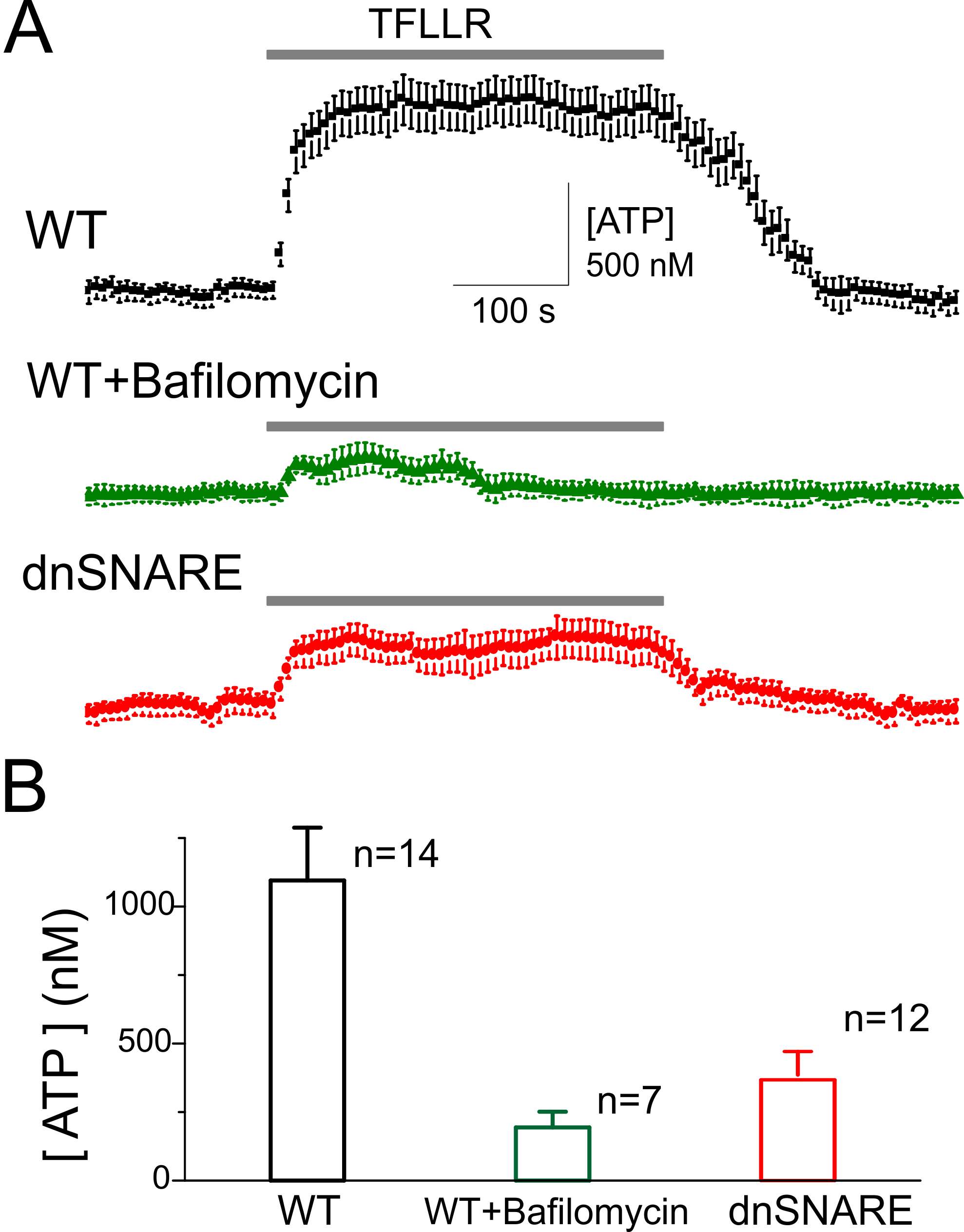

Supplement: Figure S10 — Detection of ATP release in neocortex in situ using microelectrode biosensors. (A) The representative responses of cortical slices of wild-type and dn-SNARE mice to the application of 10 µM TFLLR were recorded using microelectrode biosensors [19],[58] to ATP placed in the layer II/III. Each point represents mean ± SD of ATP concentration measured in the 10 s time window. (B) The pooled data on the peak of ATP transient evoked by application of TFLLR; data shown are mean ± SD for number of experiments as indicated. The difference between the average ATP responses was significant with p<0.01 (one-way ANOVA). In the experiments with bafilomycin A1, slices were pretreated for 2 h with 1 µM of drugs prior to recording. The significant reduction in the ATP response in the cortical slices from dn-SNARE mice and bafilomycin-treated slices strongly supports the vesicular mechanism of ATP release from astrocytes. (TIF) [file pbio.1001747.s012.tif]

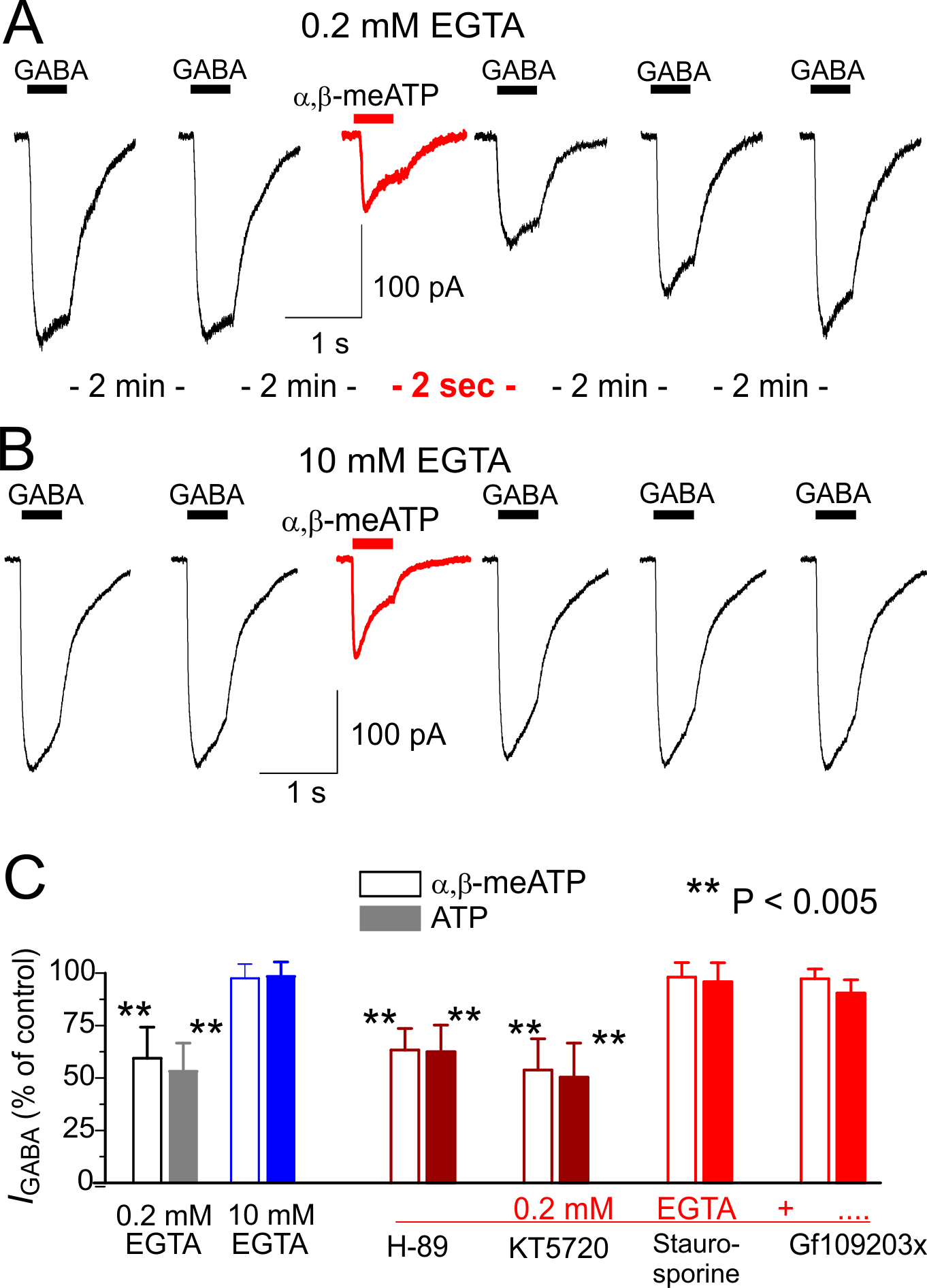

Supplement: Figure S11 — Interaction between P2X and GABA receptors in the mouse neocortical neurons depends on intracellular Ca2+ and activity of protein kinase C. (A) Whole-cell transmembrane currents were recorded in the acutely isolated pyramidal neuron of layer 2/3 of somatosensory cortex at a membrane potential of −80 mV. The currents were elicited by rapid application of GABA (100 µM) and nonhydrolysable ATP analogue α,β-meATP (20 µM) at time intervals indicated below. The cell was perfused with an intracellular solution containing a low concentration of Ca2+-chelator EGTA (0.2 mM). Note the significant decrease in the amplitude of GABA-activated current recorded 2 s after application of P2X receptor agonist. (B) GABAergic and purinergic currents were recorded using the same protocol as in (A) but with intracellular solution containing 10 mM EGTA+1 mM CaCl2 to clamp cytosolic Ca2+ at baseline level. Clamping of cytosolic Ca2+ eliminated the P2X receptor-triggered inhibition of GABA-evoked currents. (C) Pooled data (mean ± SD for eight cells) on the GABA receptor-mediated currents (% of control) recorded 2 s after application of P2X receptor agonists in the presence of high and low concentrations of intracellular EGTA and in the presence of protein kinase inhibitors H-89 (3 µM), KT5720 (200 nM), staurosporine (5 nM), and GF109203x (30 nM). At concentrations used, H-89 and KT5720 were selective for protein kinase A, GF109203x was selective for protein kinase C, and staurosporine was effective on both PKA and PKC. The asterisk (*) indicates the statistically significant (p<0.005) difference in the mean amplitude of GABA-induced current recorded before and after application of P2X receptor agonist. The significant reduction of GABA-evoked currents in the presence of low intracellular EGTA strongly supports its dependence on cytosolic Ca2+ elevation. Note that the reduction in the GABA-evoked current was abolished by inhibitors of PKC but not PKA. There was no statistically significant di [file pbio.1001747.s013.tif]

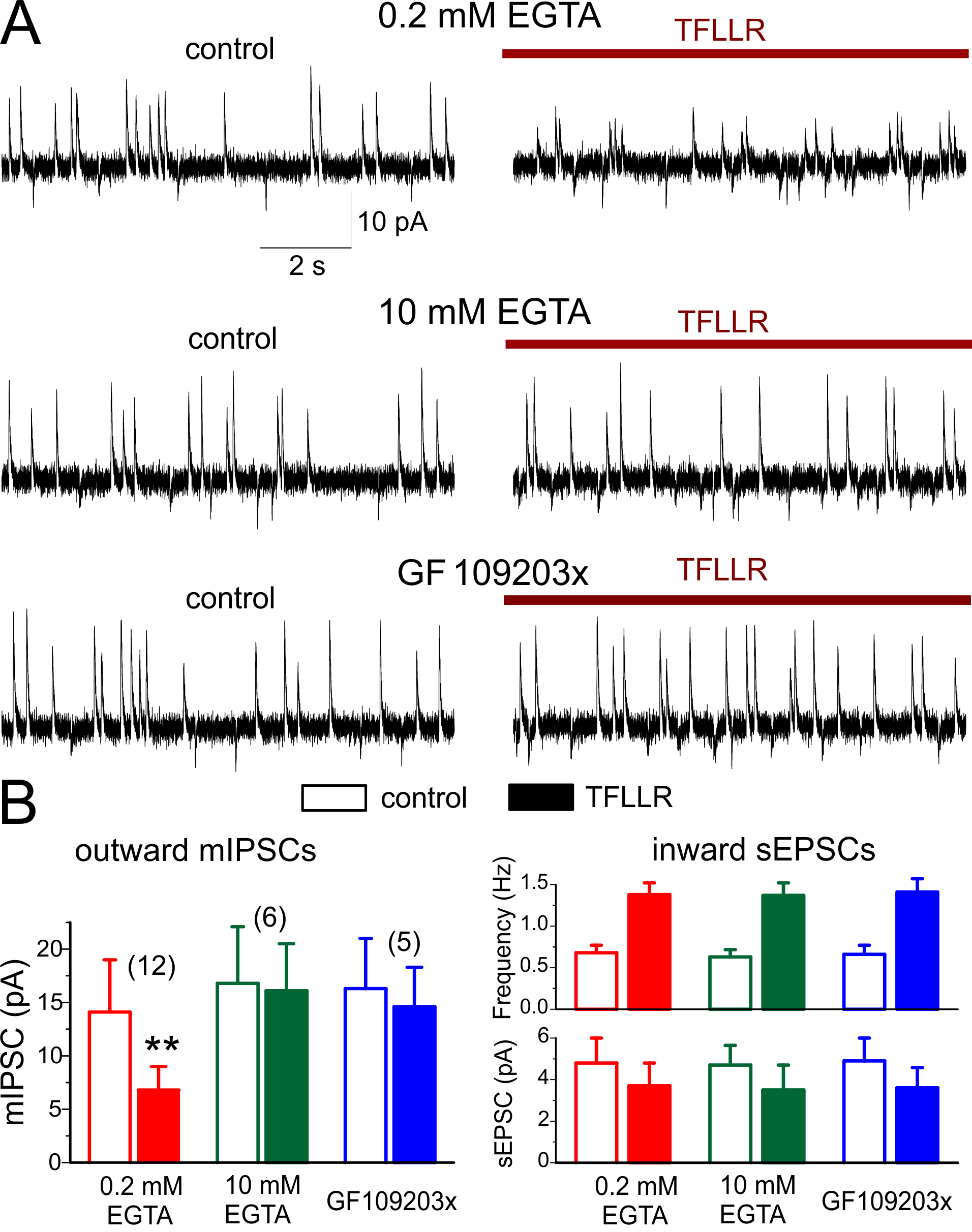

Supplement: Figure S12 — Astrocyte-driven down-regulation of inhibitory transmission in the neocortical neurons depends on intracellular Ca2+ and activity of protein kinase C. (A) Representative mIPSCs recorded in the pyramidal neurons of layer 2/3 before (control) and 20 s after application of PAR-1 receptor agonist TFLLR (10 µM). The whole-cell currents were recorded at a membrane potential of −40 mV using intracellular solution containing either 0.2 mM EGTA (upper traces) or 10 mM EGTA+1 mM CaCl2 to clamp cytosolic Ca2+ at physiological resting level (middle traces) or 0.2 mM EGTA and 100 nM of PKC blocker GF109203x. Extracellular medium contained 1 µM TTX, 50 µM NBQX, and 30 µM D-AP5. Outward currents were mediated by GABA receptors (as shown in Figure 6), and inward currents were mediated by P2X receptors. Activation of cortical astrocytes by TFLLR (as shown in Figure 2, Figure 3, and Figure S3) caused the marked increase in the number of inward spontaneous currents in all recordings. The significant decrease in the amplitude of outward mIPSCs was observed only when intracellular medium contained 0.2 mM EGTA. (B) The graph on the left shows the pooled data (mean ± SD) on the amplitude of mIPSCs recorded at different conditions in the number of neurons indicated in brackets. The asterisk (**) indicates the statistically significant (p<0.005) difference in the mean amplitude of mIPSCs recorded in control and TFLLR for 0.2 mM EGTA. The graphs on the right show changes in the frequency and amplitude of inward currents. The increase in the frequency and decrease in the mean amplitude of sEPSCs occurred due to appearance of a large number of smaller and slower sEPSCs, as shown in Figure 3 and Figure S3. There was no significant difference in the effect of TFLLR on the frequency and amplitude of sEPSCs at different conditions, verifying the independence of astroglial ATP release on intracellular perfusion of neurons. The selective attenuation of GABAergic synaptic currents in the presence of [file pbio.1001747.s014.tif]

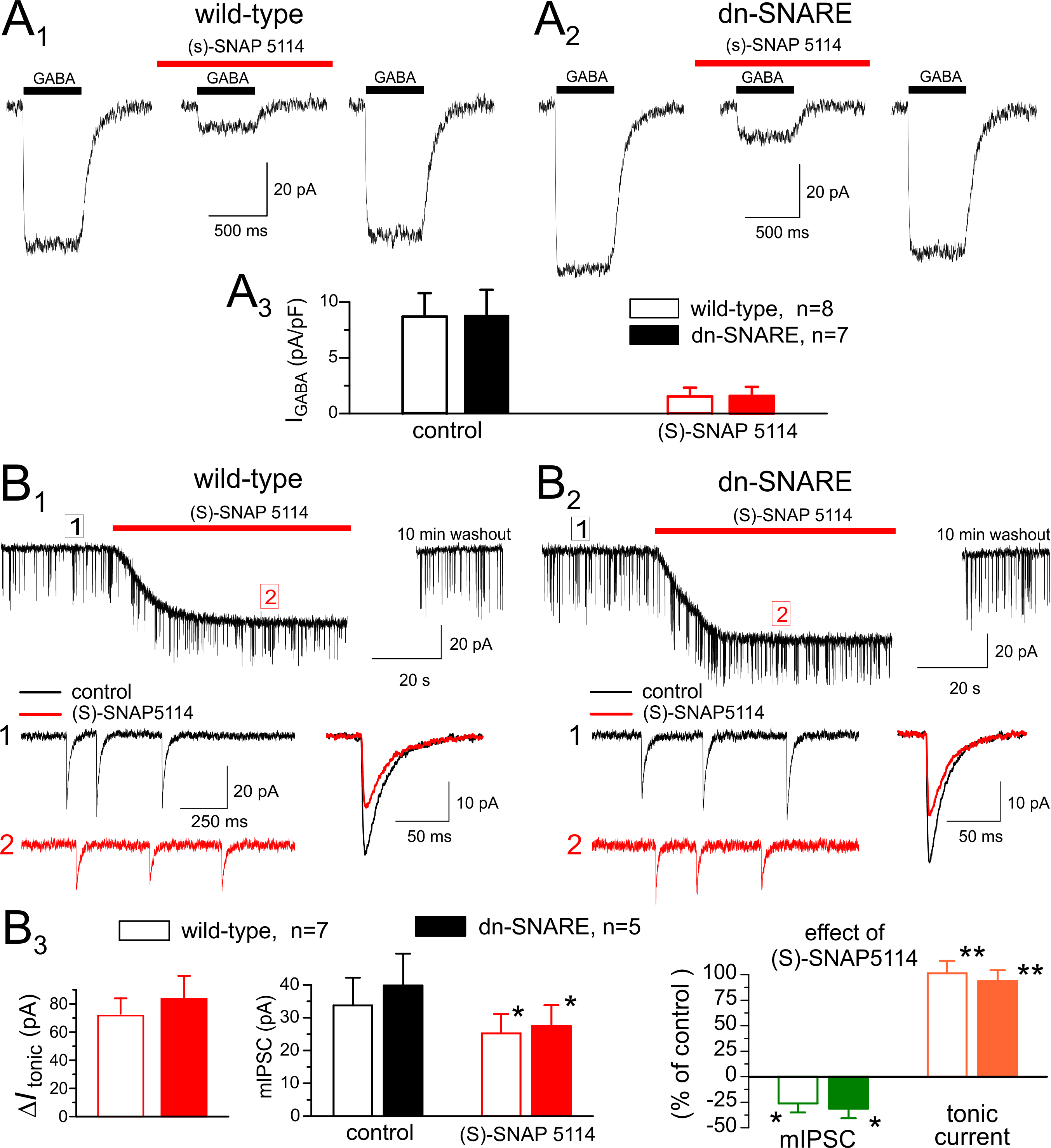

Supplement: Figure S13 — Inhibition of GABA GAT3 transporters decreased the GABAergic mIPSCs and increased the tonic current in neurons of wild-type and dn-SNARE mice. (A) Responses of acutely isolated wild-type (A1) and dn-SNARE (A2) astrocytes were activated by rapid application of 100 µM GABA in the constant presence of 100 µM picrotoxin at a membrane potential of −80 mV. The specific antagonist of glial GAT3 GABA transporters (S)-SNAP5114 (30 µM) was pre-applied 3 min before application of GABA. Diagram in panel (A3) shows pooled data (mean ± SD) on the density of GABA-elicited currents averaged over an indicated number of wild-type and dn-SNARE astrocytes. Significant effect of (S)-SNAP 5114 suggests the major contribution of GAT3 transporters to GABA-evoked currents. There was no significant difference in the GABA transporter-mediated current and effect of (S)-SNAP5114 between the wild-type and dn-SNARE mice. (B1, B2) Upper graph shows the time course of whole-cell transmembrane currents was recorded in the layer 2/3 neocortical pyramidal neurons of wild-type and dn-SNARE mice at −80 mV in the presence of 50 µM CNQX, 30 µM D-AP5, and 10 µM PPADS. Inhibition of astrocytic GAT3 transporters by (S)-SNAP5114 (30 µM) caused an increase in the tonic current, manifested in the downward shift of membrane holding current. Simultaneously, inhibition of GABA transporters caused a decrease in the amplitude of synaptic mIPSCs. Examples of mIPSCs, recorded at moments indicated, and average waveform of phasic GABAergic current (average of 25 mIPSCs) are shown in the inlays below. (B3) Diagrams show the pooled data on, from left to right, the change in the magnitude of membrane holding current, the amplitude of synaptic mIPSCs, and relative changes in the amplitudes of synaptic and tonic GABAergic currents. Data are shown as mean ± SD for the number of neurons indicated. The asterisks indicate the statistical significance of difference from the control values, (*) p<0.05 and (**) p<0.01. Note the opp [file pbio.1001747.s015.tif]

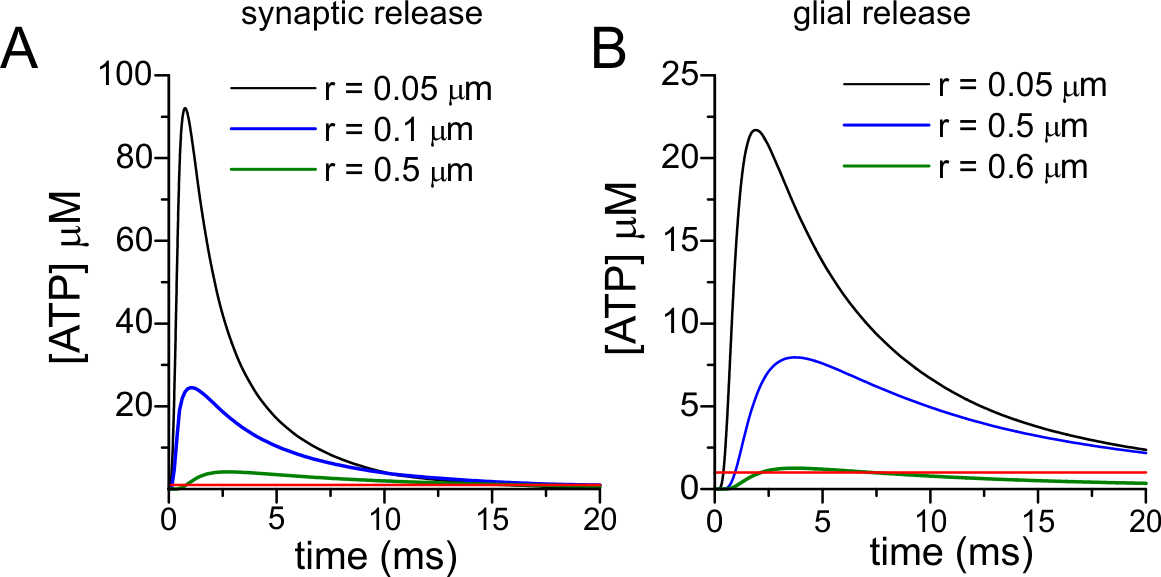

Supplement: Figure S14 — Computer simulation of ATP diffusion. To estimate the time course of ATP concentration in the extracellular space, we adapted a computer model previously used to simulate spillover of glutamate [53],[54]. The model was based on a simplified description of neurotransmitter diffusion suggested by Rusakov and Kullman [53]. The movement of ATP molecules released from the nerve terminal (A) was considered a free two-dimensional diffusion inside of the cylindrical synaptic cleft of a radius of 100 nm and height of 20 nm and a three-dimensional diffusion in the porous medium outside of the synaptic cleft. Movement of ATP after release from the glial site (B) was also calculated as a three-dimensional diffusion in the porous medium with the following parameters: diffusion coefficient D = 0.2 µm/ms (50% lower than for glutamate diffusion), free volume factor 0.12, and tortuosity factor of 1.34. To account for the quick breakdown of ATP by ectonucleotidases, the kinetic scheme for transporter uptake was replaced by Michaelis–Menten kinetics of ATP->ADP conversion with Vmax and KM values of 2.2 µM/s and 33 µM [63]. In both cases, the conservative estimate (i.e., longer travel) of linear spine density as 1 spine/µm [46] was used. (A) Graph shows simulated time course for ATP concentration after release of 1,000 molecules from the vesicle at the centre of synaptic cleft (r = 0) for the different distances: middle of synaptic cleft (r = 0.05 µm), edge of synaptic cleft (r = 0.1 µm), and middle of typical intersynaptic distance (0.5 µm).(B) Graph shows simulated time course for ATP concentration after release of 1,000 molecules from ectopic (glial) site located at the middle of the intersynaptic distance, for the following locations: vicinity of the release site (r = 0.05 µm), edge of the nearest synapse (r = 0.5 µm), and centre of nearest synapse (r = 0.6 µm). Red horizontal line in both graphs indicates a minimal level of ATP that can reliably activate P2X2 and P2X4 receptors, s [file pbio.1001747.s016.tif]

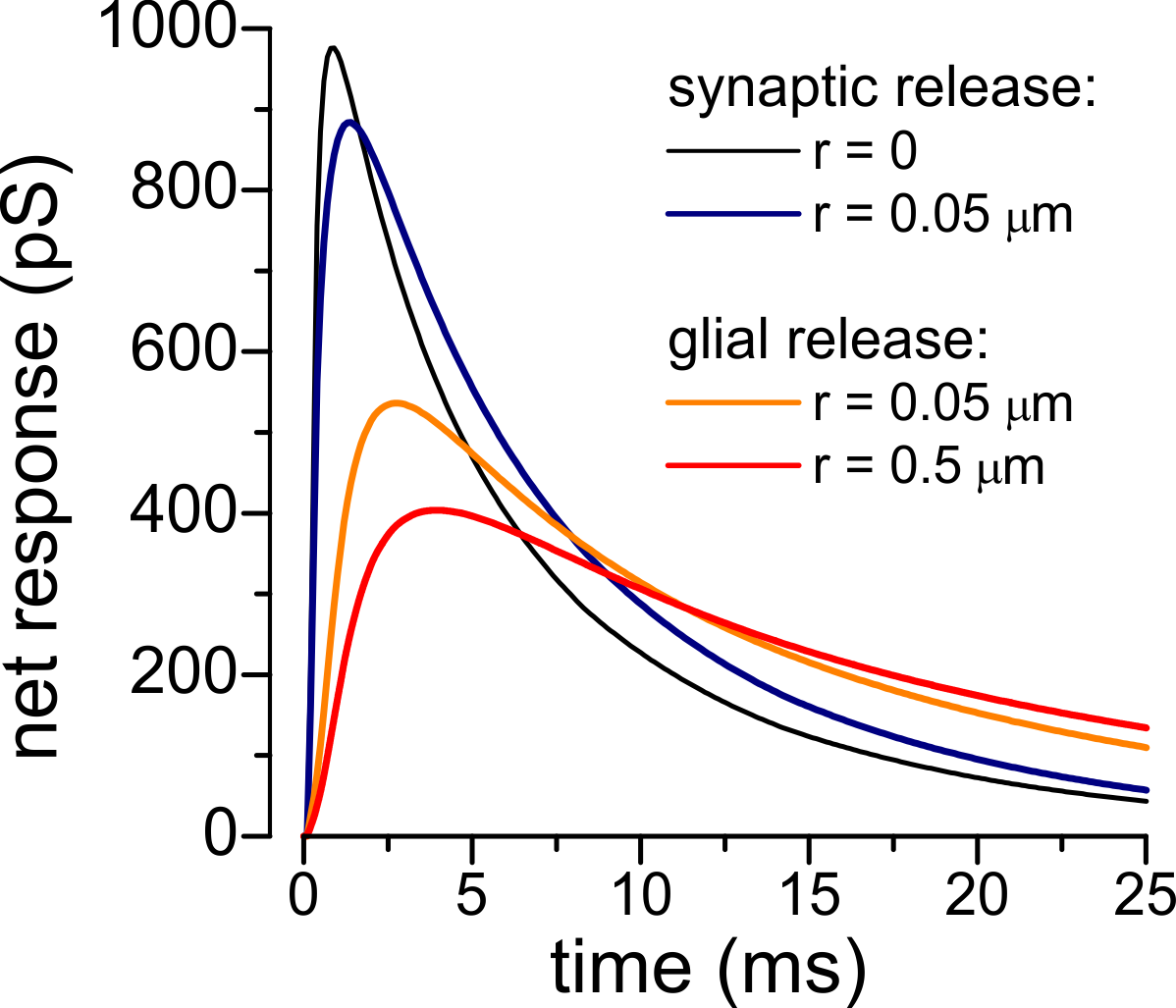

Supplement: Figure S15 — Computer simulation of P2X receptor response after synaptic and ectopic release of ATP. The simulation of net response (calculated as a net conductance) of synaptic and extrasynaptic P2X receptors located within 0.5 µM of centre of synaptic cleft. The same model of ATP release and diffusion was used as in Figure S14. To account for preferential location of the P2X receptor on the periphery of the synapse, their density was set as 5×10^−3^/nm2 within the radius of r = 0.1±0.01 (periphery of dendritic spine) nm and 5×10^−4^/nm2 elsewhere. This is a conservative estimate since maximal density is set at less than 1 receptor per 10 nm. The equal proportion of P2X2 and P2X4 receptors was assumed. Kinetic model of receptor activation used in [54] was replaced by the simplified model of P2X2 receptor kinetics [42],[66] and was used for both receptors, with an activation time of P2X4 receptors decreased to 8.8 ms accordingly to [67]; maximal conductance and open probability for the P2X2 and P2X4 receptors were set correspondingly as 21 and 9 pS and 0.6 and 0.2 [39],[64],[67]. Synaptic release (black and blue lines): the response to release of 1,000 ATP molecules from the vesicle at the central part of (r = 0) and peripheral part of synaptic cleft (r = 0.05). Glial release (orange and red lines): the response to vesicular release of 1,000 ATP molecules from the glial site located at the middle of the intrasynaptic space of (r = 0.05) and near the peripheral part of synaptic cleft (r = 0.5). The response to synaptic release does not show strong dependence on distance to release site (A), resulting from the saturation of receptors. Response to glial release does not show steep dependence either, and this can be explained by the decrease in the ATP concentration at r = 0.5 (edge of synapse) that is compensated by an increase in the density of receptors. Note that the simulated P2X responses to glial release have smaller amplitudes and slower kinetics than responses to the synaptic r [file pbio.1001747.s017.tif]

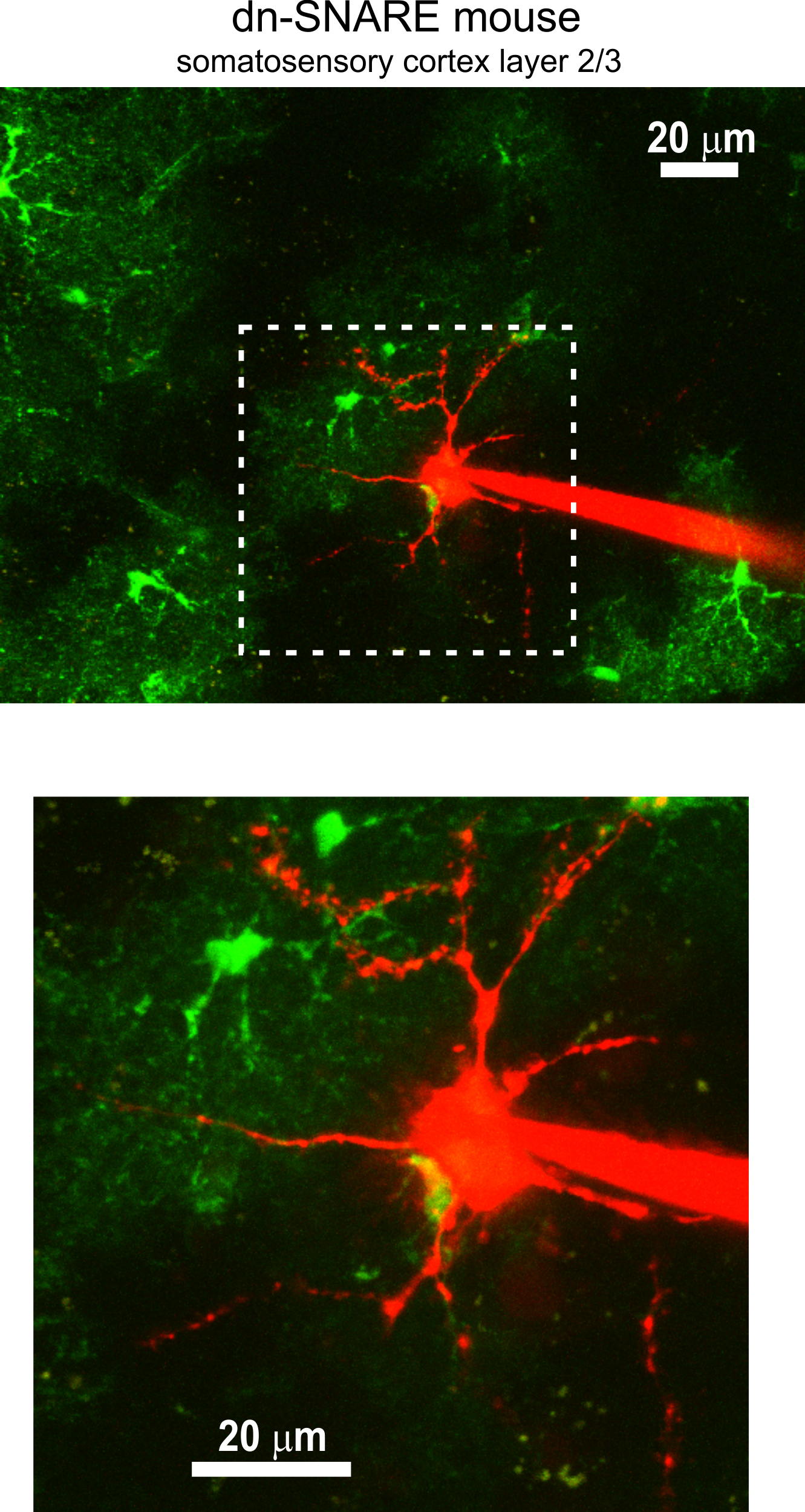

Supplement: Figure S16 — Whole-cell recording from pyramidal neocortical neuron of dn-SNARE mouse. Images show the typical outline of experiments described in Figures 6–8. The two-photon image of EGFP fluorescence of dn-SNARE astrocytes (maximal projection of Z-stack) is merged with the fluorescent image of the pyramidal neuron perfused with intracellular solution containing fluorescent dye Texas Red (20 µM). Note the three green dn-SNARE-expressing astrocytes in the immediate vicinity of pyramidal neuron. (TIF) [file pbio.1001747.s018.tif]
