# Supplementary material for: Exocytosis of ATP From Astrocytes Modulates Phasic and Tonic Inhibition in the Neocortex
Source: PLoS Biol. 2014 Jan 7;12(1):e1001747. doi: 10.1371/journal.pbio.1001747 (PMC3883644; doi:10.1371/journal.pbio.1001747)
Supplement: Table S1 — Properties of WT and dn-SNARE astrocytes. (PDF) [file pbio.1001747.s019.pdf]

Table S1
Properties of acutely isolated neocortical astrocytes.

Characterization of cortical astrocytes
Recordings were performed on astrocytes of somato-sensory cortex of dnSNARE transgenic mice [12,20] and their wild-type littermates (WT). Initial experiments to investigate release of ATP from astrocytes were also performed in transgenic mice expressing enhanced green fluorescent protein (EGFP) under the control of the glial fibrillary acidic protein (GFAP) promoter [61].
Astrocytes were initially identified by their morphology under DIC observation and EGFP fluorescence (astrocytes from dnSNARE and GFAP-EGFP mice). At the end of all experiments, identification of cortical astrocytes was confirmed by their functional characterization including low input resistance, lack of voltage-gated Na^+-conductance, large K^+-conductance, large conductance mediated by glutamate transporters, NMDA receptor-mediated current lacking Mg^{2+}-block [24]. Glutamate transporter-mediated currents were induced by application of 100μM glutamate in presence of CNQX (30μM) and D-AP5 (50 μM); NMDA receptors were activated by application of 20 μM NMDA; GABA transporters were activated by application of 100 μM GABA. Examples of responses are shown in Figures 1 and S7,

	EGFP-GFAP astrocytes (n=15)*	Wild-type astrocytes (n=53)*	dn-SNARE astrocytes (n=30)*
Diameter, μm	5.7 ± 1.2	5.5 ± 1.4	5.6 ± 1.3
Input Resistance (MΩ)	79 ± 23	71 ± 25	74 ± 27
Resting potential (mV)	-82.5±2.4 (n=8)	-81.9±2.6 (n=9)	-83.1±2.7 (n=8)
Slow-Inactivating potassium current (pA/pF) evaluated by response to membrane voltage jump from -80 to +40 mV	407 ± 66	416 ± 59	421±63
Kir channel -mediated potassium current (pA/pF) evaluated by Ba^{2+}-sensitive component of response to membrane voltage jump from -50 to -130 mV	310 ± 49 (n=8)	294 ± 47 (n=6)	283±51 (n=6)
NMDA receptor mediated current at -80 mV (pA/PF)	10.9 ± 2.4	11.17± 2.9	12.1± 2.7
Glutamate transporter current at -80 mV (pA/pF) evaluated as response to 100 μM Glu in presence of CNQX and APV)	4.7± 1.5	5.2± 1.6	5.0± 1.3
Inhibition of glutamate transporter current by 300 nM TFB-TBOA (%)	96.1± 3.4 (n=6)	94.9± 4.8 (n=6)	96.9± 3.7 (n=5)
GABA transporter current at -80 mV (pA/pF) (evaluated as response to 100 μM GABA in presence of picrotoxin	4.9± 1.9 (n=5)	5.4± 1.5 (n=15)	5.2± 1.6 (n=12)

***Total number of cells tested**, in some experiments number of cells was different (as specified)
