# Supplementary material for: Exocytosis of ATP From Astrocytes Modulates Phasic and Tonic Inhibition in the Neocortex
Source: PLoS Biol. 2014 Jan 7;12(1):e1001747. doi: 10.1371/journal.pbio.1001747 (PMC3883644; doi:10.1371/journal.pbio.1001747)
Supplement: Table S2 — Action of bafilomycin A1 on phasic purinergic currents in the neocortical neurons in situ. (PDF) [file pbio.1001747.s020.pdf]

**Table S2**

**Bafilomycin inhibits release of ATP from cortical astrocytes *in situ*.**

Spontaneous excitatory purinergic currents (mEPSCs) were recorded in the pyramidal cortical neuron at of -80 mV in presence of 50 µM NBQX, 30µM D-AP5 and 100 µM picrotoxin in control and bafilomycin-treated neocortical slices. Treatment with bafilomycin A1 dramatically inhibited the baseline frequency of purinergic mEPSCs and prevented the TFLLR-induced burst of purinergic mEPSCs. The effects of bafylomicin on the frequency and amplitude of mEPSCs were statistically significant with $P < 0.01$ both for baseline conditions and TFLLR application.

| | Control (n=12) | Bafylomicin (n =6) |
|---|---|---|
| **Frequency of purinergic mEPSCs, Hz** | | |
| Baseline | 0.63 ± 0.08 | 0.053 ± 0.019 |
| After application of TFLLR | 1.37 ± 0.14 | 0.052 ± 0.017 |
| **Amplitude of purinergic mEPSCs, pA** | | |
| Baseline | 8.87 ± 1.78 | 2.75 ± 0.85 |
| After application of TFLLR | 5.72 ± 1.44 | 2.78 ± 0.69 |
