# Supplementary material for: Exocytosis of ATP From Astrocytes Modulates Phasic and Tonic Inhibition in the Neocortex
Source: PLoS Biol. 2014 Jan 7;12(1):e1001747. doi: 10.1371/journal.pbio.1001747 (PMC3883644; doi:10.1371/journal.pbio.1001747)
Supplement: Text S1 — Limitations of immunostaining of living astrocytes. (PDF) [file pbio.1001747.s021.pdf]

Limitations of immunostaining of living astrocytes

We would like to note that we performed immunostaining of astrocytes with vesicular markers merely to provide a supporting evidence of their ability to exocytose ATP which was demonstrated by several lines of physiological experiments (Figures 1-4). The main advantage of immunolabeling of living acutely isolated astrocytes was opprtunity to stain vesicular proteins at intraluminal epitope exposed during spontaneous physiological exocytosis. The 2-photon microscopy was chosen because of less photo-damage produced in astrocytes.

As with other immunostaining approaches, the data obtained in living astrocytes may be affected by non-specific antibody binding. Also, rather specifically for staining of living cells, antibodies could be taken into the cell due to non-specific lysosomal endocytosis. One should note, however that conventional approach of immunostaining of fixed slices has its own limitations and can also be prone to unspecific labeling. For instance, recent work [32] has demonstrated that anti-VGLUT1-3 antibodies can label astrocytes of VGLUT1-3 knock-out mice.

To decrease the probability of unspecific labeling, we used rather low concentration of antibodies and relatively short the exposure time to 60 min. We also provide few sets of control data, including cross-staining of astrocytes and neurons in the same preparation with antibodies to neuronal markers neuroregulin and PSD95 and astrocytic markers GLT-1, S100β and GFAP. The results of cross-staining, presented in the Figure S6, support the specificity of antibody labeling in our preparations. Still, when interpreting the immunostaining data shown in Figures S5 and S6, one should take into account several technical issues, pointed out below.

1) Vesicular ATP transporter VNUT1 was immunostained using antibodies recognizing the intraluminal epitope and thereby could be labeled mainly as a result of vesicular endocytosis.
2) The anti-VGLUT1 (clone McKA1) antibodies, used in our experiments, were raised against the peptide similar to the C-terminal part of mouse VGLUT1 and, very likely, would bind the VGLUT1 protein at the cytoplasmic site. The anti-VGLUT1 antibodies were not conjugated with BioPorter reagent , so the most feasible explanation of punctate staining of astrocytes (Figure S5) and neurons (Figure S6) with these antibodies could be non-specific uptake by endocytosis of synaptic vesicles followed by release into the cytosol after vesicle degradation. After then, antibodies could label the cytoplasmic site at VGLUT1 protein inserted into recycled or *de novo* formed vesicles. Since VGLUT1 can undergo rather rapid trafficking and recycling [1], it is conceivable that sufficient staining of VGLUT1-containing vesicles could occur during 60 min exposure period used in our protocol. This notion is supported by rather good VGLUT-1 staining and good co-localization with PSD95 marker observed in neurons.
However, the complicated pathway leading to binding of antibody to VGLUT-1 protein could significantly decrease the efficiency of labeling as compared to immunostaining of astrocytes for VNUT1, directly labeled at intralumenal epitope during endocytosis. So, our data on weaker staining of astrocytes with VGLUT1 (Figure S5) should be interpreted with a great caution .
3) Acute isolation of neurons with the aid of vibrodissociation can retain functional synaptic boutons which can undergo spontaneous exocytosis [2]. Thus, it is conceivable that anti-PSD95 marker would stain the postsynaptic density of the recorded neuron and anti-VGLUT1 antibodies could be taken by synaptic vesicles of pre-synaptic terminals. However, the separation of stained pre- and postsynaptic sites could not be fully resolved by 2-Photon microscopy due to small size of excitatory synapses in the pyramidal neurons. This could explain the good correlation of PSD95 and VGLUT1 staining in neurons. Neurons, most likely, retain synaptic boutons due to structural links between post and presynaptic membranes (i.e. cadherin molecules). It is hardly unlikely that thin astrocytic processes, that enwrap synapses, can overcome these links and pull synaptic boutons out during isolation. This notion is supported by the lack of immunostaining of astrocytes with anti-PSD95 antibodies.
4) Possibly, antibodies could be taken into living astrocytes during spontaneous lysosomal endocytosis

thereby leading to non-specific staining. However, this mechanism does not appear to bring the main contribution to the staining of astrocytes in our experimental conditions. Otherwise, one could expect to observe the punctate labeling of astrocytes with GFAP, GLT-1 and other non-vesicular markers and observe good correlation between neuronal and glial markers. This was not the case.

5) Although we observed co-localization of VNUT1 with both synaptic vesicle and lysosomal markers, the correlation parameters and intensity ratio differed considerably among VNUT1 / SV2, VNUT1 / CatD and VNUT1 / LAMP-3 pairs (Figure S5E). Also, we observed more vesicles labeled for VNUT1 than for SV2, LAMP-3 and especially for CatD, as evidenced by number of green spots in Figures S5A-C. This arguers against the significant impact of non-specific endocytosis on antibodies uptake. The most feasible explanation for cross-staining between VNUT1 and SV2 and VNUT1 and CatD/LAMP-3 could be the presence of vesicular ATP transporter both in the synaptic-like vesicles and lysosomes, which supports the data reported previously [22-26].
6) The Cathepsin-D, which was used a second lysosomal marker, is a soluble lysosomal protein. Upon full fusion of lysosome with plasma membrane, it can be released into the extracellular space which would prevent the staining of lysosomes with anti-CatD antibodies. However, astrocytic lysosomes can also undergo kiss-and-run events [16]. Our sniffer-cell data also argue against contribution of full fusion events to the release of ATP, that would have caused massive and prolonged responses in the sniffer cells (as discussed in the main text). Thus it is possible that at significant proportion of astrocytic lysosomes can undergo kiss-and-run exocytosis during which antibodies could bind Cathepsin-D inside the opened vesicle and could be taken back after then. Dependence of anti-CatD labeling on kiss-and-run exocytosis agrees with less efficient staining as compared to membrane lysosomal marker LAMP-3 (Fig. S5B,C,E).

References:

1. Santos MS, Li H, Voglmaier SM (2009) Synaptic vesicle protein trafficking at the glutamate synapse. Neuroscience 158: 189-203.
2. Duguid IC, Pankratov Y, Moss GW, Smart TG (2007) Somatodendritic release of glutamate regulates synaptic inhibition in cerebellar Purkinje cells via autocrine mGluR1 activation. J Neurosci 27: 12464-12474.
